# Synthesis and antibacterial activity of Schiff bases and amines derived from alkyl 2-(2-formyl-4-nitrophenoxy)alkanoates

**DOI:** 10.1007/s00044-015-1397-6

**Published:** 2015-07-15

**Authors:** Agata Goszczyńska, Halina Kwiecień, Karol Fijałkowski

**Affiliations:** Department of Organic Synthesis and Drug Technology, West Pomeranian University of Technology, Szczecin, Al. Piastów 42, 71-065 Szczecin, Poland; Department of Immunology, Microbiology and Physiological Chemistry, West Pomeranian University of Technology, Szczecin, Al. Piastów 45, 70-311 Szczecin, Poland

**Keywords:** Amines, Antibacterial activities, Esters, 2-(2-Formylphenoxy)alkanoic acids, Reduction, Reductive amination, Schiff bases

## Abstract

A series of novel Schiff bases and secondary amines were obtained in good yields, as a result of the reductive amination of alkyl 2-(2-formyl-4-nitrophenoxy)alkanoates with both aniline and 4-methoxyaniline under established mild reaction conditions. Sodium triacetoxyborohydride as well as hydrogen in the presence of palladium on carbon were used as efficient reducing agents of the Schiff bases, in both direct and stepwise reductive amination processes. The Schiff bases, amines, and amine hydrochlorides were designed as potential antibacterial agents, and structure–activity relationship could be established following in vitro assays against Gram-positive and Gram-negative bacteria. The minimal inhibitory concentration and zone of inhibition were also determined. In these tests, some of Schiff bases and secondary amine hydrochlorides showed moderate-to-good activity against Gram-positive bacteria, including *S. aureus*, *M. luteus*, and *S. mutans*.

**Electronic supplementary material:**

The online version of this article (doi:10.1007/s00044-015-1397-6) contains supplementary material, which is available to authorized users.

## Introduction

The major problem in the effective antibacterial treatment is increasing resistance of microorganisms to currently available antimicrobial drugs. Therefore, the development of novel antimicrobial drugs is an active area of research. Most of compounds bearing an azomethine group exhibit antimicrobial (da Silva *et al.*, 2011; Mohini *et al.*, 2013; Shi *et al.*, 2007), antioxidant, and antiproliferative properties (Cheng *et al.*, 2010). Schiff bases, such as nitrofurantoin or nifuroxazide, are commonly applied in medicine as antibacterial agents (Sztanke *et al.*, 2013). Additionally, a variety of phenoxyalkanoic acid derivatives are also known to possess a wide range of bioactivities (Hullar and Failla, 1969; Pattan *et al.*, 2009; Kumar and Kumaresan, 2012), and some of the Schiff bases derived from 2-formylphenoxyacetic acids exhibit antibacterial properties (Bala *et al.*, 2010; Iqbal *et al.*, 2007). Further, aromatic secondary amines, as well as their salts, are also known to possess antimicrobial activity (Kitahara *et al.*, 2004; Singh *et al.*, 2011). Finally, secondary amines containing an aromatic nitro group exhibit an arginase inhibitory effect on vascular smooth muscle cell proliferation (Curtis *et al.*, 2013).

A widely useful method for the synthesis of amines is reductive amination, which involves the reaction of aldehydes and ketones with ammonia or primary/secondary amines in the presence of a selective reducing agent (Tarasevich and Kozlov, 1999; Gomez *et al.*, 2002). This process is considered *direct* when a carbonyl compound and an amine are mixed together with a reducing agent in a single operation. On the other hand, a *stepwise* reductive amination involves the pre-formation of the intermediate imine, followed by reduction in a separate step (Abdel-Magid *et al.*, 1996). A wide variety of reducing agents have been utilized for reductive amination; however, two methods have been used most commonly. The first method involves catalytic hydrogenation with platinum, palladium, ruthenium, cobalt or nickel catalysts (Klyuev and Khidekel, 1980; Petrisko and Krupka, 2005; Tripathi *et al.*, 2008). The second method utilizes metal hydride reagents, mainly sodium borohydride (Panfilov *et al.*, 2000), sodium triacetoxyborohydride (Abdel-Magid and Mehrman, 2006; Gribble, 2006), sodium or lithium cyanoborohydride (Borch *et al.*, 1971; Grenga *et al.*, 2009), and sodium borohydride modified with numerous polyvalent metal salts (Saxena *et al.*, 2000; Saidi *et al.*, 2007; Neidigh, *et al.*, 1998) or activated by acids (Cho and Kang, 2005; Alinezhad *et al.*, 2010).

Here, we report the synthesis of a series of Schiff bases and amines that were designed as potential antimicrobial agents. The synthesis involves the chemoselective reaction of primary amines with alkyl 2-(2-formyl-4-nitrophenoxy)alkanoates yielding Schiff bases bearing intact the ester group, as well as further reduction of the Schiff bases to the corresponding amino esters. Further, we have performed antibacterial screening of the obtained compounds against Gram-positive and Gram-negative bacteria and analyzed the influence of the electron-donating substituents such as methoxyl and amino groups in the phenyl rings, as well as length of hydrophobic side chain on the antibacterial activities.

## Results and discussion

### Chemistry

**Scheme 1:**
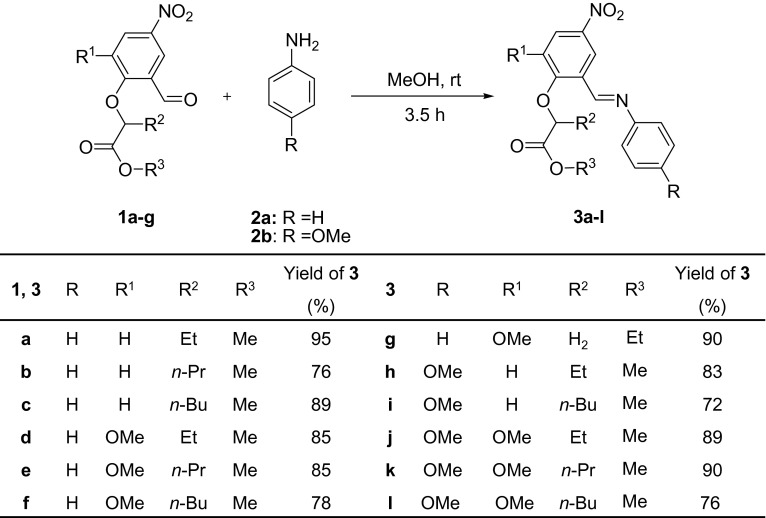
Synthesis of Schiff bases from formyl esters **1a**–**g** and aniline (**2a**) or 4-methoxyaniline (**2b**)

The desired alkyl 2-(2-formylphenoxy)alkanoates **1a**–**g** were obtained in high yield by the condensation of adequately substituted 2-hydroxybenzaldehydes with alkyl 2-bromoalkanoates in the presence of potassium bicarbonate in dimethylformamide (Kwiecień, 2004). The Schiff bases **3a**–**l** were prepared by the reaction of alkyl 2-(2-formylphenoxy)alkanoates **1a**–**g** with aniline (**2a**) and 4-methoxyaniline (**2b**) (Scheme 1). To determine the optimal conditions of the process, a series of reactions of methyl 2-(2-formyl-4-nitrophenoxy)butanoate (**1a**) with aniline (**2a**) were carried out using different solvents, such as tetrahydrofuran, 1,2-dichloroethane, and methanol or without any solvent. The influence of the ratio of reagents, reaction time, and catalyst on the yield was also assessed, as indicated in Table 1.

**Table 1:** Synthesis of methyl 2-(4-nitro-2-((phenylimino)methyl)phenoxy)butanoate (**3a**)

Entry	**1a/2a** ratio (mmol/mmol)	Solvent (mL)		Reaction time (h)	Yield^a^ (%)
1	1/1	THF	12	24	91
2	1/1	THF/MeOH	6/6	5.5	93
3	1/1	DCE	8	24	56
4	1/1	DCE^b^	8	24	91
5	1/1.5	DCE^b^	8	24	92
6	1.5/1.5	DCE/MeOH	4/4	72	62
7	1/1	MeOH^b^	6	3.5	99
8	1/1	–	–	24	77^c^

^a^Yield based on GC analysis of the reaction mixture

^b^Catalytic amount of acetic acid was added

^c^Reaction was carried out without any solvent

Based on these studies, we found that a nearly quantitative yield of the desired product was obtained when the reaction was carried out in methanol and in the presence of catalytic amount of the acetic acid, using equimolar ratio of the reactants (Entry 7, Table 1).

Under these established conditions, the reaction proceeded chemoselectively in the formyl group and leaving unchanged the ester group. Importantly, it was observed that the product **3a** could be obtained in high yield by using an aprotic solvent, such as 1,2-dichloroethane in the presence of catalytic amounts of acetic acid. This is important for the direct reductive amination, which is more effective when it is carried out in 1,2-dichloroethane, as demonstrated later. The Schiff base was separated from the reaction mixture by dilution with water, followed by filtration of the precipitate.

Next, the Schiff bases **3b**–**l** were readily prepared by reactions of formyl esters **1b**–**g** with aniline (**2a**) and 4-methoxyaniline (**2b**), in the established reaction conditions (1:1 molar ratio of the formyl ester to amine, methanol as solvent, catalytic amount of acetic acid, room temperature, 3.5 h). The crude products (**3b**–**l**) were crystallized from methanol to yield stable crystals with high melting points (mp); yield: 72–95 %. Some of the Schiff bases exhibit a wide range of their melting points (see experimental section) that is probably caused by presence of (*E*-) and (*Z*-) diastereoisomers in the solid state of the products.

The structures of novel Schiff bases **3a**–**l** were confirmed by gas chromatography mass spectrometry (GCMS), Fourier transform infrared spectroscopy (FTIR), and ^1^H and ^13^C nuclear magnetic resonance (NMR). The infrared spectra exhibited an intense absorption band in the range of 1623–1614 cm^−1^, characteristic of the azomethine groups. Additionally, intense bands, originating from the valence vibrations of the ester carbonyl group, were observed in the range 1756–1733 cm^−1^ (C=O stretch) and 1208–1198 cm^−1^ (C–O stretch). Further, we observed a singlet of integration intensity equivalent to one hydrogen at 9.10–8.94 ppm in the ^1^H NMR spectra of the Schiff bases, indicating the presence of the azomethine proton (–CH=N–).

**Scheme 2:**
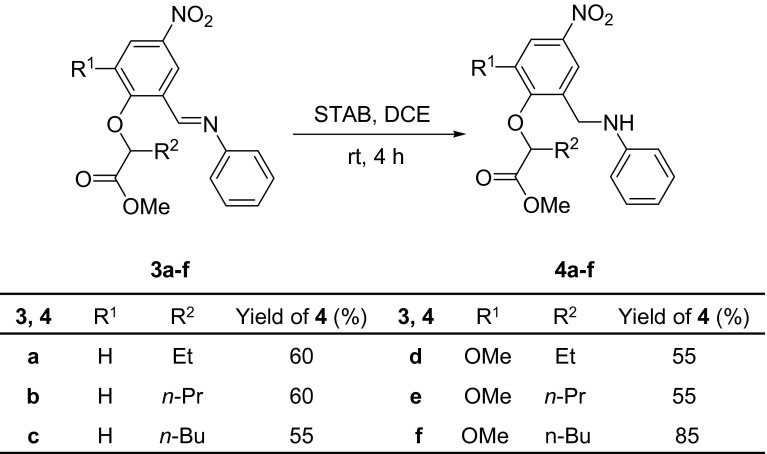
Reduction of Schiff bases **3a**–**f** with STAB into amines **4a**–**f**

**Scheme 3:**
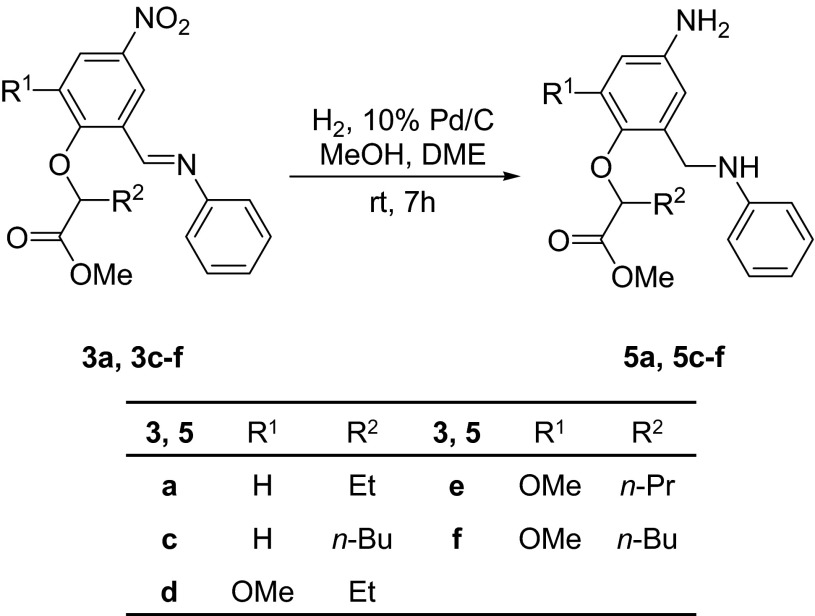
Catalytic reduction of Schiff bases **5a** and **5c**–**f**

Two methods were used for the reduction of Schiff bases. The first one was carried out using sodium triacetoxyborohydride (STAB) as a selective reducing agent of the imino group only and giving nitro amines **4a**–**f** (Scheme 2). The second method consisted of catalytic reduction of both imino and nitro groups, resulting in the formation of diamine compounds **5a**, **c**–**f** (Scheme 3).

To determine the optimal conditions for the reduction of Schiff bases with sodium triacetoxyborohydride to the nitro amines **4a**–**f**, a series of reactions was carried out, starting from model compound **3a** and using the following solvents: methanol, 1,2-dichloroethane, tetrahydrofuran, and *N*,*N*-dimethylformamide (Table 2). Utilizing methanol resulted in a low yield of amino ester, and neither extending the reaction time nor increasing the temperature improved the yield. Similarly, low yield was obtained using *N*,*N*-dimethylformamide in the presence of catalytic amounts of acetic acid. The best result was obtained using a 1:1.5 molar ratio of Schiff base/STAB in 1,2-dichloroethane and a catalytic amount of acetic acid. The reaction was carried out at room temperature for 4 h. Next, the reaction mixture was neutralized with 5 % aqueous solution of sodium bicarbonate, and the organic layer was separated and dried using magnesium sulfate. The product was isolated by solvent removal under reduced pressure and was recrystallized from methanol, to yield pure amino ester **4a**. Under these optimal conditions, the synthesis of compounds **4b**-**f** was readily achieved with moderate-to-good yields (Scheme 2). All of the synthesized amino esters **4a**–**f** are novel compounds. Their structures were established by spectroscopic methods: GC–MS, FTIR, ^1^H, and ^13^C NMR. The FTIR spectra exhibited an intense absorption band in the range of 3406–3385 cm^−1^, characteristic of the amine groups. Additionally, intense bands, originating from the valence vibrations of the ester carbonyl group, were observed in the range 1751–1742 cm^−1^ (C=O stretch) and 1209–1200 cm^−1^ (C–O stretch). In the ^1^H NMR spectra of the amino esters, the multiplet at 4.70–4.31 ppm is consist of two doublets and broad signal which are derived from two protons of CH_2_ and a one proton of NH group, respectively. The deuterium exchange experiment with D_2_O was performed to confirm the presence of amine proton (see Supporting Information).

**Table 2:** Reduction of Schiff base **3a** to amine **4a** with STAB

Entry	**4a**/STAB ratio (mmol/mmol)	Solvent (mL)		Reaction temp.	Reaction time (h)	Yield^a^ (%)
1	1/1.4	DMF^c^	14	100 °C	5	21
2	1/1.4	MeOH	44	rt	3	23
3	1/1.4	MeOH	44	reflux	4	27
4	1/1.4	THF	20	reflux	4	65
5	1/1.7	DCE	20	rt	6	86
6	1/1.5	DCE^b^	17	rt	4	93
7	1/2.0	DCE^b^	17	rt	4	81
8	1/1.5	DCE^c^	17	rt	4	84

^a^Yield based on GC analysis of reaction mixture

^b^Added AcOH (0.1 mL)

^c^Added AcOH (0.2 mL)

Reduction of both nitro and azomethine groups of **3a** and **3c**–**f** was carried out in mild conditions: in methanol with the addition of dimethoxyethane (DME), using a 1:0.1 weight ratio of Schiff base to catalyst, 10 % Pd/C (Scheme 3). The reaction was completed after 7 h, and the products **5a** and **5c**–**f** were obtained after removing of the solvent under vacuum. Diamines **5a**, **c**–**f** were obtained as brown semi-solids, in good yield (71–86 %, Table 3).

**Table 3:** Catalytic reduction of Schiff bases **3a** and **3c**–**f** with hydrogen

**3**	**3**/(10 % Pd/C) ratio (g/g)	MeOH (mL)	DME (mL)	**5**	Yield (%)
**a**	0.31/0.031	40	10	**a**	85
**c**	0.20/0.020	35	7	**c**	82
**d**	0.20/0.020	35	20	**d**	71
**e**	0.36/0.036	40	10	**e**	84
**f**	0.16/0.016	35	7	**f**	74

The FTIR spectra of **5a**, **c**–**f** exhibit an intensive absorption band in the range of 3410–3374 cm^−1^, characteristic of the amine groups. Additionally, intensive bands, originating from the valence vibrations of the ester carbonyl group, were observed in the range 1735–1737 cm^−1^. Finally, the signal at 9.10–8.94 ppm in the ^1^H NMR spectra, associated with azomethine group, disappeared and instead signals at ranges 3.90–3.53 and 1.51–0.78 ppm were observed, indicating the presence of the amine protons.

Subsequently, reductive amination of methyl 2-(2-formylphenoxy)alkanoate **1a**–**f** was investigated as a one-step process. Synthesis of amines via direct reductive amination is very useful, because it does not require isolating the intermediate Schiff bases. This greatly speeds up the process of synthesis and limits losses associated with isolation of the intermediates.

To determine optimal conditions for the direct reductive amination, the model formyl ester **1a** was reacted with aniline (**2a**) in the presence of sodium triacetoxyborohydride as the reducing agent. The reactions were conducted at ambient temperature, using different solvents, and changing the molar ratio of the reactants and reaction times (Table 4).

**Table 4:** Synthesis of **4a** via the direct reductive amination of methyl 2-(2-formyl-4-nitrophenoxy)butanoate (**1a**)

**1a**/**2a**/STAB ratio (mmol/mmol/mmol)	Solvent (mL)	Catalyst	Reaction time (h)	Yield^a^ (%)
1/1/1	THF	–	50	42
1/1/1.45	THF	–	50	61
1/1/1	DCE	–	50	91
1/1.5/1.5	DCE	–	50	98
1/1/1.5	DCE	–	4	59
1/1/1.5	DCE	–	24	86
1/1/1.5	DCE	Amberlyst-15	4	89
1/1/1.5	DCE	AcOH	4	98

^a^Yield based on GC analysis

The highest yield of the desired product was achieved when the reaction was performed in 1,2-dichloroethane with a catalytic amount of acetic acid for 4 h, with an equimolar ratio of formyl ester and aniline and 1.5 mol excess of the catalyst.

**Scheme 4:**
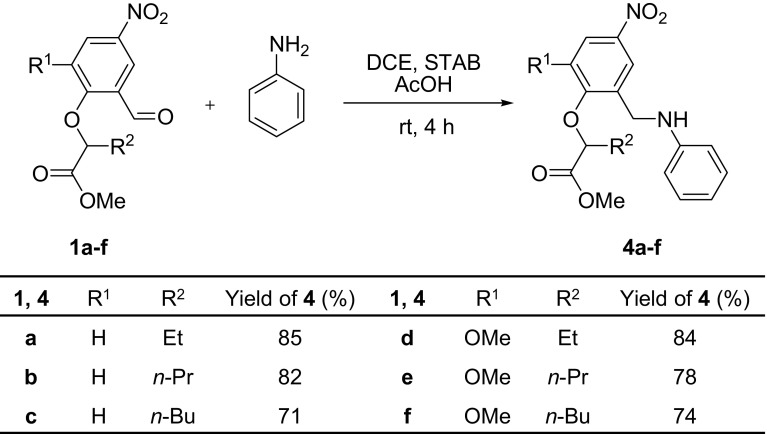
Direct reductive amination of formyl esters **1a**–**f** to **4a**–**f**

The direct reductive amination of **1b**–**f** was carried out using the same conditions as for **1a**, resulting in good yields (71–85 %) of amines **4b**–**f** (Scheme 4). Finally, amino esters **4a**–**f** were converted into their hydrochloride salts.

### Microbiology

All of the synthesized compounds were screened for antibacterial activity against selected clinically important Gram-positive (*S. aureus*, *M. luteus*, *S. mutans*, *E. faecalis*) and Gram-negative (*E. coli*, *P. aeruginosa*, *A. baumannii*) bacteria by the disk diffusion method. Acetone was used as solvent for Schiff bases, and DMSO as solvent for amines and hydrochloride salts of amines. The antibiotic ciprofloxacin (5 mg/mL) was used as a positive control. The results of antibacterial screening indicate that four of eleven tested Schiff bases **3a**, **3c**–**e** exhibit varied activity against Gram-positive bacteria, including *S. aureus*, and *S. mutans*. The bacterial inhibition zone values of the Schiff bases are summarized in Table 5.

**Table 5:** Antibacterial activity^a^ of the Schiff bases **3a** and **3c**–**e** at 10 mg/mL concentration

Compound	*S. aureus*			*S. mutans*
	MSSA ATCC 25923	MRSA ATCC 43300	MLSB clinical isolate	clinical isolate
**3a**	13	8	9	10
**3c**	7	7	8	7
**3d**	10	15	12	–
**3e**	8	13	13	7
ciprofloxacin	25	26	27	28

^a^Zone of inhibition in mm; ‘–’ means not sensitive (no antimicrobial activity was recorded)

Schiff base **3d** exhibited antibacterial activity only against *S. aureus* strains and caused the strongest inhibition of the growth of methicillin-resistant *S. aureus.* Schiff base **3a**, on the other hand, caused the strongest inhibition of the growth of *S. mutans*. It should also be mentioned that the synthesized Schiff bases did not inhibit the growth of *E. faecalis* and *M. luteus*. Further, introducing the methoxyl group into Schiff base structure has a significant impact on antibacterial activity. No antibacterial activity was observed for the Schiff bases containing methoxyl in *N*-substituted phenyl ring (**3h**–**l**, R=OMe). Likewise, the presence of methoxyl group at 2-position in **3d** structure abolishes activity against *S. mutans*, in contrast to *S. aureus*, where activity increases against MRSA. Increasing alkyl, hydrophobic chain length results in decreased antibacterial activity, except for the influence of **3e** on *S. mutans.*

Followed the screening, the minimum inhibitory concentration (MIC) was determined for the Schiff bases that demonstrated activity against specific species of bacteria. The results are presented in Table 6.

**Table 6:** Minimum inhibitory concentration (MIC, mg/mL) of Schiff bases **3a**, **c**–**e**

Compound	*S. aureus*			*S. mutans*
	MSSA ATCC 25923	MRSA ATCC 43300	MLSB clinical isolate	clinical isolate
**3a**	0.10	0.10	0.10	0.50
**3c**	0.50	0.25	0.25	0.50
**3d**	0.25	0.25	0.25	1.00
**3e**	0.25	0.10	0.10	1.00

Next, it was observed that amino esters **4a**-**f** did not show any antimicrobial activity, while their hydrochlorides showed good inhibition of the Gram-positive bacteria (Table 7). The most active were those without the methoxyl groups, but no influence of hydrophobic side chain on the antibacterial activity was observed. The lack of inhibition for amino esters might be caused by the presence of an intramolecular hydrogen bond that can be formed between amino proton and carbonyl group in the amino esters.

**Table 7:** Antibacterial activity^a^ of amino esters **4a**–**f** at 10 mg/mL concentration

Compound	*E. faecalis*	*S. aureus*			*M. luteus*	*S. mutans*
	ATCC 29212	MSSA ATCC 25923	MRSA ATCC 43300	MLSB clinical isolate	PCM 1944	clinical isolate
**4a·**HCl	12	8	–	8	15	11
**4b·**HCl	12	12	7	7	14	14
**4c·**HCl	17	16	13	8	17	21
**4e·**HCl	8	–	–	–	8	–
**4f·**HCl	13	13	14	12	15	15
ciprofloxacin	20	25	26	27	22	28

^a^Zone of inhibition in mm; ‘–’ no antimicrobial activity was recorded

The broadest spectrum of antibacterial activity was noted for **4c**·HCl. This compound inhibited the growth of all Gram-positive bacteria and was the only compound synthesized in these studies that inhibited the growth of the Gram-negative *A. baumannii*. The MIC of hydrochloride salts of **4a**–**f** is given in Table 8.

**Table 8:** Minimum inhibitory concentration (MIC, mg/mL) of hydrochloride salts of the amino esters **4a**–**f**

	Gram-positive					
	*E. faecalis*	*S. aureus*			*M. luteus*	*S. mutans*
	ATCC 29212	MSSA ATCC 25923	MRSA ATCC 43300	MLSB clinical isolate	PCM 1944	clinical isolate
**4a·**HCl	0.50	0.50	NT	1.00	0.50	0.50
**4b·**HCl	0.25	0.10	0.50	1.00	0.05	0.25
**4c·**HCl	0.05	0.10	0.25	0.50	0.05	0.10
**4e·**HCl	0.50	NT	NT	NT	1.00	NT
**4f·**HCl	0.05	0.01	0.25	0.25	0.05	0.10

*NT* means not tested

Three other hydrochloride salts of amino esters, **4a**·HCl, **4b**·HCl, **4f**·HCl, inhibited the growth of all Gram-positive bacteria, having MICs of 0.50–1.00, 0.05–1.0, and 0.01–0.25 mg/mL, respectively, for each bacterial strain. Moderate antibacterial activity was also recorded for **4e**·HCl, which inhibited the growth of *E. faecalis* and *M. luteus*. The most encouraging results against Gram-positive bacteria were obtained for compounds **4c**·HCl and **4f**·HCl having MICs of 0.05–0.50 and 0.01–0.25 mg/mL, respectively.

The structure–activity relationships of the tested compounds can be summarized as follows: (1) in series of the Schiff bases, the presence of methoxyl group at the *N*-phenyl ring has a significant negative impact on their antibacterial activity; (2) in the same series, elongation of hydrophobic, alkyl chain causes the decrease in antibacterial activity against *S. aureus* MSSA and MRSA; (3) reduction of azomethine group to amine causes loss of activity against all of the tested microorganism; (4) in series of amine hydrochlorides, the presence of methoxyl group at the *N*-phenyl ring has the same negative impact on their antibacterial activity as in the case of Schiff bases; opposite effect is observed for elongation of hydrophobic, alkyl chain that causes the increase in antibacterial activity.

Lack of activity of the tested substances against Gram-negative bacteria could be explained by the differences in the structure of the cell walls of Gram-positive and Gram-negative microorganisms. In most Gram-positive bacteria, the cell wall consists of many layers of peptidoglycan, forming a thick, rigid structure. The cell walls of Gram-negative bacteria consist of one or a very few layers of peptidoglycan and a lipid-rich outer membrane (Beveridge, 1999). However, the mechanism responsible for the antibacterial activity of examined compounds is not known at the moment; work is in progress to clarify in detail the mechanism of antibacterial action, as well as the design of more effective compounds.

## Conclusion

We demonstrate simple and efficient methods for both stepwise and direct reductive amination of 2-(2-formyl-4-nitrophenoxy)alkanoic acid derivatives, yielding secondary *N*-arylated amines via Schiff bases under mild conditions. The reduction step was performed utilizing sodium triacetoxyborohydride, as well as catalytic hydrogenation using palladium(0) catalyst. Our antibacterial screening assay indicates that some of Schiff bases and secondary amine hydrochlorides possess moderate-to-good activity against Gram-positive bacteria, including *S. aureus*, *M. luteus*, and *S. mutans*. In a series of Schiff bases, we observe some of the influence of chain length and presence of methoxyl group on the antibacterial activity. Further modification of the selected compounds based on the information obtained from these results, as well as molecular modeling and structure–activity relationship studies, are in progress.

## Materials and methods

### Chemistry

^1^H and ^13^C NMR spectra were recorded on a TM Bruker DPX 400 (400 MHz) instrument, with CDCl_3_ solvent. Chemical shifts *δ* are given from TMS (0 ppm), as an internal standard for ^1^H NMR, and CDCl_3_ (77.0 ppm), for ^13^C NMR (100 MHz). Mass spectra were obtained using an Agilent Technologies 6890 N apparatus, equipped with a mass detector 5973 Network and 30 m × 0.25 mm capillary column, filled with a 0.25-μm film of 5 % MePh silicate. Fourier transform infrared-attenuated total reflection spectroscopy (FTIR–ATR) was performed using a Nexus spectrometer with Golden Gate (ATR) (Thermo Nicolet Corp.). Samples were dried at 60 °C, under vacuum for 24 h, and 32 scans were averaged across the spectral range of 400–4000 cm^−1^. All melting points were determined using a Boetius apparatus and are uncorrected.

**Fig. 1:**
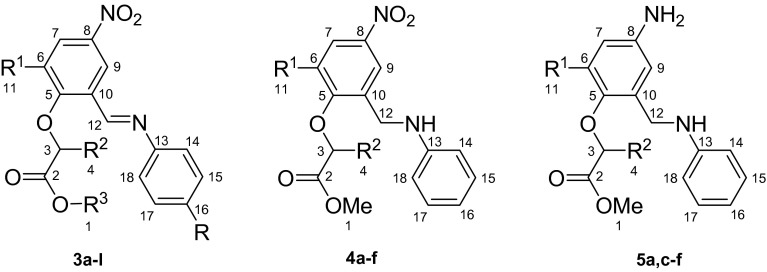
Numbering for ^13^C NMR spectra

Most of the reagents and solvents were purchased in commercially available grade purity. 2-Hydroxy-3-methoxy-5-nitrobenzaldehyde (mp 140–142 °C) and 2-hydroxy-5-nitrobenzaldehyde (mp 129–131 °C) were obtained by nitration of appropriate 2-hydroxy-3-methoxybenzaldehyde or 2-hydroxybenzaldehyde with 100 % nitric acid in acetic acid solution (Kwiecień and Szychowska, 2007), although they are commercially available. Methyl 2-(2-formyl-4-nitrophenoxy)alkanoates **1a**–**c** were prepared starting from appropriate 2-bromoesters and 2-hydroxy-5-nitrobenzaldehyde according to the literature (Kwiecień, 2004). Methyl 2-(2-formyl-6-methoxy-4-nitrophenoxy)alkanoates **1d**–**f** and ethyl 2-formyl-6-methoxy-4-nitrophenoxy)acetate **1g** were obtained starting from 2-hydroxy-3-methoxy-5-nitrobenzaldehyde and appropriate 2-bromoesters according to procedures in Kwiecień (2004) and Kwiecień and Szychowska (2007), respectively (Fig. 1).

### General procedure for the synthesis of methyl 2-(4-nitro-2-((phenylimino)methyl)phenoxy)alkanoate (3a–l)

A mixture of alkyl 2-(2-formyl-4-nitrophenoxy)alkanoate (**1a**–**g**) (1.87 mmol), aniline (**2a**) or *p*-methoxyaniline (**2b**) (1.87 mmol), methanol (50 mL), and acetic acid (0.2 mL) was stirred magnetically at room temperature for 3.5 h. Then, the reaction mixture was poured into water (27 mL) and allowed to stand at 5–7 °C (in a refrigerator) for 24 h. The precipitate was filtered off, washed with water, and recrystallized from hot methanol to give **3a**–**l**.

#### *Methyl 2-(4-nitro-2-((phenylimino)methyl)phenoxy)butanoate (****3a****)*

Colorless solid (MeOH) This compound (**3a**) was prepared from methyl 2-(2-formyl-4-nitrophenoxy)butanoate (**1a**) (1.87 mmol, 0.50 g) and aniline (**2a**) (1.87 mmol, 0.17 g) according to the general procedure. The product obtained as a colorless solid was purified from methanol. 0.61 g (95 %); mp 111–117 °C; FTIR (neat) *ν*_max_: 3081, 2975, 1744, 1620, 1585, 1510, 1340, 1210, 1077 cm^−1^; ^1^H NMR (CDCl_3_, 400 MHz): *δ* = 9.09 (d, *J* = 2.9 Hz, 1H, Ar), 8.95 (s, 1H, N=CH), 8.27 (dd, *J* = 2.9, 9.1 Hz, 1H, Ar), 7.49–7.38 (m, 2H, Ar), 7.30–7.24 (m, 3H, Ar), 6.86 (d, *J* = 9.1 Hz, 1H, Ar), 4.83 (t, *J* = 6.0 Hz, 1H, CH), 3.78 (s, 3H, OCH_3_), 2.18–2.05 (m, 2H, CH_2_), 1.12 (t, *J* = 7.4 Hz, 3H, CH_3_); ^13^C NMR (CDCl_3_, 101 MHz): *δ* = 170.5 (C, C-2), 161.7 (CH, C-12), 153.4 (C, C-5), 151.6 (C, C-13), 142.4 (C, C-8), 129.3 (C, C-15, C-17), 127.4 (C, C-7), 126.6 (C, C-9), 126.2 (C, C-10), 124.0 (C, C-16), 121.1 (C, C-14, C-18), 112.5 (C, C-6), 78.4 (CH, C-3), 52.6 (OCH_3_, C-1), 26.0 (CH_2_CH_3_, C-4), 9.7 (CH_2_CH_3_, C-4); GCMS *m/z* 342 [M]^+^ (32), 327 (9), 283 (32), 250 (18), 225 (18), 222 (5), 195 (23), 190 (8), 179 (7), 175 (100), 145 (23), 139 (7), 104 (17), 93 (22), 77 (48), 59 (29), 51 (8); Anal. Calcd for C_18_H_18_N_2_O_5_: C, 63.15; H, 5.30; N, 8.18. Found: 63.12; H, 5.40; N, 8.15.

#### *Methyl 2-(4-nitro-2-((phenylimino)methyl)phenoxy)pentanoate (****3b****)*

Colorless solid (MeOH) This compound (**3b**) was prepared from methyl 2-(2-formyl-4-nitrophenoxy)pentanoate (**1b**) (1.78 mmol, 0.50 g) and aniline (**2a**) (1.78 mmol, 0.162 g) according to the general procedure. The product obtained as a colorless solid was purified from methanol. 0.48 g (76 %); mp 84–87 °C; FTIR (neat) *ν*_max_: 3102, 2962, 1733, 1614, 1579, 1515, 1341, 1268, 1078 cm^−1^; ^1^H NMR (CDCl_3_, 400 MHz): *δ* = 9.10 (d, *J* = 2.9 Hz, 1H, Ar), 8.94 (s, 1H, N=CH), 8.28 (dd, *J* = 2.9, 9.1 Hz, 1H, Ar), 7.47–7.41 (m, 2H, Ar), 7.32–7.25 (m, 3H, Ar), 6.87 (d, *J* = 9.1 Hz, 1H, Ar), 4.88 (dd, *J* = 4.9, 7.6 Hz, 1 H, CH), 3.79 (s, 3H, OCH_3_), 2.14–1.98 (m, 2H, CH_2_), 1.65–1.50 (m, 2H, CH_2_), 1.01 (t, *J* = 7.4 Hz, 3H, CH_3_); ^13^C NMR (CDCl_3_, 101 MHz): *δ* = 170.7 (C, C-2), 161.7 (CH, C-12), 153.5 (C, C-5), 151.6 (C, C-13), 142.3 (C, C-8), 129.2 (C, C-15, C-17), 127.4 (C, C-7), 126.6 (C, C-9), 126.1 (C, C-10), 124.0 (C, C-16), 121.1 (C, C-14, C-18), 112.5 (C, C-6), 77.2 (CH, C-3), 52.6 (OCH_3_, C-1), 34.5 (CH_2_CH_2_CH_3_, C-4), 18.6 (CH_2_CH_2_CH_3_, C-4), 13.7 (CH_2_CH_2_CH_3_, C-4); GCMS *m/z* 356 [M]^+^ (55), 327 (55), 297 (32), 264 (23), 241 (16), 225 (20), 204 (9), 195 (29), 189 (100), 179 (7), 167 (19), 145 (12), 130 (5), 118 (5), 104 (16), 93 (29), 77 (42), 59 (19); Anal. Calcd for C_19_H_20_N_2_O_5_: C, 64.04; H, 5.66; N, 7.86. Found: C, 64.14; H, 5.68; N, 7.82.

#### *Methyl 2-(4-nitro-2-((phenylimino)methyl)phenoxy)hexanoate (****3c****)*

Colorless solid (MeOH) This compound (**3c**) was prepared from methyl 2-(2-formyl-4-nitrophenoxy)hexanoate (**1c**) (1.69 mmol, 0.50 g) and aniline (**2a**) (1.69 mmol, 0.154 g) according to the general procedure. The product obtained as a colorless solid was purified from methanol. 0.56 g (89 %); mp 68–71 °C; FTIR (neat) *ν*_max_: 3088, 2956, 1737, 1611, 1579, 1510, 1339, 1203, 1072 cm^−1^; ^1^H NMR (CDCl_3_, 400 MHz): *δ* = 9.09 (d, *J* = 2.9 Hz, 1H, Ar), 8.94 (s, 1H, N=CH), 8.27 (dd, *J* = 2.9, 9.1 Hz, 1H, Ar), 7.46–7.40 (m, 2H, Ar), 7.31–7.25 (m, 3H, Ar), 6.86 (d, *J* = 9.1 Hz, 1H, Ar), 4.86 (t, *J* = 6.2 Hz, 1H, CH), 3.78 (s, 3H, OCH_3_), 2.15–2.00 (m, 2H, CH_2_), 1.57–1.46 (m, 2H, CH_2_), 1.46–1.35 (m, 2H, CH_2_), 0.94 (t, *J* = 7.2 Hz, 3H, CH_3_); ^13^C NMR (CDCl_3_, 101 MHz): *δ* = 170.7 (C, C-2), 161.7 (CH, C-12), 153.5 (C, C-5), 151.6 (C, C-13), 142.4 (C, C-8), 129.3 (C, C-15, C-17), 127.4 (C, C-7), 126.6 (C, C-9), 126.1 (C, C-10), 124.0 (C, C-16), 121.1 (C, C-14, C-18), 112.5 (C, C-6), 77.4 (CH, C-3), 52.7 (OCH_3_, C-1), 32.2 (CH_2_CH_2_CH_2_CH_3_, C-4), 27.3 (CH_2_CH_2_CH_2_CH_3_, C-4), 22.2 (CH_2_CH_2_CH_2_CH_3_, C-4), 13.9 (CH_2_CH_2_CH_2_CH_3_, C-4); GCMS *m/z* 370 [M]^+^ (51), 327 (52), 311 (31), 295 (6), 278 (24), 242 (27), 225 (21), 203 (100), 195 (32), 188 (10), 167 (21), 145 (10), 104 (17), 93 (40), 77 (46), 59 (20); Anal. Calcd for C_20_H_22_N_2_O_5_: C, 64.85; H, 5.99; N, 7.56. Found: C, 64.80; H, 5.64; N, 7.83.

#### *Methyl 2-(2-methoxy-4-nitro-6-((phenylimino)methyl)phenoxy)butanoate (****3d****)*

Colorless solid (MeOH) This compound (**3d**) was prepared from methyl 2-(2-formyl-6-methoxy-4-nitrophenoxy)butanoate (**1d**) (1.68 mmol, 0.50 g) and aniline (**2a**) (1.68 mmol, 0.153 g) according to the general procedure. The product obtained as a colorless solid was purified from methanol. 0.53 g (85 %); mp 124-129 °C; FTIR (neat) *ν*_max_: 3088, 2977, 1746, 1619, 1577, 1518, 1341, 1202, 1085 cm^−1^; ^1^H NMR (CDCl_3_, 400 MHz): *δ* = 9.09 (s, 1H, N=CH), 8.74 (d, *J* = 2.9 Hz, 1H, Ar), 7.83 (d, *J* = 2.9 Hz, 1H, Ar), 7.46–7.39 (m, 2H, Ar), 7.33–7.24 (m, 3H, Ar), 5.13 (dd, *J* = 5.4, 6.9, 1H, CH), 3.96 (s, 3H, OCH_3_), 3.69 (s, 3H, OCH_3_), 2.13–1.97 (m, 2H, CH_2_), 1.11 (t, *J* = 7.6 Hz, 3H, CH_3_); ^13^C NMR (CDCl_3_, 101 MHz): *δ* = 171.4 (C, C-2), 155.0 (C, C-12), 152.0 (C, C-5), 151.7 (C, C-6), 151.6 (C, C-13), 143.7 (C, C-8), 130.2 (C, C-10), 129.2 (C, C-15, C-17), 126.5 (C, C-16), 121.2 (C, C-14, C-18), 115.6 (C, C-9), 108.9 (C, C-7), 81.4 (CH, C-3), 56.4 (OCH_3_, C-11), 52.1 (OCH_3_, C-1), 26.5 (CH_2_CH_3_, C-4), 9.4 (CH_2_CH_3_, C-4); GCMS *m/z* 372 [M]^+^ (61), 357 (11), 313 (34), 280 (37), 280 (37), 271 (15), 255 (22), 252 (7), 236 (12), 225 (22), 210 (8), 205 (100), 196 (12), 190 (29), 175 (45), 154 (25), 127 (6), 104 (16), 93 (19), 77 (38), 59 (23), 51 (6); Anal. Calcd for C_19_H_20_N_2_O_6_: C, 61.28; H, 5.41; N, 7.52. Found: C, 61.24; H, 5.39; N, 7.55.

#### *Methyl 2-(2-methoxy-4-nitro-6-((phenylimino)methyl)phenoxy)pentanoate (****3e****)*

Colorless solid (MeOH) This compound (**3e**) was prepared from methyl 2-(2-formyl-6-methoxy-4-nitrophenoxy)pentanoate (**1e**) (1.61 mmol, 0.50 g) and aniline (**2a**) (1.62 mmol, 0.147 g) according to the general procedure. The product obtained as a colorless solid was purified from methanol. 0.53 g (85 %); mp 126–128 °C; FTIR (neat) *ν*_max_: 3086, 2961, 1745, 1623, 1575, 1517, 1328, 1205, 1071 cm^−1^; ^1^H NMR (CDCl_3_, 400 MHz): *δ* = 9.08 (s, 1H, N=CH), 8.74 (d, *J* = 2.7 Hz, 1H, Ar), 7.83 (d, *J* = 2.7 Hz, 1H, Ar), 7.45–7.39 (m, 2H, Ar), 7.45–7.39 (m, 3H, Ar), 5.19 (dd, *J* = 5.6, 6.7 Hz, 1H, CH), 3.96 (s, 3H, OCH_3_), 3.67 (s, 3H, OCH_3_), 2.06–1.91 (m, 2H, CH_2_), 1.65–1.51 (m, 2H, CH_2_), 1.00 (t, *J* = 7.4 Hz, 3H, CH_3_); ^13^C NMR (CDCl_3_, 101 MHz): *δ* = 171.6 (C, C-2), 155.1 (C, C-12), 152.0 (C, C-5), 151.7 (C, C-6), 151.6 (C, C-13), 143.7 (C, C-8), 130.2 (C, C-10), 129.2 (C, C-15, C-17), 126.5 (C, C-16), 121.2 (C, C-14, C-18), 115.6 (C, C-9), 108.8 (C, C-7), 80.1 (CH, C-3), 56.4 (OCH_3_, C-11), 52.1 (OCH_3_, C-1), 35.2 (CH_2_CH_2_CH_3_, C-4), 18.3 (CH_2_CH_2_CH_3_, C-4), 13.7 (CH_2_CH_2_CH_3_, C-4); GCMS *m/z* 386 [M]^+^ (52), 357 (34), 327 (23), 297 (8), 294 (36), 271 (16), 255 (23), 235 (13), 219 (100), 204 (25), 189 (36), 175 (15), 154 (27), 127 (7), 104 (18), 93 (32), 77 (42), 59 (20); Anal. Calcd for C_20_H_22_N_2_O_6_: C, 62.17; H, 5.74; N, 7.25. Found: C, 62.47; H, 5.70; N, 7.30.

#### *Methyl 2-(2-methoxy-4-nitro-6-((phenylimino)methyl)phenoxy)hexanoate (****3f****)*

Colorless solid (MeOH) This compound (**3f**) was prepared from methyl 2-(2-formyl-6-methoxy-4-nitrophenoxy)hexanoate (**1f**) (1.54 mmol, 0.50 g) and aniline (**2a**) (1.54 mmol, 0.14 g) according to the general procedure. The product obtained as a colorless solid was purified from methanol. 0.48 g (78 %); mp 82–91 °C; FTIR (neat) *ν*_max_: 3090, 2953, 1744, 1619, 1575, 1525, 1322, 1200, 1091 cm^−1^; ^1^H NMR (CDCl_3_, 400 MHz): *δ* = 9.08 (s, 1H, N=CH), 8.74 (d, *J* = 2.7 Hz, 1H, Ar), 7.83 (d, *J* = 2.7 Hz, 1H, Ar), 7.46–7.39 (m, 2H, Ar), 7.33–7.24 (m, 3H, Ar), 5.17 (t, *J* = 6.1 Hz, 1H, CH), 3.96 (s, 3H, OCH_3_), 3.68 (s, 3H, OCH_3_), 2.06–1.96 (m, 2H, CH_2_), 1.58–1.46 (m, 2H, CH_2_), 1.45–1.34 (m, 2H, CH_2_), 0.93 (t, *J* = 7.3 Hz, 3H, CH_3_); ^13^C NMR (CDCl_3_, 101 MHz): *δ* = 171.6 (C, C-2), 155.2 (C, C-12), 152.0 (C, C-5), 151.7 (C, C-6), 151.6 (C, C-13), 143.7 (C, C-8), 130.2 (C, C-10), 129.2 (C, C-15, C-17), 126.5 (C, C-16), 121.2 (C, C-14, C-18), 115.5 (C, C-9), 108.8 (C, C-7), 80.3 (CH, C-3), 56.4 (OCH_3_, C-11), 52.1 (OCH_3_, C-1), 32.9 (CH_2_CH_2_CH_2_CH_3_, C-4), 27.1 (CH_2_CH_2_CH_2_CH_3_, C-4), 22.4 (CH_2_CH_2_CH_2_CH_3_, C-4), 13.9 (CH_2_CH_2_CH_2_CH_3_, C-4); GCMS *m/z* 400 [M]^+^ (67), 369 (5), 357 (43), 341 (29), 308 (43), 297 (7), 276 (6), 271 (19), 255 (25), 248 (10), 233 (100), 226 (9), 210 (8), 203 (36), 196 (14), 180 (14), 154 (27), 127 (6), 104 (16), 93 (33), 77 (36), 69 (14); Anal. Calcd for C_21_H_24_N_2_O_6_: C, 62.99; H, 6.04; N, 7.00. Found: C, 63.10; H, 6.01; N, 6.97.

#### *Ethyl 2-(2-methoxy-4-nitro-6-((phenylimino)methyl)phenoxy)acetate (****3g****)*

Colorless solid (MeOH) This compound (**3** **g**) was prepared from ethyl 2-(2-formyl-4-nitrophenoxy)acetate (**1** **g**) (1.77 mmol, 0.50 g) and aniline (**2a**) (1.77 mmol, 0.161 g) according to the general procedure. The product obtained as an aquamarine solid was purified from methanol. 0.57 g (90 %); mp 98–101 °C; FTIR (neat) *ν*_max_: 3107, 2944, 1756, 1617, 1577, 1516, 1337, 1199 cm^−1^; ^1^H NMR (CDCl_3_, 400 MHz): *δ* = 9.10 (s, 1H, N=CH), 8.74 (d, *J* = 2.6 Hz, 1H, Ar), 7.85 (d, *J* = 2.6 Hz, 1H, Ar), 7.45–7.38 (m, 2H, Ar), 7.34–7.23 (m, 3H, Ar), 4.91 (s, 2H, CH_2_), 4.22 (q, *J* = 7.1 Hz, 2H, OCH_2_), 3.99 (s, 3H, OCH_3_),1.24 (t, *J* = 7.1 Hz, 3H, CH_3_); ^13^C NMR (CDCl_3_, 101 MHz): *δ* = 168.8 (C, C-2), 154.8 (CH, C-12), 152.2 (C, C-5), 151.8 (C, C-6), 151.4 (C, C-13), 144.0 (C, C-8), 130.1 (C, C-10), 129.2 (C, C-15, C-17), 126.6 (C, C-16), 121.3 (C, C-14, C-18), 115.4 (C, C-9), 108.9 (C, C-7), 69.5 (CH_2_, C-3), 61.4 (OCH_2_CH_3_, C-1), 56.5 (OCH_3_, C-11), 14.1 (OCH_2_CH_3_, C-1); GCMS *m/z* 358 [M]^+^ (19), 329 (3), 285 (52), 255 (6), 239 (16), 225 (6), 210 (5), 194 (22), 179 (7), 167 (6), 148 (31), 104 (11), 93 (100), 77 (28), 61 (5). Anal. Calcd for C_19_H_20_N_2_O_6_: C, 61.28; H, 5.41; N, 7.52. Found: C, 61.32; H, 5.52; N, 7.50.

#### *Methyl 2-(2-((4-methoxyphenylimino)methyl)-4-nitrophenoxy)butanoate (****3h****)*

Aquamarine solid (MeOH) This compound (**3** **h**) was prepared from methyl 2-(2-formyl-4-nitrophenoxy)butanoate (**1a**) (1.69 mmol, 0.45 g) and *p*-methoxyaniline (**2b**) (1.69 mmol, 0.205 g) according to the general procedure. The product obtained as an aquamarine solid was purified from methanol. 0.52 g (83 %); mp 108.5–110.5 °C; FTIR (neat) *ν*_max_: 3090, 2932, 1737, 1615, 1596, 1510, 1342, 1214, 1075 cm^−1^; ^1^H NMR (CDCl_3_, 400 MHz): *δ* = 9.08 (d, *J* = 2.9 Hz, 1H, Ar), 8.96 (s, 1H, N=CH), 8.24 (dd, *J* = 2.9, 9.1 Hz, 1H, Ar), 7.33–7.28 (m, 2H, Ar), 6.99–6.94 (m, 2H, Ar), 6.84 (d, *J* = 9.1 Hz, 1H, Ar), 4.82 (t, *J* = 6.0 Hz, 1H, CH), 3.85 (s, 3H, OCH_3_), 3.78 (s, 3H, OCH_3_), 2.12 (p, *J* = 7.4 Hz, 2H, CH_2_), 1.11 (t, *J* = 7.4 Hz, 3H, CH_3_); ^13^C NMR (CDCl_3_, 101 MHz): *δ* = 170.5 (C, C-2), 161.5 (C, C-12), 158.8 (C, C-5), 151.0 (C, C-16), 144.3 (C, C-13), 142.3 (C, C-8), 127.0 (C, C-7), 126.4 (C, C-10), 123.6 (C, C-9), 122.6 (C, C-14, C-18), 114.4 (C, C-15, C-17), 112.5 (C, C-6), 78.3 (CH, C-3), 55.5 (OCH_3_, C-19), 52.6 (OCH_3_, C-1), 26.0 (CH_2_CH_3_, C-4), 9.6 (CH_2_CH_3_, C-4); GCMS *m/z* 372 [M]^+^ (100), 313 (16), 271 (9), 257 (12), 241 (11), 225 (16), 190 (5), 175 (54), 160 (7), 145 (14), 134 (7), 122 (60), 108 (7), 92 (10), 77 (10), 59 (14); Anal. Calcd for C_19_H_20_N_2_O_6_: C, 61.28; H, 5.41; N, 7.52. Found: C, 61.32; H, 5.52; N, 7.50.

#### *Methyl 2-(2-((4-methoxyphenylimino)methyl)-4-nitrophenoxy)hexanoate (****3i****)*

Pale yellow solid (MeOH) This compound (**3i**) was prepared from methyl 2-(2-formyl-4-nitrophenoxy)hexanoate (**1c**) (1.69 mmol, 0.50 g) and *p*-methoxyaniline (**2b**) (1.69 mmol, 0.208 g) according to the general procedure. The product obtained as a pale yellow solid was purified from methanol. 0.49 g (72 %); mp 102–104 °C; FTIR (neat) *ν*_max_: 3090, 2927, 1739, 1616, 1592, 1516, 1333, 1198, 1080 cm^−1^; ^1^H NMR (CDCl_3_, 400 MHz): *δ* = 9.08 (d, *J* = 2.9 Hz, 1H, Ar), 8.95 (s, 1H, N=CH), 8.24 (dd, *J* = 2.9, 9.2 Hz, 1H, Ar), 7.33–7.28 (m, 2H, Ar), 6.99–6.93 (m, 2H, Ar), 6.84 (d, *J* = 9.2 Hz, 1H, Ar), 4.85 (undivided dd, *J* = 5.6, 6.8 Hz, 1H, CH), 3.85 (s, 3H, OCH_3_), 3.78 (s, 3H, OCH_3_), 2.12–2.02 (m, 2H, CH_2_), 1.57–1.47 (m, 2H, CH_2_), 1.47–1.36 (m, 2H, CH_2_), 0.94 (t, *J* = 7.2 Hz, 3H, CH_3_); ^13^C NMR (CDCl_3_, 101 MHz): *δ* = 170.8 (C, C-2), 161.5 (C, C-12), 158.8 (C, C-5), 151.1 (C, C-16), 144.3 (C, C-13), 142.3 (C, C-8), 127.0 (C, C-7), 126.4 (C, C-10), 123.7 (C, C-9), 122.6 (C, C-14, C-18), 114.4 (C, C-15, C-17), 112.4 (C, C-6), 77.4 (CH, C-3), 55.5 (OCH_3_, C-19), 52.6 (OCH_3_, C-1), 32.2 (CH_2_CH_2_CH_2_CH_3_, C-4), 27.3 (CH_2_CH_2_CH_2_CH_3_, C-4), 22.2 (CH_2_CH_2_CH_2_CH_3_, C-4), 13.9 (CH_2_CH_2_CH_2_CH_3_, C-4); GCMS *m/z* 400 [M]^+^ (100), 357 (12), 341 (14), 297 (6), 272 (15), 257 (13), 241 (9), 225 (16), 203 (52), 182 (6), 173 (6), 154 (7), 145 (6), 134 (6), 122 (40), 108 (6), 92 (8), 77 (8), 59 (8); Anal. Calcd for C_21_H_24_N_2_O_6_: C, 62.99; H, 6.04; N, 7.00. Found: C, 62.96; H, 6.14; N, 7.02.

#### *Methyl 2-(2-((4-methoxyphenylimino)methyl)-6-methoxy-4-nitrophenoxy)butanoate (****3j****)*

Beige solid (MeOH) This compound (**3j**) was prepared from methyl 2-(2-formyl-6-methoxy-4-nitrophenoxy)butanoate (**1d**) (3.37 mmol, 1.00 g) and *p*-methoxyaniline (**2b**) (3.37 mmol, 0.415 g) according to the general procedure. The product obtained as a beige solid was purified from methanol. 1.20 g (89 %); mp 116–117 °C; FTIR (neat) *ν*_max_: 3099, 2948, 1746, 1619, 1577, 1518, 1341, 1202, 1091 cm^−1^; ^1^H NMR (CDCl_3_, 400 MHz): *δ* = 9.10 (s, 1H, N=CH), 8.73 (d, *J* = 2.6 Hz, 1H, Ar), 7.80 (d, *J* = 2.6 Hz, 1H, Ar), 7.37–7.32 (m, 2H, Ar), 7.00–6.93 (m, 2H, Ar), 5.10 (dd *J* = 5.6, 6.4 Hz, 1H, CH), 3.95 (s, 3H, OCH_3_), 3.85 (s, 3H, OCH_3_), 3.69 (s, 3H, OCH_3_), 2.14–1.98 (m, 2H, CH_2_), 1.11 (t, *J* = 7.5 Hz, 3H, CH_3_); ^13^C NMR (CDCl_3_, 101 MHz): *δ* = 171.4 (C, C-2), 158.8 (C, C-12), 152.6 (C, C-16), 151.7 (C, C-5, C-6), 144.3 (C, C-13), 143.7 (C, C-8), 130.5 (C, C-10), 122.7 (C, C-14, C-18), 115.4 (C, C-9), 114.4 (C, C-15, C-17), 108.5 (C, C-7), 81.4 (CH, C-3), 56.4 (OCH_3_, C-11), 55.5 (OCH_3_, C-19), 52.0 (OCH_3_, C-1), 26.5 (CH_2_CH_3_, C-4), 9.4 (CH_2_CH_3_, C-4); GCMS *m/z* 402 [M]^+^ (100), 343 (12), 301 (11), 281 (7), 271 (11), 255 (20), 236 (7), 221 (12), 205 (88), 190 (25), 175 (40), 162 (7), 134 (14), 122 (48), 108 (10), 92 (12), 77 (15), 59 (22); Anal. Calcd for C_20_H_22_N_2_O_7_: C, 59.70; H, 5.51; N, 6.96. Found: C, 59.82; H, 5.62; N, 6.76.

#### *Methyl 2-(2-((4-methoxyphenylimino)methyl)-6-methoxy-4-nitrophenoxy)pentanoate (****3k****)*

Pale yellow solid (MeOH) This compound (**3k**) was prepared from methyl 2-(2-formyl-6-methoxy-4-nitrophenoxy)pentanoate (**1e**) (3.22 mmol, 1.00 g) and *p*-methoxyaniline (**2b**) (3.22 mmol, 0.396 g) according to the general procedure. The product obtained as a pale yellow solid was purified from methanol. 1.20 g (90 %); mp 105–106 °C; FTIR (neat) *ν*_max_: 3099, 2952, 1736, 1616, 1572, 1522, 1342, 1204, 1087 cm^−1^; ^1^H NMR (CDCl_3_, 400 MHz): *δ* = 9.10 (s, 1H, N=CH), 8.73 (d, *J* = 2.5 Hz, 1H, Ar), 7.80 (d, *J* = 2.6 Hz, 1H, Ar), 7.40–7.31 (m, 2H, Ar), 7.00–6.92 (m, 2H, Ar), 5.16 (undivided dd, *J* = 5.6, 6.8 Hz, 1H, CH), 3.95 (s, 3H, OCH_3_), 3.85 (s, 3H, OCH_3_), 3.67 (s, 3H, OCH_3_), 2.09–1.90 (m, 2H, CH_2_), 1.71–1.46 (m, 2H, CH_2_), 1.00 (t, *J* = 7.4 Hz, 3H, CH_3_); ^13^C NMR (CDCl_3_, 101 MHz): *δ* = 171.7 (C, C-2), 158.8 (C, C-12), 152.7 (C, C-16), 151.7 (C, C-5, C-6), 144.3 (C, C-13), 143.7 (C, C-8), 130.5 (C, C-10), 122.7 (C, C-14, C-18), 115.3 (C, C-9), 114.4 (C, C-15, C-17), 108.4 (C, C-7), 80.1 (CH, C-3), 56.3 (OCH_3_, C-11), 55.5 (OCH_3_, C-19), 52.1 (OCH_3_, C-1), 35.2 (CH_2_CH_2_CH_3_, C-4), 18.3 (CH_2_CH_2_CH_3_, C-4), 13.8 (CH_2_CH_2_CH_3_, C-4); GCMS *m/z* 416 [M]^+^ (100), 387 (8), 357 (11), 301 (10), 285 (6), 271 (5), 255 (17), 235 (8), 219 (61), 204 (15), 189 (22), 184 (6), 175 (9), 134 (8), 122 (21), 108 (6), 92 (6), 77 (7), 59 (8); Anal. Calcd for C_21_H_24_N_2_O_7_: C, 60.57; H, 5.81; N, 6.73. Found: C, 60.65; H, 5.72; N, 6.73.

#### *Methyl 2-(2-((4-methoxyphenylimino)methyl)-6-methoxy-4-nitrophenoxy)hexanoate (****3l****)*

Aquamarine solid (MeOH) This compound (**3l**) was prepared from methyl 2-(2-formyl-6-methoxy-4-nitrophenoxy)hexanoate (**1f**) (3.08 mmol, 1.00 g) and *p*-methoxyaniline (**2b**) (3.08 mmol, 0.379 g) according to the general procedure. The product obtained as an aquamarine solid was purified from methanol. 1.32 g (76 %); mp 116–119 °C; FTIR (neat) *ν*_max_: 3102, 2955, 1739, 1616, 1592, 1516, 1333, 1198, 1092 cm^−1^; ^1^H NMR (CDCl_3_, 400 MHz): *δ* = 9.10 (s, 1H, N=CH), 8.72 (d, *J* = 2.3 Hz, 1H, Ar), 7.80 (d, *J* = 2.3 Hz, 1H, Ar), 7.37–7.32 (m, 2H, Ar), 6.99–6.93 (m, 2H, Ar), 5.13 (t, *J* = 6.0 Hz, 1H, CH), 3.95 (s, 3H, OCH_3_), 3.85 (s, 3H, OCH_3_), 3.68 (s, 3H, OCH_3_), 2.00 (p, *J* = 7.0 Hz, 2H, CH_2_), 1.59–1.46 (m, 2H, CH_2_), 1.45–1.34 (m, 2H, CH_2_), 0.93 (t, *J* = 7.2 Hz, 3H, CH_3_); ^13^C NMR (CDCl_3_, 101 MHz): *δ* = 171.6 (C, C-2), 158.8 (C, C-12), 152.7 (C, C-16), 151.7 (C, C-5), 151.6 (C, C-6), 144.3 (C, C-13), 143.7 (C, C-8), 130.5 (C, C-10), 122.7 (C, C-14, C-18), 115.3 (C, C-9), 114.4 (C, C-15, C-17), 108.5 (C, C-7), 80.4 (CH, C-3), 56.3 (OCH_3_, C-11), 55.5 (OCH_3_, C-19), 52.1 (OCH_3_, C-1), 32.9 (CH_2_CH_2_CH_2_CH_3_, C-4), 27.1 (CH_2_CH_2_CH_2_CH_3_, C-4), 22.4 (CH_2_CH_2_CH_2_CH_3_, C-4), 13.9 (CH_2_CH_2_CH_2_CH_3_, C-4); GCMS *m/z* 430 [M]^+^ (100), 387 (8), 371 (11), 301 (10), 285 (6), 271 (5), 262 (2), 255 (17), 248 (5), 233 (59), 218 (12), 206 (7), 203 (23), 184 (6), 175 (7), 134 (8), 122 (20), 108 (6), 92 (6), 77 (7), 59 (7). Anal. Calcd for C_22_H_26_N_2_O_7_: C, 61.39; H, 6.09; N, 6.51. Found: C, 61.45; H, 6.20; N, 6.75.

### General procedure for the synthesis of methyl 2-(4-nitro-2-((phenylamino)methyl)phenoxy)alkanoate (4a–f)

To a mixture of methyl 2-(4-nitro-2-((phenylimino)methyl)phenoxy)alkanoate (**3a**–**f**) (0.58 mmol) in 1,2-dichloroethane (10 mL), sodium triacetoxyborohydride (0.88 mmol) and acetic acid (0.1 mL) were added. The mixture was stirred magnetically at room temperature for 4 h. Then, the mixture was quenched with an aqueous solution of sodium carbonate 5 % (NaHCO_3_). The DCE layer was separated and dried (MgSO_4_). The solvent was removed under reduced pressure to give the crude product. The resultant organic layer was dried, evaporated, and residue was recrystallized from hot methanol, to yield **4a**–**f**.

#### *Methyl 2-(4-nitro-2-((phenylamino)methyl)phenoxy)butanoate (****4a****)*

Yellow solid (MeOH) This compound (**4a**) was prepared from methyl 2-(4-nitro-2-((phenylimino)methyl)phenoxy)butanoate (**3a**) (0.58 mmol, 0.20 g) and sodium triacetoxyborohydride (0.88 mmol, 0.186 g) according to the general procedure. The product obtained as a yellow solid was purified from methanol. 0.12 g (60 %); mp 106–108 °C; FTIR (neat) *ν*_max_: 3406, 3075, 2979, 1750, 1593, 1330, 1204, 1085 cm^−1^; ^1^H NMR (CDCl_3_, 400 MHz): *δ* = 8.24 (d, *J* = 2.8 Hz, 1H, Ar), 8.09 (dd, *J* = 2.8, 9.0 Hz, 1H, Ar), 7.20–7.11 (m, 2H, Ar), 6.76 (d, *J* = 9.0 Hz, 1H, Ar), 6.71 (t, *J* = 7.5 Hz, 1H, Ar), 6.67– 6.61 (m, 2H, Ar), 4.84 (undivided dd, *J* = 5.3, 6.5 Hz, 1H, CH), 4.50 (d, *J* = 15.9 Hz, 1H, CHH, ½ H, NH: D_2_O exchangeable), 4.41 (d, *J* = 15.9 Hz, 1H, CHH, ½ H, NH: D_2_O exchangeable), 3.78 (s, 3H, OCH_3_), 2.25–1.92 (m, 2H, CH_2_), 1.10 (t, *J* = 7.4 Hz, 3H, CH_3_); ^13^C NMR (CDCl_3_, 101 MHz): *δ* = 170.4 (C, C-2), 160.0 (C, C-5), 147.4 (C, C-13), 141.7 (C, C-8), 129.6 (C, C-10), 129.0 (C, C-15, C-17), 124.2 (C, C-7, C-9), 117.6 (C, C-16), 112.8 (C, C-14, C-18), 110.7 (C, C-6), 77.3 (CH, C-3), 52.3 (OCH_3_, C-1), 42.8 (CH_2_, C-12), 25.7 (CH_2_CH_3_, C-4), 9.2 (CH_2_CH_3_, C-4); GCMS *m/z* 344 [M]^+^ (90), 285 (5), 243 (100), 227 (12), 197 (20), 180 (5), 167 (8), 152 (14), 134 (7), 106 (20) 93 (14), 77 (14), 59 (18); Anal. Calcd for C_18_H_20_N_2_O_5_: C, 62.78; H, 5.85; N, 8.13. Found: C, 62.65; H, 5.87; N, 8.26. **4a**·HCl a white solid mp. 106–108 °C.

#### *Methyl 2-(4-nitro-2-((phenylamino)methyl)phenoxy)pentanoate (****4b****)*

Yellow solid (MeOH) This compound (**4b**) was prepared from methyl 2-(4-nitro-2-((phenylimino)methyl)phenoxy)pentanoate (**3b**) (0.56 mmol, 0.20 g) and sodium triacetoxyborohydride (0.84 mmol, 0.178 g) according to the general procedure. The product obtained as a pale yellow solid was purified from methanol. 0.12 g (60 %); mp 74–76 °C; FTIR (neat) *ν*_max_: 3408, 3075, 2979, 1751, 1593, 1513, 1328, 1209, 1079 cm^−1^; ^1^H NMR (CDCl_3_, 400 MHz): *δ* = 8.25 (d, *J* = 2.7 Hz, 1H, Ar), 8.10 (dd, *J* = 2.7, 9.0 Hz, 1H, Ar), 7.20–7.14 (m, 2H, Ar), 6.76 (d, *J* = 9.1 Hz, 1H, Ar), 6.72 (t, *J* = 7.3 Hz, 1H, Ar), 6.67–6.62 (m, 2H, Ar), 4.872 (dd, *J* = 7.2, 5.0 Hz, 1H, CH), 4.50 (d, *J* = 16.0 Hz, 1H, CHH), 4.41 (d, *J* = 16.0 Hz, 2H; 1H, CHH, 1H NH: D_2_O exchangeable), 3.77 (s, 3H, OCH_3_), 2.11–1.94 (m, 2H, CH_2_), 1.56 (sext, *J* = 7.5 Hz, 2H, CH_2_), 0.99 (t, *J* = 7.4 Hz, 3H, CH_3_); ^13^C NMR (CDCl_3_, 101 MHz): *δ* = 171.0 (C, C-2), 160.3 (C, C-5), 147.7 (C, C-13), 142.00 (C, C-8), 129.9 (C, C-10), 129.3 (C, C-15, C-17), 124.5 (C, C-7, C-9), 117.9 (C, C-16), 113.1 (C, C-14, C-18), 110.9 (C, C-6), 76.5 (CH, C-3), 52.2 (OCH_3_, C-1), 43.1 (CH_2_, C-12), 34.5 (CH_2_CH_2_CH_3_, C-4), 18.5 (CH_2_CH_2_CH_3_, C-4), 13.7 (CH_2_CH_2_CH_3_, C-4); GCMS m/z 358 [M]^+^ (93), 329 (8), 299 (6), 243 (100), 227 (11), 197 (19), 167 (7), 152 (10), 106 (15), 93 (14), 77 (9), 55 (9); FTIR (cm^−1^): 3408, 3110–3018, 2958–2871, 1751, 1593, 1513, 1328, 1209, 1079–1059; Anal. Calcd for C_19_H_22_N_2_O_5_: C, 66.04; H, 5.85; N, 8.56. Found: C, 66.80; H, 5.72; N, 8.80. **4b**·HCl a white solid mp. 106–108 °C.

#### *Methyl 2-(4-nitro-2-((phenylamino)methyl)phenoxy)hexanoate (****4c****)*

Yellow solid (MeOH) This compound (**4c**) was prepared from methyl 2-(4-nitro-2-((phenylimino)methyl)phenoxy)hexanoate (**3c**) (0.54 mmol, 0.20 g) and sodium triacetoxyborohydride (0.81 mmol, 0.171 g) according to the general procedure. The product obtained as a pale yellow solid was purified from methanol. 0.11 g (55 %); mp 42–44 °C; FTIR (neat) *ν*_max_: 3416, 3051, 2955, 1742, 1592, 1512, 1337, 1203, 1082 cm^−1^; ^1^H NMR (CDCl_3_, 400 MHz): *δ* = 8.24 (d, *J* = 2.7 Hz, 1H, Ar), 8.09 (dd, *J* = 2.7, 9.0 Hz, 1H, Ar), 7.20–7.12 (m, 2H, Ar), 6.76 (d, *J* = 9.0 Hz, 1H, Ar), 6.71 (t, *J* = 7.3 Hz, 1H, Ar), 6.64 (d, *J* = 8.5 Hz, 2H, Ar), 4.87 (t, *J* = 6.0 Hz, 1H, CH), 4.49 (d, *J* = 15.9 Hz, 1H, CHH), 4.41 (d, *J* = 15.9 Hz, 1H, CHH), 3.77 (s, 3H, OCH_3_), 2.08–1.98 (m, 2H, CH_2_), 1.57–1.45 (m, 2H, CH_2_), 1.45–1.32 (m, 2H, CH_2_), 0.92 (t, *J* = 7.2 Hz, 3H, CH_3_); ^13^C NMR (CDCl_3_, 101 MHz): *δ* = 171.0 (C, C-2), 160.4 (C, C-5), 147.7 (C, C-13), 142.0 (C, C-8), 129.9 (C, C-10), 129.3 (C, C-15, C-17), 124.6 (C, C-9), 124.5 (C, C-7), 117.9 (C, C-16), 113.1 (C, C-14, C-18), 111.0 (C, C-6), 76.7 (CH, C-3), 52.6 (OCH_3_, C-1), 43.2 (CH_2_, C-12), 32.3 (CH_2_CH_2_CH_2_CH_3_, C-4), 27.3 (CH_2_CH_2_CH_2_CH_3_, C-4), 22.3 (CH_2_CH_2_CH_2_CH_3_, C-4), 13.8 (CH_2_CH_2_CH_2_CH_3_, C-4); GCMS *m/z* 372 [M]^+^ (90), 329 (8), 243 (100), 227 (11), 197 (17), 167 (6), 152 (8), 106 (13), 93 (15), 69 (10), 59 (6); Anal. Calcd for C_20_H_24_N_2_O_5_: C, 64.50; H, 6.50; N, 7.52. Found: C, 64.455; H, 6.62; N, 7.55. **4c**·HCl a white solid mp. 106–108 °C.

#### *Methyl 2-(2-methoxy-4-nitro-6-((phenylamino)methyl)phenoxy)butanoate (****4d****)*

Yellow solid (MeOH) This compound (**4d**) was prepared from methyl 2-(2-methoxy-4-nitro-6-((phenylimino)methyl)phenoxy)butanoate (**3d**) (0.54 mmol, 0.20 g) and sodium triacetoxyborohydride (0.81 mmol, 0.171 g) according to the general procedure. The product obtained as a pale yellow solid was purified from methanol. 0.11 g (55 %); mp 117–119 °C; FTIR (neat) *ν*_max_: 3387, 3051, 2955, 1743, 1601, 1514, 1332, 1204, 1091 cm^−1^; ^1^H NMR (CDCl_3_, 400 MHz): *δ* = 7.93 (d, *J* = 2.6 Hz, 1H, Ar), 7.69 (d, *J* = 2.6 Hz, 1H, Ar), 7.20–7.11 (m, 2H, Ar), 6.74–6.68 (m, 1H, Ar), 6.67–6.60 (m, 2H, Ar), 5.26 (undivided dd, *J* = 5.6, 6.8 Hz, 1H, CH), 4.57 (d, *J* = 16.0 Hz, 1H, CHH), 4.48 (d, *J* = 16.0 Hz, 1H, CHH), 4.38 (s, 1H, NH: D_2_O exchangeable), 3.89 (s, 3H, OCH_3_), 3.73 (s, 3H, OCH_3_), 2.10–1.92 (m, 2H, CH_2_), 1.08 (t, *J* = 7.4 Hz, 3H, CH_3_); ^13^C NMR (CDCl_3_, 101 MHz): *δ* = 171.8 (C, C-2), 150.5 (C, C-6), 149.8 (C, C-5), 147.8 (C, C-13), 143.0 (C, C-8), 133.7 (C, C-10), 129.3 (C, C-15, C-17), 117.8 (C, C-16), 116.8 (C, C-9), 113.0 (C, C-14, C-18), 107.0 (C, C-7), 80.6 (CH, C-3), 56.2 (OCH_3_, C-11), 52.1 (OCH_3_, C-1), 43.4 (CH_2_, C-12), 26.7 (CH_2_CH_3_, C-4), 9.3 (CH_2_CH_3_, C-4); GCMS *m/z* 374 [M]^+^ (62), 273 (100), 257 (37), 227 (16), 207 (7), 182 (12), 154 (7), 134 (7), 106 (17), 93 (26), 77 (12), 59 (14); Anal. Calcd for C_19_H_22_N_2_O_6_: C, 60.95; H, 5.92; N, 7.48. Found: C, 61.05; H, 5.99; N, 7.42. **4d**·HCl a white solid mp. 106–108 °C.

#### *Methyl 2-(2-methoxy-4-nitro-6-((phenylamino)methyl)phenoxy)pentanoate (****4e****)*

Yellow solid (MeOH) This compound (**4e**) was prepared from methyl 2-(2-methoxy-4-nitro-6-((phenylimino)methyl)phenoxy)pentanoate (**3e**) (0.52 mmol, 0.20 g) and sodium triacetoxyborohydride (0.78 mmol, 0.166 g) according to the general procedure. The product obtained as a pale yellow solid was purified from methanol. 0.11 g (55 %), mp 112–114 °C; FTIR (neat) *ν*_max_: 3397, 3051, 2955, 1746, 1602, 1514, 1336, 1203, 1097 cm^−1^; ^1^H NMR (CDCl_3_, 400 MHz): *δ* = 7.93 (d, *J* = 2.5 Hz, 1H, Ar), 7.68 (d, *J* = 2.5 Hz, 1H, Ar), 7.20–7.13 (m, 2H, Ar), 6.76–6.58 (m, 1H, Ar), 6.67–6.58 (m, 2H, Ar), 5.31 (t, *J* = 6.0 Hz, 1H, CH), 4.59 (d, *J* = 15.9 Hz, 1H, CHH), 4.55 (d, *J* = 15.9 Hz, 1H, CHH), 4.37 (bs, 1H, NH: D_2_O exchangeable), 3.89 (s, 3H, OCH_3_), 3.72 (s, 3H, OCH_3_), 2.03–1.88 (m, 2H, CH_2_), 1.66–1.43 (m, 2H, CH_2_), 0.97 (t, *J* = 7.4 Hz, 3H, CH_3_); ^13^C NMR (CDCl_3_, 101 MHz): *δ* = 172.0 (C, C-2), 150.5 (C, C-6), 149.7 (C, C-5), 147.8 (C, C-13), 143.0 (C, C-8), 133.7 (C, C-10), 129.3 (C, C-15, C-17), 117.8 (C, C-16), 116.8 (C, C-9), 113.0 (C, C-14, C-18), 107.0 (C, C-7), 79.4 (CH, C-3), 56.2 (OCH_3_, C-11), 52.1 (OCH_3_, C-1), 43.4 (CH_2_, C-12), 35.4 (CH_2_CH_2_CH_3_, C-4), 18.3 (CH_2_CH_2_CH_3_, C-4), 13.8 (CH_2_CH_2_CH_3_, C-4); GCMS 388 *m/z* [M]^+^ (52), 273 (100), 257 (33), 227 (14), 207 (9), 182 (10), 148 (6), 106 (15), 93 (29), 87 (5), 77 (11), 73 (7), 59 (8), 55 (12); Anal. Calcd for C_20_H_24_N_2_O_6_: C, 61.84; H, 6.23; N, 7.21. Found: C, 61.65; H, 6.28; N, 7.15. **4e**·HCl a white solid mp. 106–108 °C.

#### *Methyl 2-(2-methoxy-4-nitro-6-((phenylamino)methyl)phenoxy)hexanoate (****4f****)*

Yellow solid (MeOH) This compound (**4f**) was prepared from methyl 2-(2-methoxy-4-nitro-6-((phenylimino)methyl)phenoxy)hexanoate (**3f**) (0.50 mmol, 0.20 g) and sodium triacetoxyborohydride (0.75 mmol, 0.16 g) according to the general procedure. The product obtained as a pale yellow solid was purified from methanol. 0.15 g (85 %); mp 119–121 °C; FTIR (neat) *ν*_max_: 3385, 3091, 2953, 1746, 1601, 1514, 1334, 1202, 1095 cm^−1^; ^1^H NMR (CDCl_3_, 400 MHz): *δ* = 7.92 (d, *J* = 2.6 Hz, 1H, Ar), 7.68 (d, *J* = 2.6 Hz, 1H, Ar), 7.20–7.11 (m, 2H, Ar), 6.70 (t, *J* = 7.3 Hz, 1H, Ar), 6.63 (d, *J* = 7.8 Hz, 2H, Ar), 5.29 (undivided dd, *J* = 5.6, 6.8 Hz, 1H, CH), 4.56 (d, *J* = 15.8 Hz, 1H, CHH), 4.47 (d, *J* = 15.9 Hz, 1H, CHH), 4.36 (bs, 1H, NH: D_2_O exchangeable), 3.88 (s, 3H, OCH_3_), 3.72 (s, 3H, OCH_3_), 2.02–1.89 (m, 2H, CH_2_), 1.55–1.30 (m, 4H, C_2_H_4_), 0.90 (t, *J* = 7.2 Hz, 3H, CH_3_); ^13^C NMR (CDCl_3_, 101 MHz): *δ* = 172.0 (C, C-2), 150.5 (C, C-6), 149.8 (C, C-5), 147.8 (C, C-13), 143.0 (C, C-8), 133.7 (C, C-10), 129.3 (C, C-15, C-17), 117.8 (C, C-16), 116.8 (C, C-9), 113.0 (C, C-14, C-18), 107.0 (C, C-7), 79.7 (CH, C-3), 56.2 (OCH_3_, C-11), 52.1 (OCH_3_, C-1), 43.4 (CH_2_, C-12), 33.1 (CH_2_CH_2_CH_2_CH_3_, C-4), 27.0 (CH_2_CH_2_CH_2_CH_3_, C-4), 22.4 (CH_2_CH_2_CH_2_CH_3_, C-4), 13.9 (CH_2_CH_2_CH_2_CH_3_, C-4); GCMS 402 *m/z* [M]^+^ (46), 273 (100), 257 (32), 227 (13), 182 (9), 154 (5), 106 (12), 93 (25), 69 (9), 55 (6); Anal. Calcd for C_21_H_26_N_2_O_6_ (402.18): C, 62.67; H, 6.51; N, 6.96. Found: C, 62.72; H, 6.45; N, 6.90. **4f**·HCl a white solid mp. 106–108 °C.

### Direct reductive amination of 1a–f; synthesis of (4a–f)

Aniline (1.87 mmol), sodium triacetoxyborohydride (2.81 mmol), and acetic acid (0.2 mL) were added to a mixture of methyl 2-(2-formyl-4-nitrophenoxy)alkanoate (**1a**–**f**) (1.87 mmol) in 1,2-dichloroethane (15 mL). The mixture was stirred magnetically at room temperature for 4 h. Next, the mixture was washed (neutralized) with an aqueous solution of sodium carbonate 5 % (NaHCO_3_) (30 mL) and extracted with ethyl acetate. The resultant organic layer was dried and evaporated, and residue was recrystallized from methanol to give **4a**–**f**. Yields of **4a**–**f** are given in Scheme 4.

### General procedure for the synthesis of methyl 2-(4-amino-2-((phenylamino)methyl)phenoxy)alkanoate (5a and 5c–f)

A mixture of methanol (10 mL) and 10 % Pd/C catalyst (0.033 g) was stirred magnetically under a slow stream of hydrogen (1 bubble per second) at room temperature for 30 min. Then, a solution of **3a** or **3c**–**f** (0.91 mmol) in methanol (10 mL) and DME (10 mL) was added, and reduction was carried out until the conversion of **3a** or **3c**–**f** was completed (7 h), which was determined by gas chromatography. The mixture was left overnight, the catalyst was separated on the next day, the solvent was evaporated, and the residue was obtained to give **5a** and **5c**–**f**.

#### *Methyl 2-(4-amino-2-((phenylamino)methyl)phenoxy)butanoate (****5a****)*

Brown semi-solid This compound (**5a**) was prepared from methyl 2-(4-nitro-2-((phenylimino)methyl)phenoxy)butanoate (**3a**) (0.91 mmol, 0.31 g) and 10 % Pd/C catalyst (0.031 g) according to the general procedure. The product obtained as a brown semi-solid 0243 g (85 %); FTIR (neat) *ν*_max_: 3365, 3019, 2935, 1736, 1599, 1202, 1058 cm^−1^; ^1^H NMR (CDCl_3_, 400 MHz): *δ* = 7.18–7.09 (m, 2H, Ar), 6.72–6.54 (m, 5H, Ar), 6.46 (dd, *J* = 2.7, 8.5 Hz, 1H, Ar), 4.59 (t, *J* = 6.0 Hz, 1H, CH), 4.36 (d, *J* = 14.6 Hz, 1H, CHH), 4.26 (d, *J* = 14.6 Hz, 1H, CHH), 3.90–3.53 (m, 5H; 3H, OCH_3_, 2H NH_2_: D_2_O exchangeable), 2.01–1.91 (m, 2H, CH_2_), 1.04 (t, *J* = 7.4 Hz, 3H, CH_3_); ^13^C NMR (CDCl_3_, 101 MHz): *δ* = 172.5 (C, C-2), 148.6 (C, C-5), 148.5 (C, C-13), 140.7 (C, C-8), 129.4 (C, C-10), 129.1 (C, C-15, C-17), 117.2 (C, C-16), 116.7 (C, C-9), 114.2 (C, C-7), 113.2 (C, C-6), 113.1 (C, C-14, C-18), 77.9 (CH, C-3), 52.1 (OCH_3_, C-1), 43.7 (CH_2_, C-12), 26.3 (CH_2_CH_3_, C-4), 9.6 (CH_2_CH_3_, C-4); GCMS 314 *m/z* [M]^+^ (100), 213 (46), 196 (30), 162 (36) 134 (16), 122 (59), 106 (11), 104 (25), 93 (24), 77 (18), 59 (9); Anal. Calcd for C_18_H_22_N_2_O_3_: C, 68.77; H, 7.05; N, 8.91. Found: C, 68.64; H, 7.15; N, 8.95.

#### *Methyl 2-(4-amino-2-((phenylamino)methyl)phenoxy)hexanoate (****5c****)*

Brown semi-solid This compound (**5c**) was prepared from methyl 2-(4-nitro-2-((phenylimino)methyl)phenoxy)hexanoate (**3c**) (0.54 mmol, 0.20 g) and 10 % Pd/C catalyst (0.02 g) according to the general procedure. The product obtained as a brown semi-solid 0.151 g (82 %); FTIR (neat) *ν*_max_: 3365, 3019, 2935, 1738, 1600, 1201, 1063 cm^−1^; ^1^H NMR (CDCl_3_, 400 MHz): *δ* = 7.19–7.10 (m, 2H, Ar), 6.72–6.54 (m, 5H, Ar), 6.46 (dd, *J* = 2.8, 8.5 Hz, 1H, Ar), 4.63 (undivided dd, *J* = 5.6, 6.8 Hz, 1H, CH), 4.35 (d, *J* = 14.5 Hz, 1H, CHH), 4.26 (d, *J* = 14.5 Hz, 1H, CHH), 3.71 (s, 4H; 3H, OCH_3_, 1H NH: D_2_O exchangeable), 1.98–1.87 (m, 2H, CH_2_), 1.51–1.41 (m, 2H, CH_2_), 1.40–1.27 (m, 2H, CH_2_), 0.88 (t, *J* = 7.2 Hz, 3H, CH_3_); ^13^C NMR (CDCl_3_, 101 MHz): *δ* = 172.8 (C, C-2), 148.6 (C, C-5), 148.6 (C, C-13), 140.7 (C, C-8), 129.4 (C, C-10), 129.1 (C, C-15, C-17), 117.2 (C, C-16), 116.7 (C, C-9), 114.2 (C, C-7), 113.2 (C, C-6), 113.1 (C, C-14, C-18), 76.9 (CH, C-3), 52.1 (OCH_3_, C-1), 43.7 (CH_2_, C-12), 32.7 (CH_2_CH_2_CH_2_CH_3_, C-4), 27.4 (CH_2_CH_2_CH_2_CH_3_, C-4), 22.4 (CH_2_CH_2_CH_2_CH_3_, C-4), 13.9 (CH_2_CH_2_CH_2_CH_3_, C-4); GCMS 342 *m/z* [M]^+^ (100), 213 (63), 196 (35), 162 (9), 134 (7), 122 (49), 106 (11), 104 (26), 93 (27), 77 (17), 55 (7); Anal. Calcd for C_20_H_26_N_2_O_3_: C, 70.15; H, 7.65; N, 8.18. Found: C, 70.35; H, 7.72; N, 8.16.

#### *Methyl 2-(4-amino-2-methoxy-6-((phenylamino)methyl)phenoxy)butanoate (****5d****)*

Brown semi-solid This compound (**5d**) was prepared from methyl 2-(2-methoxy-4-nitro-6-((phenylimino)methyl)phenoxy)butanoate (**3d**) (0.54 mmol, 0.20 g) and 10 % Pd/C catalyst (0.02 g) according to the general procedure. The product obtained as a brown semi-solid 0.132 g (71 %); FTIR (neat) *ν*_max_: 3365, 3019, 2935, 1737, 1599, 1199, 1054 cm^−1^; ^1^H NMR (CDCl_3_, 400 MHz): *δ* = 7.28–6.99 (m, 2H, Ar); 6.81–6.57 (m, 3H, Ar), 6.24 (d, *J* = 2.5 Hz, 1H, Ar), 6.13 (d, *J* = 2.5 Hz, 1H, Ar), 4.72–4.66 (m, 1H, CH), 4.51–4.29 (m, 2H, CH_2_), 3.76–3.54 (m, 8H; 6H OCH_3_, 2H NH_2_: D_2_O exchangeable), 2.03-1.83 (m, 2H, CH_2_), 1.08–0.83 (m, 4H, 3H CH_3_, 1H NH); ^13^C NMR (CDCl_3_, 101 MHz): *δ* = 172.8 (C, C-2), 152.1 (C, C-6), 148.5 (C, C-13), 142.8 (C, C-8), 137.3 (C, C-5), 133.5 (C, C-10), 129.1 (C, C-15, C-17), 117.1 (C, C-16), 112.9 (C, C-14, C-18), 106.8 (C, C-9), 99.2 (C, C-7), 81.6 (CH, C-3), 55.6 (OCH_3_, C-11), 51.7 (OCH_3_, C-1), 43.4 (CH_2_, C-12), 26.5 (CH_2_CH_3_, C-4), 9.2 (CH_2_CH_3_, C-4); Anal. Calcd for C_19_H_24_N_2_O_4_: C, 66.26; H, 7.02; N, 8.13. Found: C, 66.36; H, 7.14; N, 8.23.

#### *Methyl 2-(4-amino-2-methoxy-6-((phenylamino)methyl)phenoxy)pentanoate (****5e****)*

Brown semi-solid This compound (**5e**) was prepared from methyl 2-(2-methoxy-4-nitro-6-((phenylimino)methyl)phenoxy)pentanoate (**3e**) (0.93 mmol, 0.36 g) and 10 % Pd/C catalyst (0.036 g) according to the general procedure. The product obtained as a brown semi-solid 0.28 g (84 %); FTIR (neat) *ν*_max_: 3370, 3048, 2956, 1739, 1599, 1198, 1057 cm^−1^; ^1^H NMR (CDCl_3_, 400 MHz): *δ* = 7.27–7.02 (m, 2H, Ar), 6.71–6.57 (m, 3H, Ar), 6.24 (d, *J* = 2.5 Hz, 1H,Ar), 6.12 (d, *J* = 2.5 Hz, 1H, Ar), 4.74 (t, *J* = 6.2 Hz, 1H, CH), 4.43–4.32 (m, 2H, CH_2_), 3.78–3.63 (m, 8H; 6H, OCH_3_, 2H, NH_2_: D_2_O exchangeable), 2.10–1.73 (m, 2H, CH_2_), 1.73–1.35 (m, 2H, CH_2_), 1.06–0.78 (m, 4H; 3H, CH_3_, 1H, NH: D_2_O exchangeable); ^13^C NMR (CDCl_3_, 101 MHz): *δ* = 173.0 (C, C-2), 152.1 (C, C-6), 148.5 (C, C-13), 142.8 (C, C-8), 137.2 (C, C-5), 133.5 (C, C-10), 129.1 (C, C-15, C-17), 117.1 (C, C-16), 112.9 (C, C-14, C-18), 106.8 (C, C-9), 99.2 (C, C-7), 80.3 (CH, C-3), 55.6 (OCH_3_, C-11), 51.7 (OCH_3_, C-1), 43.4 (CH_2_, C-12), 35.4 (CH_2_CH_2_CH_3_, C-4), 18.1 (CH_2_CH_2_CH_3_, C-4), 13.9 (CH_2_CH_2_CH_3_, C-4);. Anal. Calcd for C_20_H_26_N_2_O_4_: C, 67.02; H, 7.31; N, 7.82. Found: C, 67.12; H, 7.37; N, 7.76.

#### *Methyl 2-(4-amino-2-methoxy-6-((phenylamino)methyl)phenoxy)hexanoate (****5f****)*

Brown semi-solid This compound (**5f**) was prepared from methyl 2-(2-methoxy-4-nitro-6-((phenylimino)methyl)phenoxy)hexanoate (**3f**) (0.40 mmol, 0.16 g) and 10 % Pd/C catalyst (0.016 g) according to the general procedure. The product obtained as a brown semi-solid 0.11 g (74 %); FTIR (neat) *ν*_max_: 3370, 3048, 2956, 1740, 1599, 1196, 1055 cm^−1^; ^1^H NMR (CDCl_3_, 400 MHz): *δ* = 7.27–6.96 (m, 2H, Ar), 6.87–6.55 (m, 3H, Ar), 6.24 (d, *J* = 2.5 Hz, 1H, Ar), 6.13 (d, *J* = 2.5 Hz, 1H, Ar), 4.73 (t, *J* = 6.2 Hz, 1H, CH), 4.40–4.29 (m, 2H, CH_2_), 3.83–3.62 (m, 8H; 6H, OCH_3_, 2H, NH_2_: D_2_O exchangeable), 1.98–1.81 (m, 2H, CH_2_), 1.51–1.21 (m, 5H; 4H, C_2_H_4_, 1H, NH: D_2_O exchangeable), 0.87 (t, *J* = 7.1 Hz, 3H; CH_3_); ^13^C NMR (CDCl_3_, 101 MHz): *δ* = 173.0 (C, C-2), 152.1 (C, C-6), 148.6 (C, C-13), 142.8 (C, C-8), 137.3 (C, C-5), 133.5 (C, C-10), 129.1 (C, C-15, C-17), 117.1 (C, C-16), 112.9 (C, C-14, C-18), 106.9 (C, C-9), 99.3 (C, C-7), 80.5 (CH, C-3), 55.6 (OCH_3_, C-11), 51.7 (OCH_3_, C-1), 43.4 (CH_2_, C-12), 33.1 (CH_2_CH_2_CH_2_CH_3_, C-4), 26.9 (CH_2_CH_2_CH_2_CH_3_, C-4), 22.5 (CH_2_CH_2_CH_2_CH_3_, C-4), 13.9 (CH_2_CH_2_CH_2_CH_3_, C-4); Anal. Calcd for C_21_H_28_N_2_O_4_ (372.2): C, 67.72; H, 7.58; N, 7.52. Found: C, 67.68; H, 7.49; N, 7.51.

### Microbiological work

#### Microorganisms

The antimicrobial activity of the synthesized compounds was assessed against 11 microbial strains: 5 Gram-negative, *Escherichia coli* American Type Culture Collection (ATTC) 25922, *Escherichia coli* (ATCC 8739), *Pseudomonas aeruginosa* (ATCC 27853), *Pseudomonas aeruginosa* (wild-type strain isolated from clinical sample), *Acinetobacter baumannii* (wild-type strain isolated from clinical sample), and 6 Gram-positive, *Enterococcus faecalis* (ATCC 29212), *Staphylococcus aureus* MSSA (ATCC 25923), *Staphylococcus aureus* MRSA (ATCC 43300), *Staphylococcus aureus* MLSB of inducible phenotype with resistance to macrolide–lincosamide–streptogramin B antibiotics (wild-type strain isolated from clinical sample), *Micrococcus luteus* Polish Collection of Microorganisms (PCM) 1944, *Streptococcus mutans* (wild-type strain isolated from dental plaque). Bacterial strains were cultivated in Brain Heart Infusion (BHI, Oxoid) medium at 37 °C for 24 h. After incubation, microbial suspension was diluted with sterile phosphate-buffered saline (PBS) to 10^8^ CFU/mL (turbidity = McFarland barium sulfate standard 0.5).

## Agar diffusion disk method

The antimicrobial activities of synthesized compounds were determined by disk diffusion method as recommended by National Committee for Clinical Laboratory Standards (NCCLS) (Furtado and Medeiros, 1980). The compounds were evaluated for antimicrobial activity against bacteria on Müeller-Hinton Agar (MHA, Oxoid) medium. The sterile 6-mm filter paper disks (Whatman, no. 2, Sigma-Aldrich) were impregnated with 10 µL of the tested compound at concentration 100 mg/mL in acetone (POCH) or DMSO (Sigma-Aldrich). The concentration of each tested compound on a disk was 10 mg/mL. The disks were allowed to remain at room temperature until complete diluent evaporation. The disks loaded with tested compounds were placed onto the surface of the proper agar medium (MHA) seeded with the suspension of appropriate test microorganism and incubated for 24 h at 36 °C. The solvent acetone or DMSO was used as a vehicle control. Commercial antibiotic ciprofloxacin (5 mg) (Oxoid) was used as a positive control. The average diameters of the zone of inhibition (in mm) were calculated for each tested sample and control. Tests were performed in triplicate.

## Minimal inhibitory concentration (MIC) assay

Compounds that exhibited activity against specific species of bacteria, as determined by the agar diffusion disk method, were further evaluated for their minimal inhibitory concentrations (MIC) using the serial dilution technique. Minimal inhibitory concentrations (MICs) were obtained by measuring the areas of the microbial growth inhibition using the bioautographic assay as described below. Prior to this study, compounds were dissolved in DMSO to make a 300 mg/mL stock and then diluted to 200, 100, 50, 25, 10, 5 and 1 mg/mL. MIC values were defined as the lowest concentration of each compound that completely inhibited microbial growth. The results were expressed in milligrams per milliliter.

## Bioautographic assay

The bioautographic assay was performed as described by (Valgas *et al.*, 2007). Plates of silica gel (0.2 mm, Sigma-Aldrich) were seeded in dot blot with 10 µL of each compound at concentration 100 mg/mL in acetone (POCH) or DMSO (Sigma-Aldrich). The concentration of each tested compound in a spot was 10 mg/mL. Sample spots were placed with a micropipette, and the spot diameter was approximately 7 mm. Each sample spot was located approximately 3 cm apart and away from the edge of TLC plate. As positive control, a solution of 2 mg of chloramphenicol (Fluka) dissolved in 1 mL of DMSO (Sigma-Aldrich) was used. As a vehicle control, the solvent acetone or DMSO was used. The controls were applied to plates in the same fashion as tested compounds. The TLC plates were air-dried, covered with MHA (10 mL of the medium on 9 cm diameter Petri plats) containing 100 mL of test microorganism suspensions, and incubated for 24 h at 36 °C. After incubation, in order to visualize zones of growth inhibition, 2 mL of MTT solution (1 mg of 3-(4,5-dimethylthiazol-2-yl)-2,5-diphenyltetrazolium bromide in one mL sterile physiological solution, Sigma-Aldrich) was added to each Petri plate and incubation was continued for 3 h at the same culture condition. In the last step, the areas of growth inhibition were measured for each tested compound and controls and the average diameters of the zone of inhibition (in mm) were calculated. Tests were performed in triplicate.

## Electronic supplementary material

Supplementary material 1 (DOC 4444 kb)
