# Supplementary material for: Synthesis and antibacterial activity of Schiff bases and amines derived from alkyl 2-(2-formyl-4-nitrophenoxy)alkanoates
Source: Med Chem Res. 2015 Jul 15;24(9):3561–77. doi: 10.1007/s00044-015-1397-6 (PMC4513202; doi:10.1007/s00044-015-1397-6)
Supplement: Supplementary file 1 — Supplementary material 1 (DOC 4444 kb) [file 44_2015_1397_MOESM1_ESM.doc]

**Synthesis and antibacterial activity of Schiff bases and amines derived from alkyl 2-(2-formyl-4-nitrophenoxy)alkanoates**

Agata Goszczyńska, Halina Kwiecień, Karol Fijałkowska

**Supporting Information**

| **3a** | **Methyl 2-(4-nitro-2-((phenylimino)methyl)phenoxy)butanoate** |
| --- | --- |
|  | **^1^H NMR** |
| **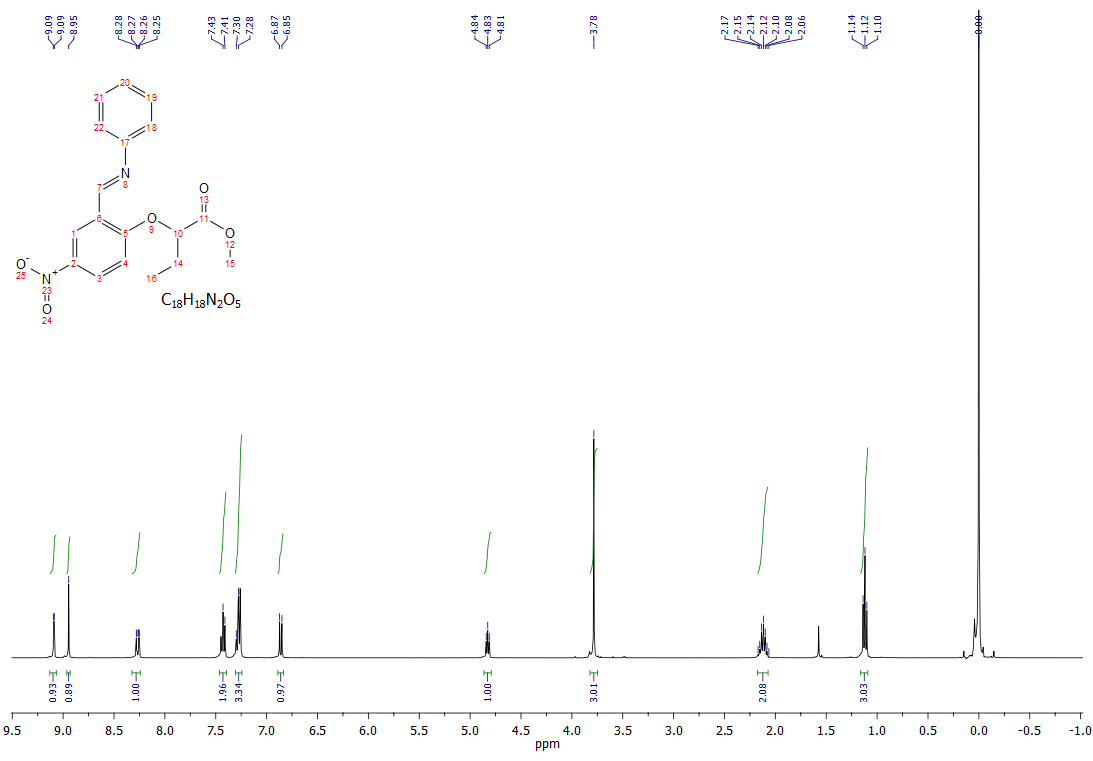** | |

|  | **^13^C NMR** |
| --- | --- |
| **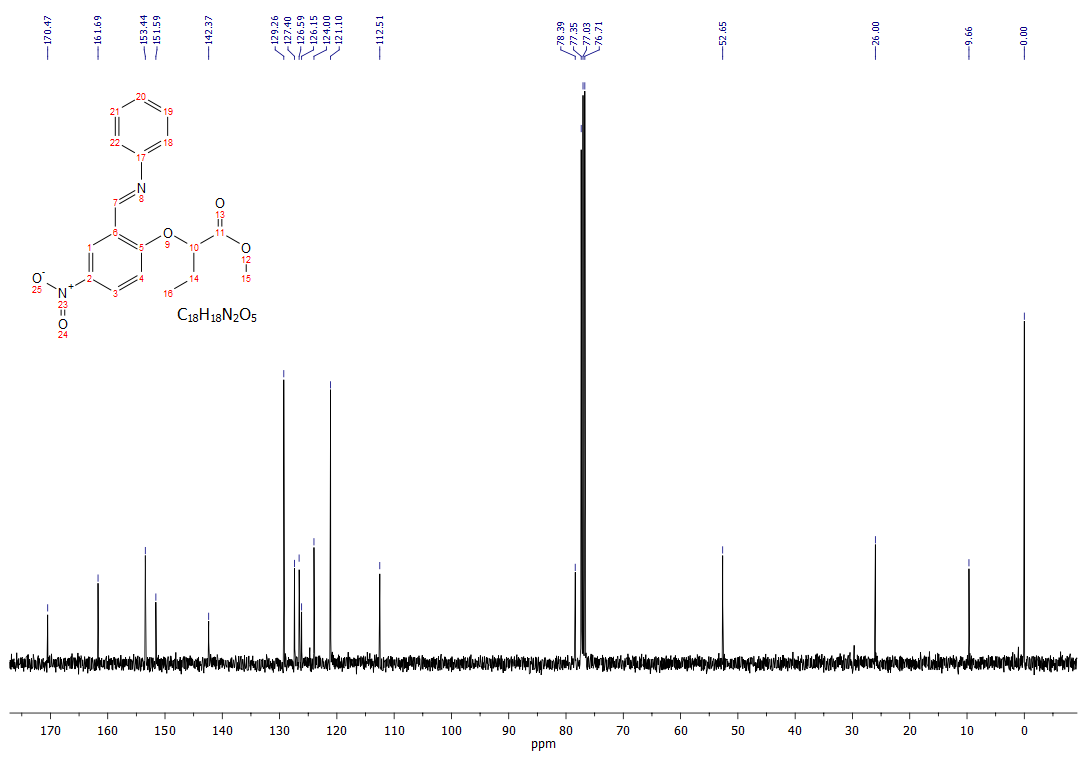** | |

|  | **GC-MS** |
| --- | --- |
| **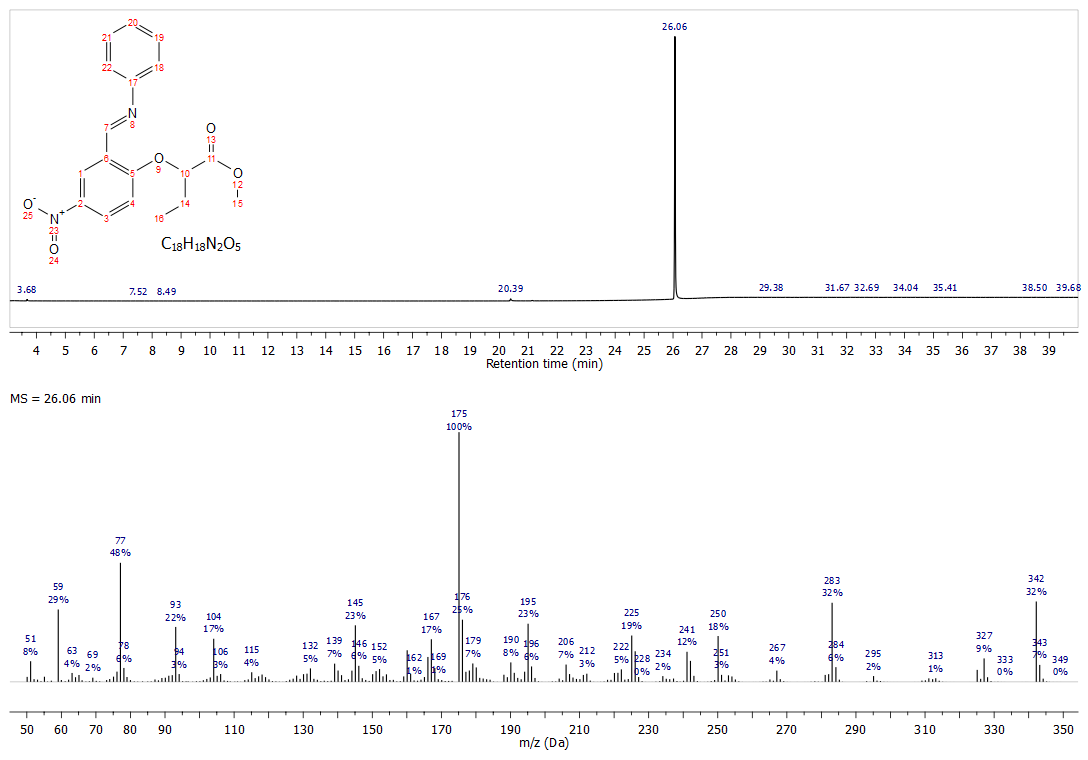** | |

|  | **FTIR** |
| --- | --- |
| **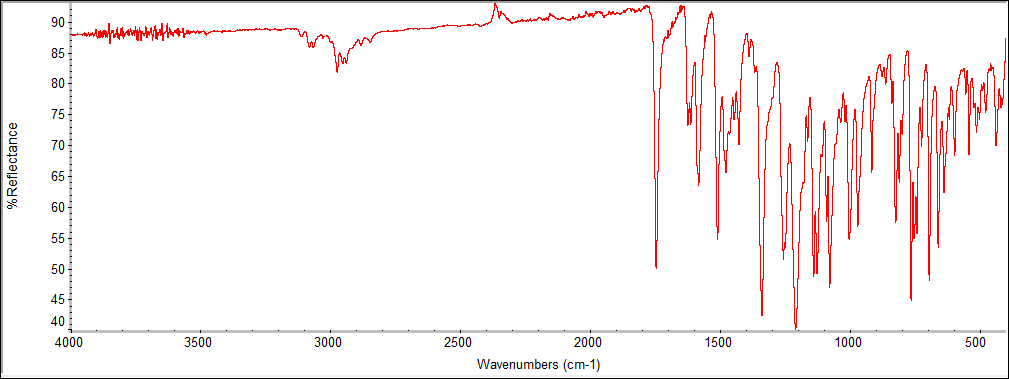** | |

| **3b** | **Methyl 2-(4-nitro-2-((phenylimino)methyl)phenoxy)pentanoate** |
| --- | --- |
|  | **^1^H NMR** |
|  | **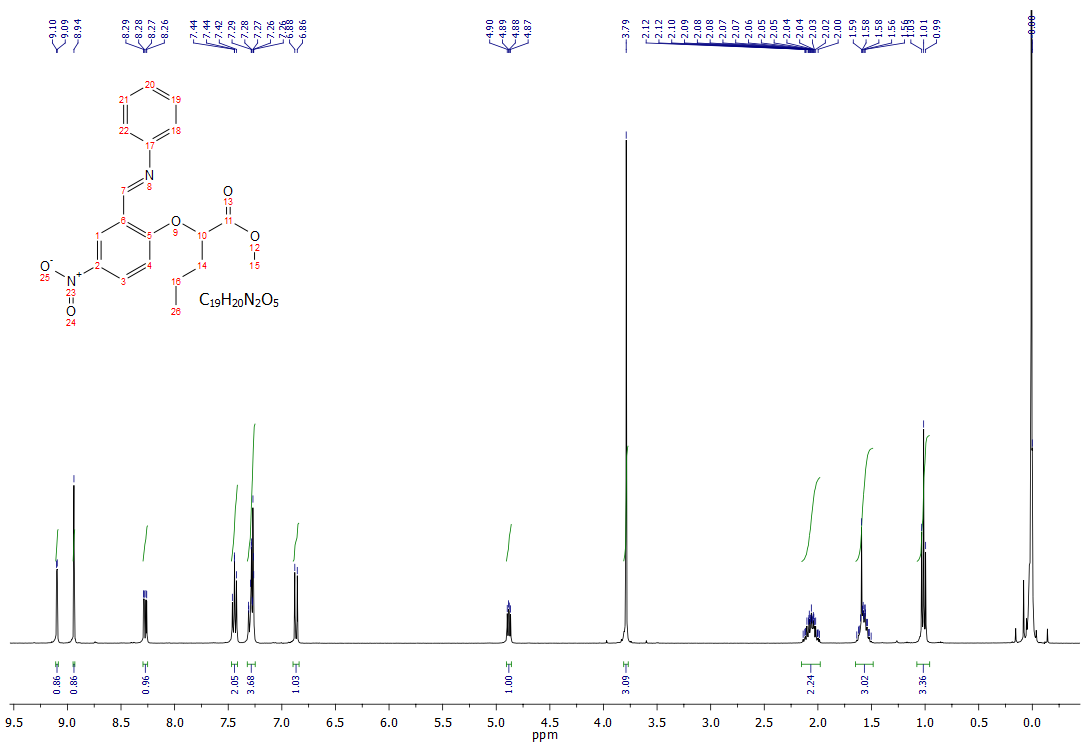** |
|  |  |

|  | **^13^C NMR** |
| --- | --- |
|  | **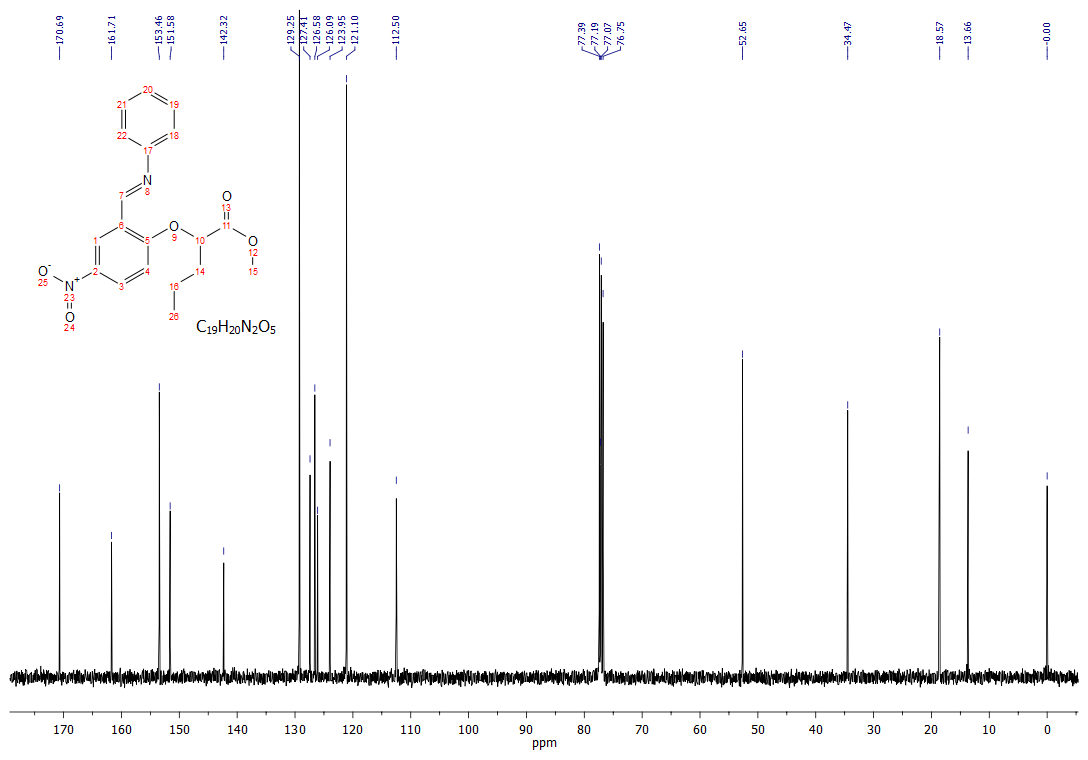** |

|  | **GC-MS** |
| --- | --- |
|  | **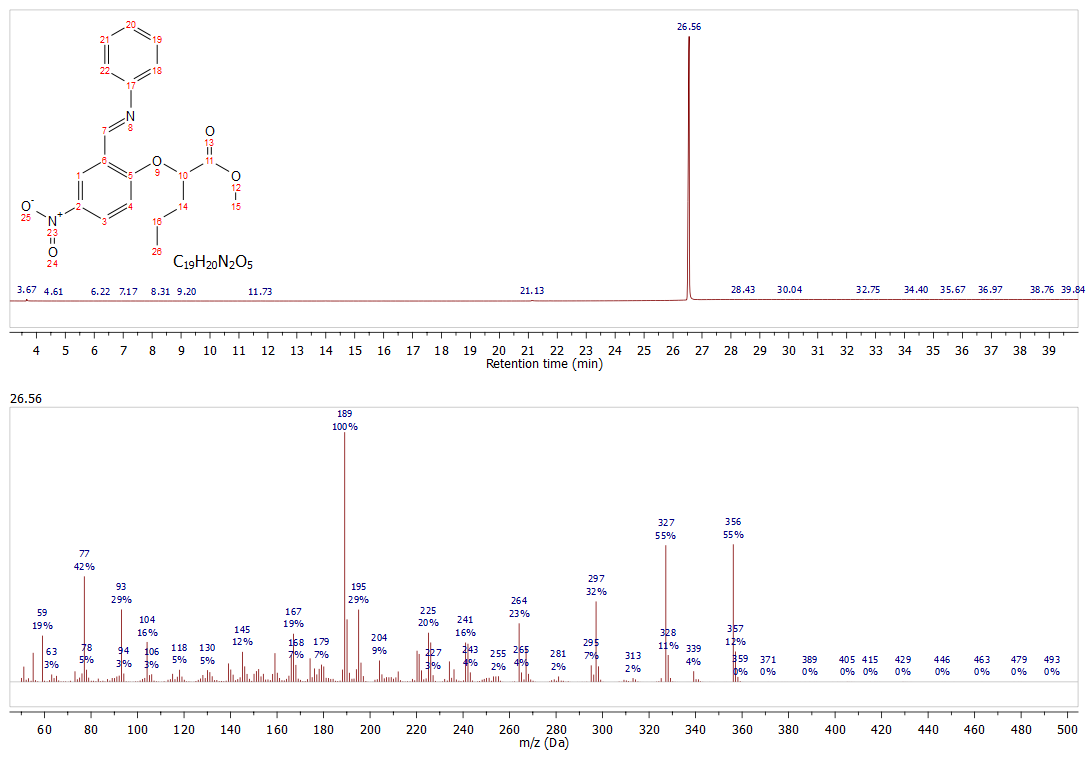** |

|  | **FTIR** |
| --- | --- |
|  | **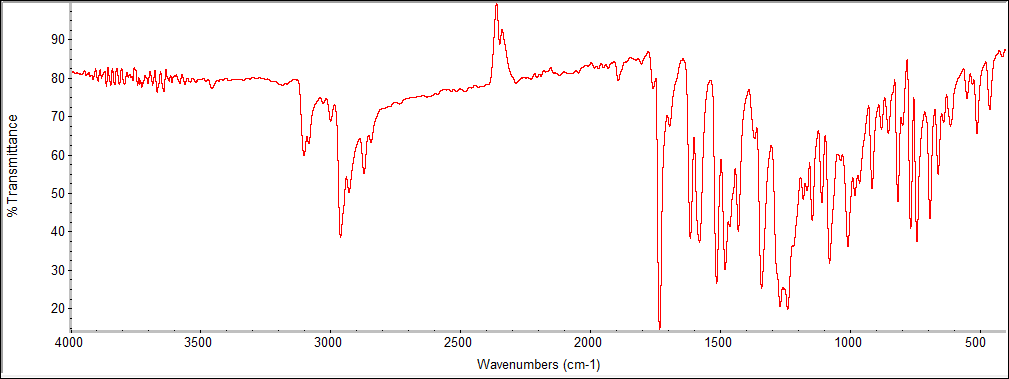** |

| **3c** | **Methyl 2-(4-nitro-2-((phenylimino)methyl)phenoxy)hexanoate** |
| --- | --- |
|  | **^1^H NMR** |
|  | **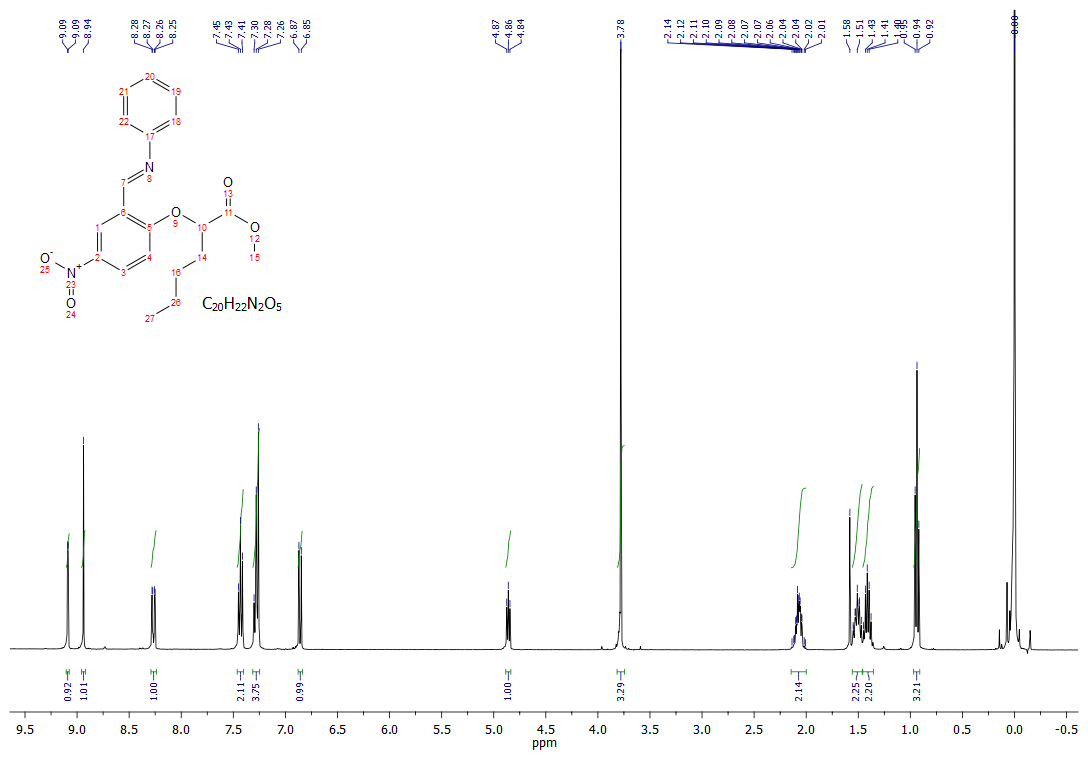** |

|  | **^13^C NMR** |
| --- | --- |
|  | **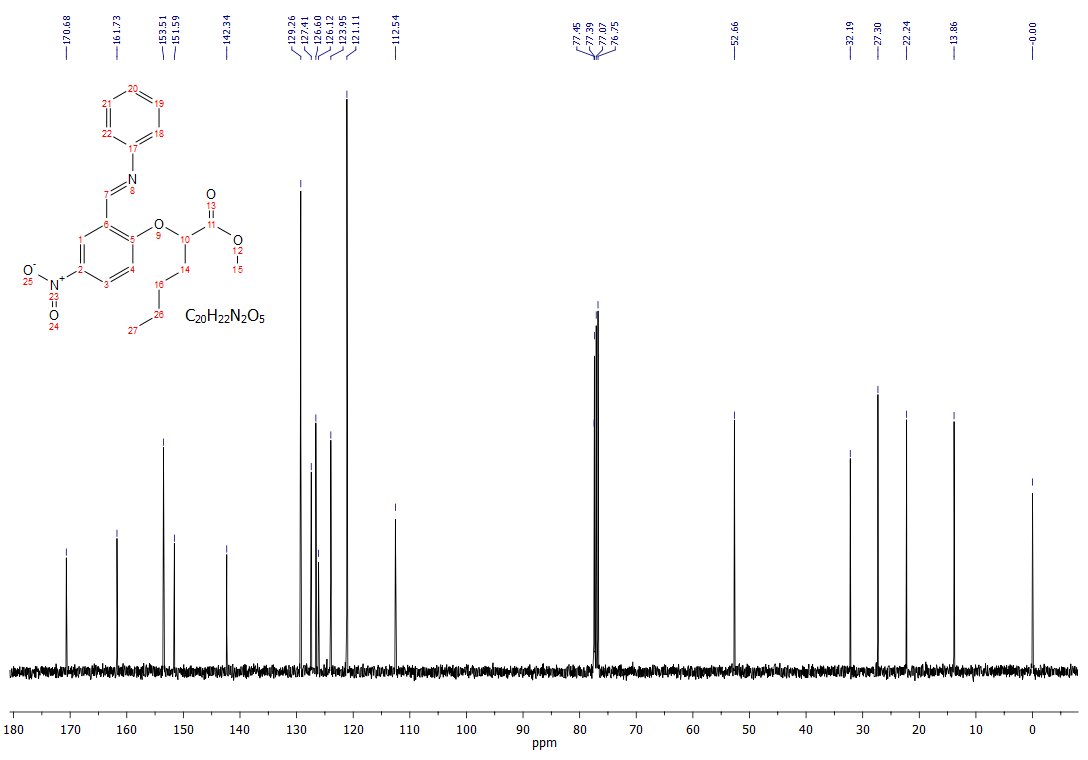** |

|  | **GC-MS** |
| --- | --- |
|  | **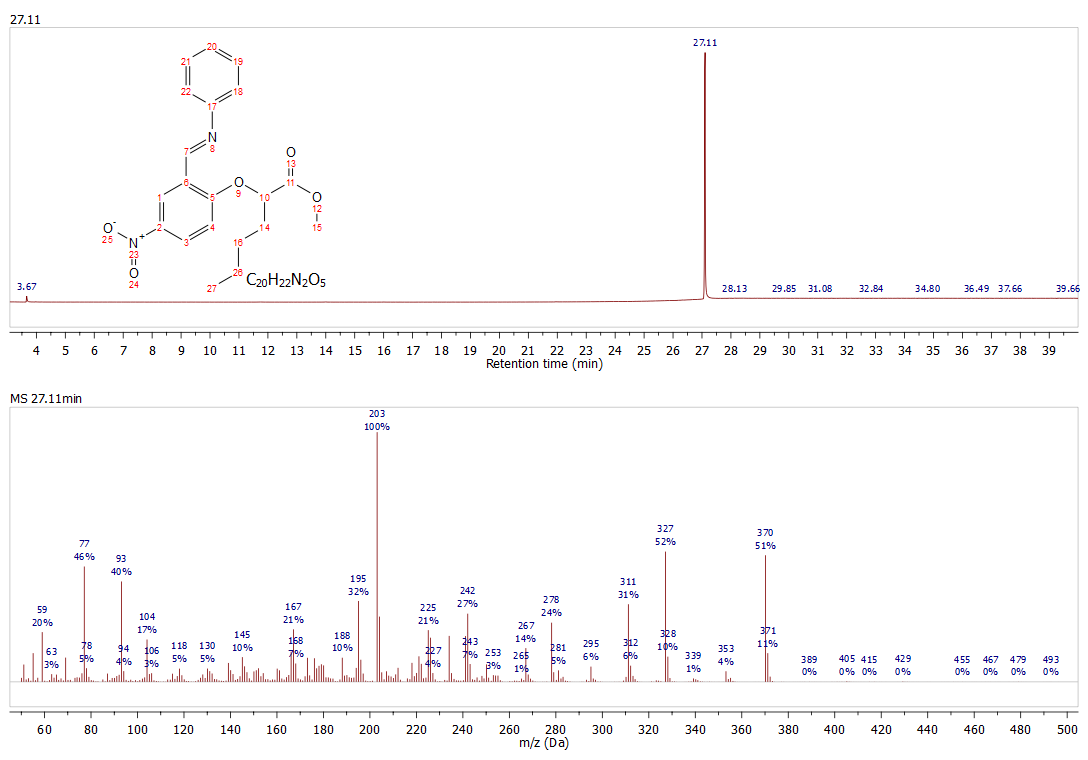** |

|  | **FTIR** |
| --- | --- |
|  | **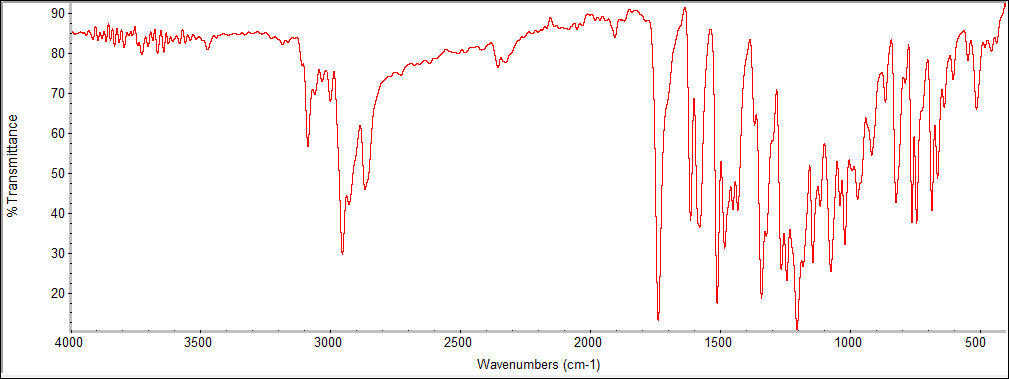** |

| **3d** | **Methyl 2-(2-methoxy-4-nitro-6-((phenylimino)methyl)phenoxy)butanoate** |
| --- | --- |
|  | **^1^H NMR** |
|  | **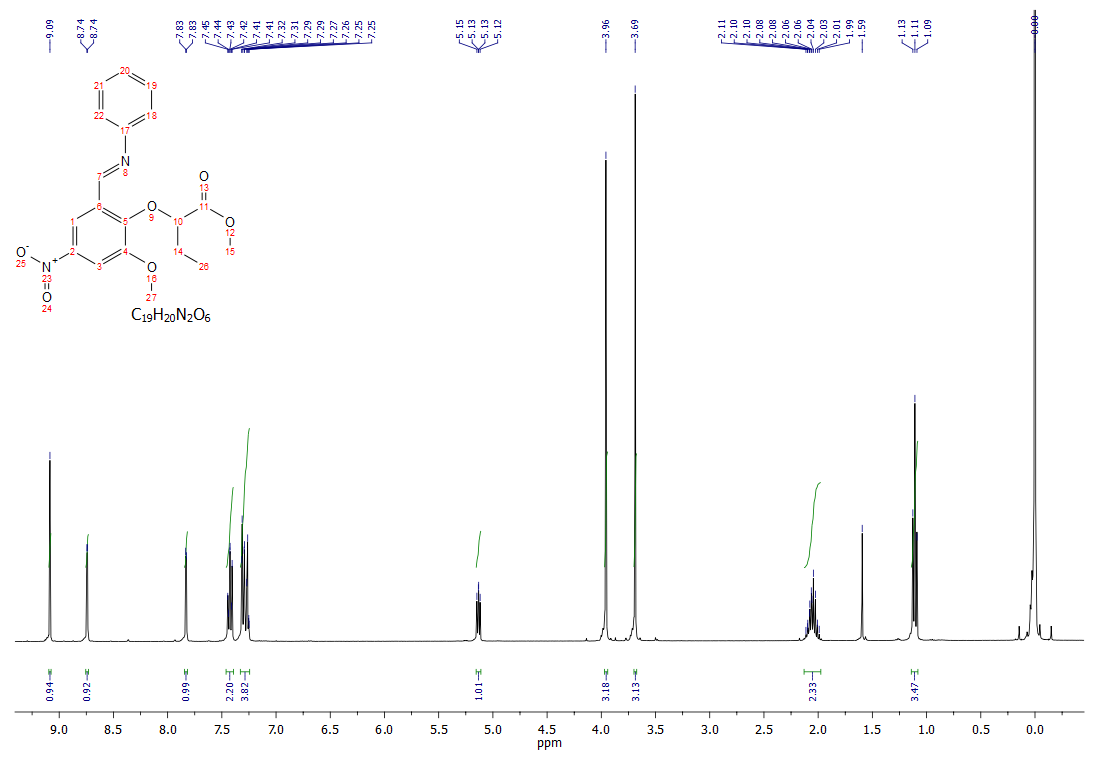** |
|  | **^13^C NMR** |
|  | **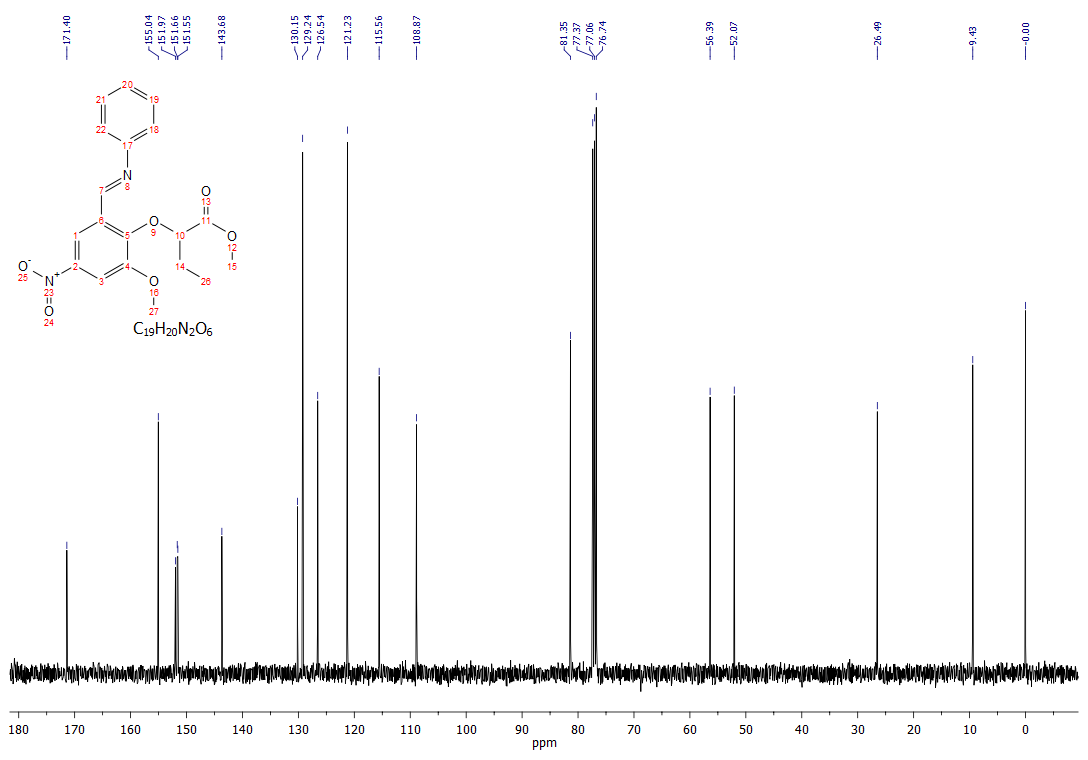** |

|  | **GC-MS** |
| --- | --- |
|  | **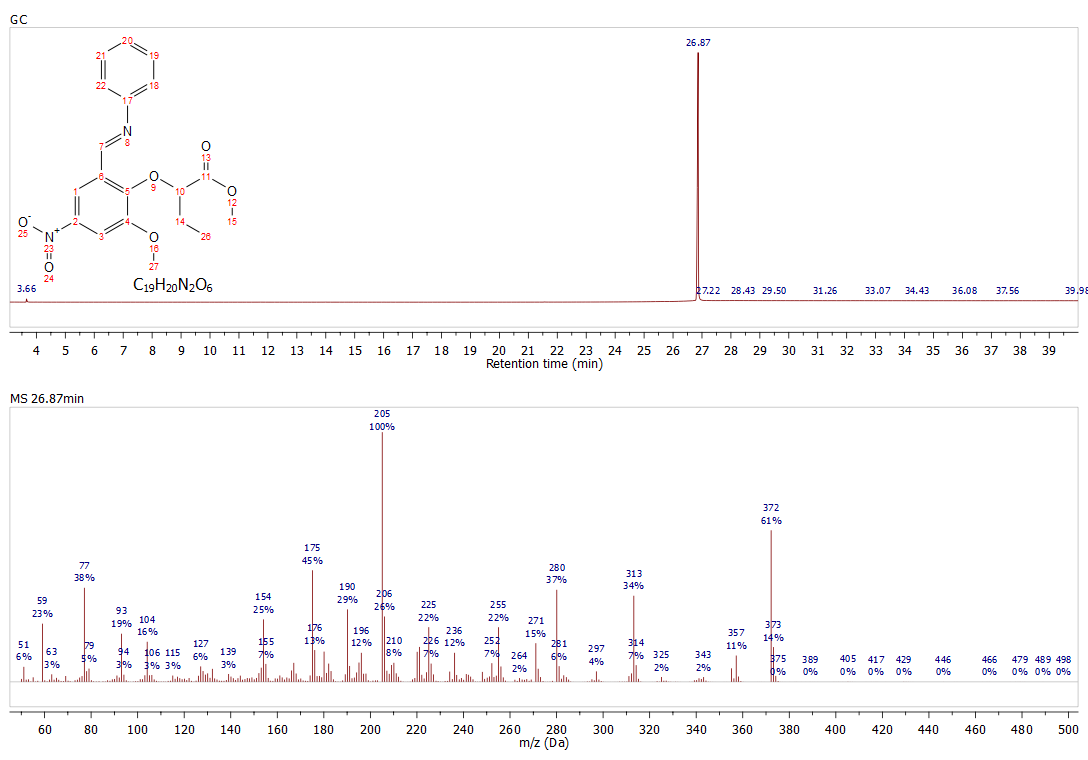** |

|  | **FTIR** |
| --- | --- |
|  | **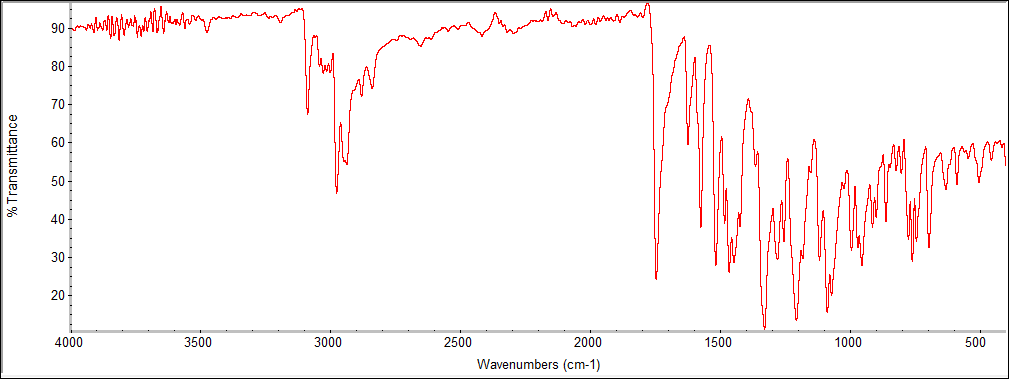** |

| **3e** | **Methyl 2-(2-methoxy-4-nitro-6-((phenylimino)methyl)phenoxy)pentanoate** |
| --- | --- |
|  | **^1^H NMR** |
|  | **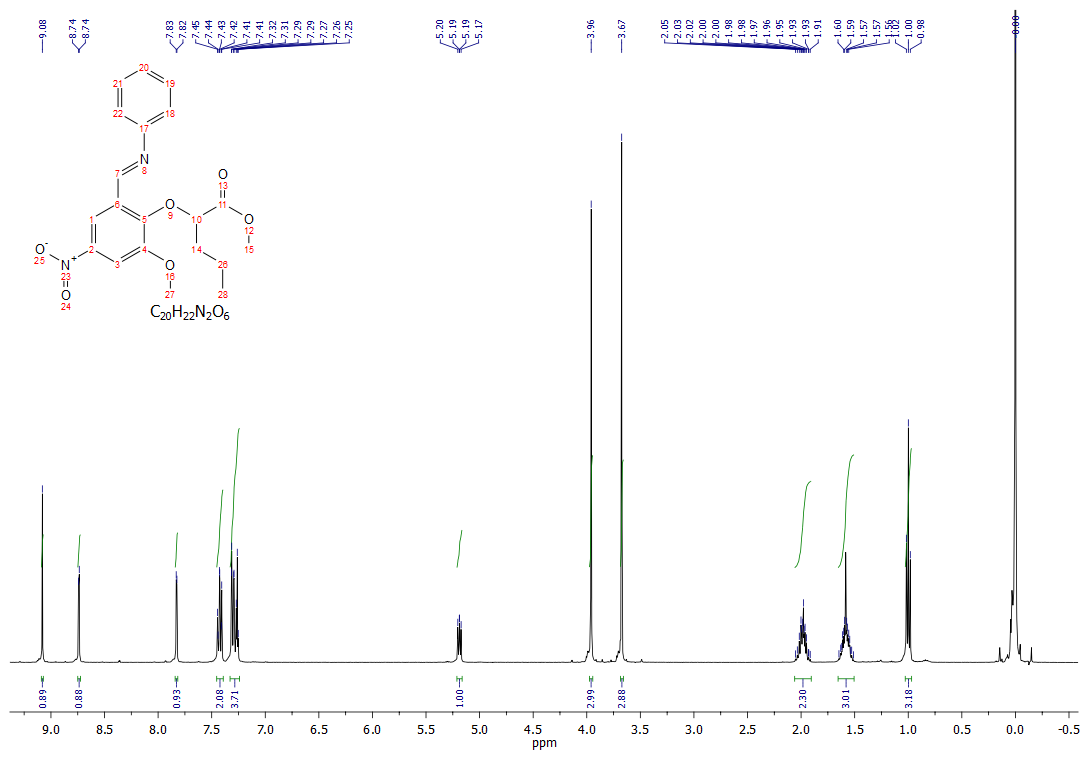** |

|  | **^13^C NMR** |
| --- | --- |
|  | **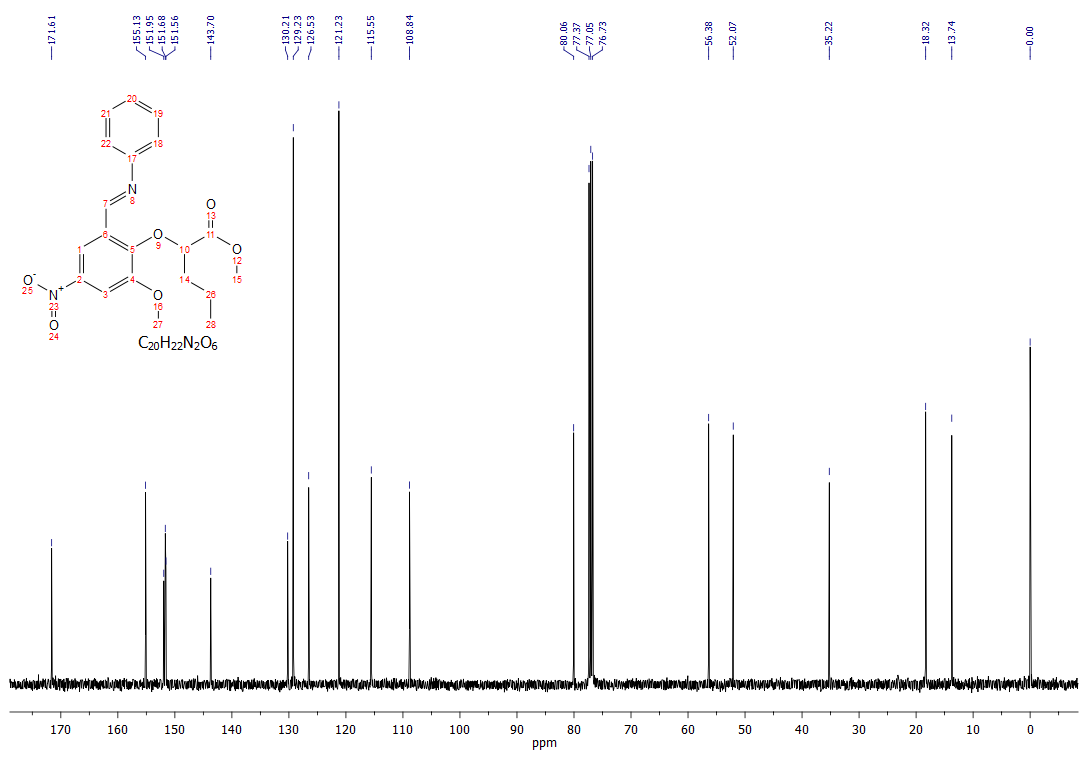** |

|  | **GC-MS** |
| --- | --- |
|  | **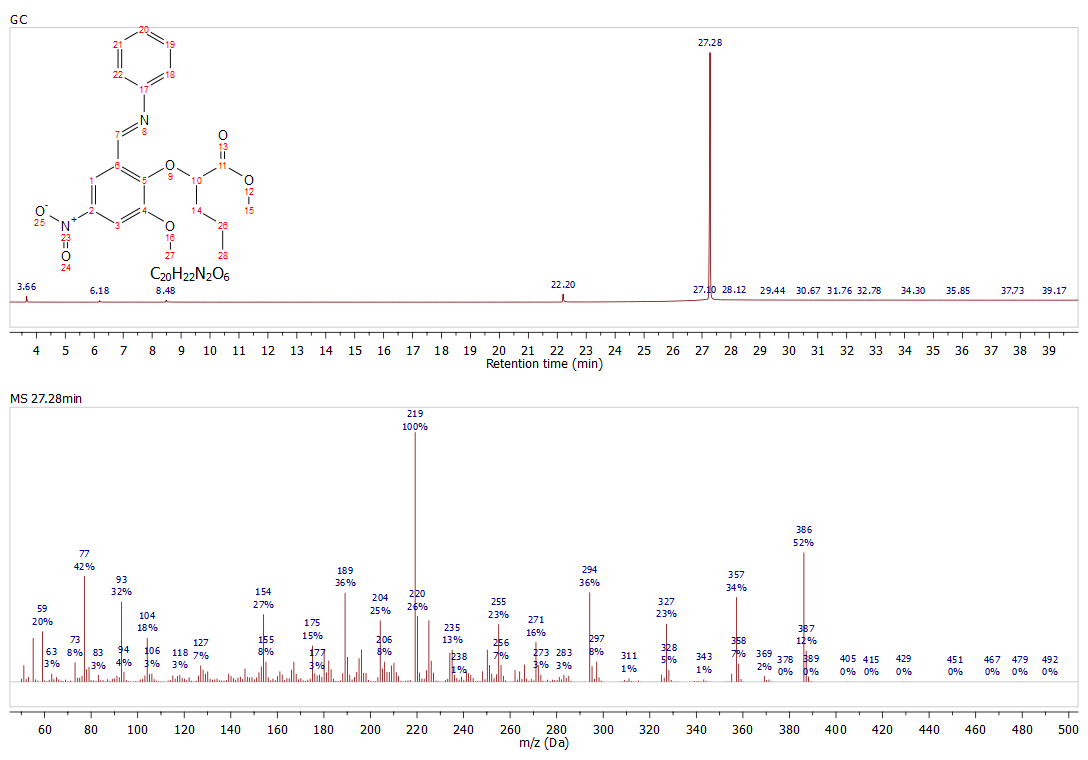** |

|  | **FTIR** |
| --- | --- |
|  | **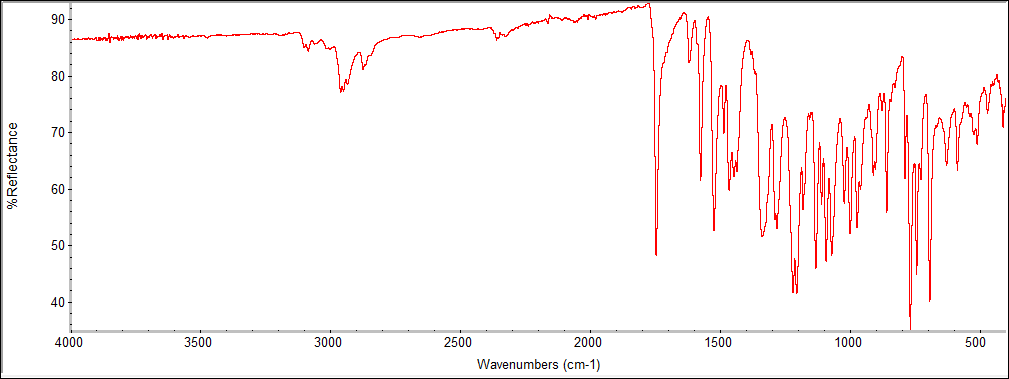** |

| **3f** | **Methyl 2-(2-methoxy-4-nitro-6-((phenylimino)methyl)phenoxy)hexanoate** |
| --- | --- |
|  | **^1^H NMR** |
|  | **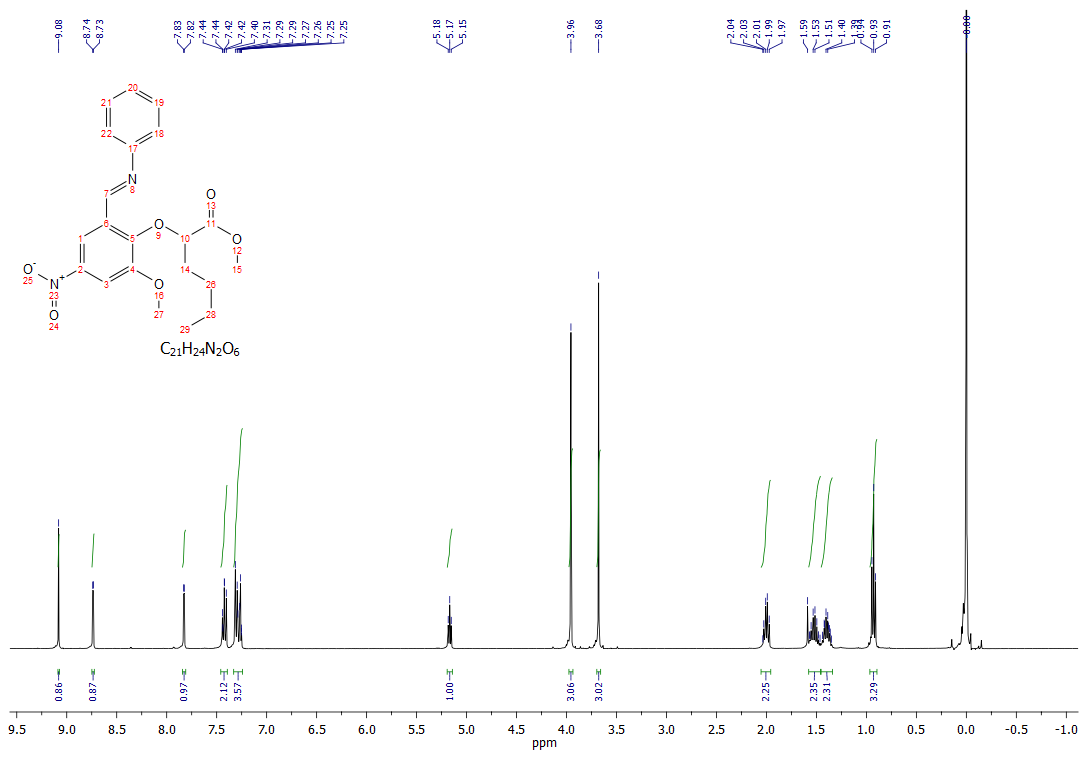** |

|  | **^13^C NMR** |
| --- | --- |
|  | **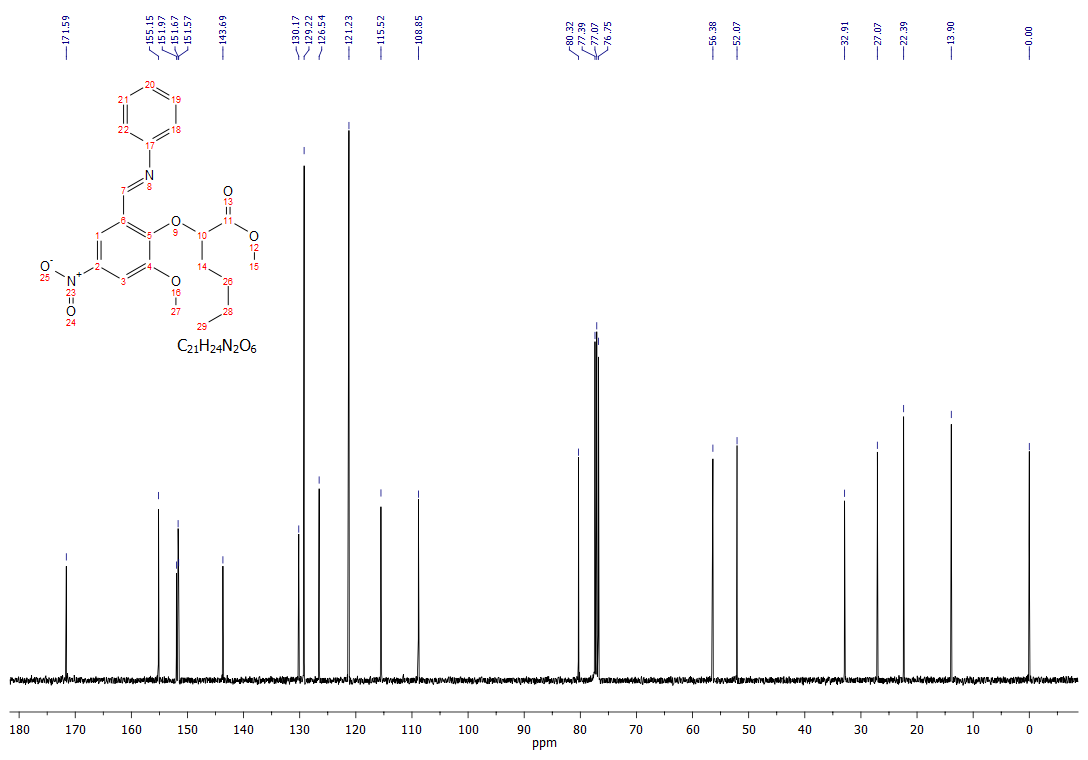** |

|  | **GC-MS** |
| --- | --- |
|  | **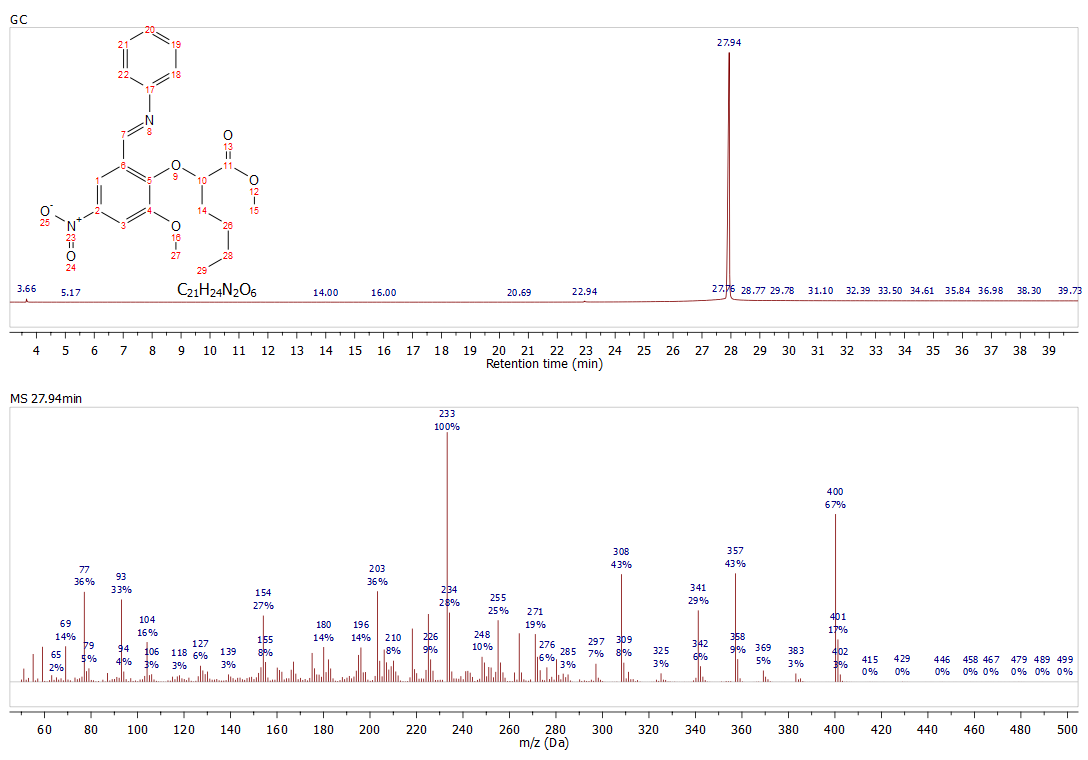** |

|  | **FTIR** |
| --- | --- |
|  | **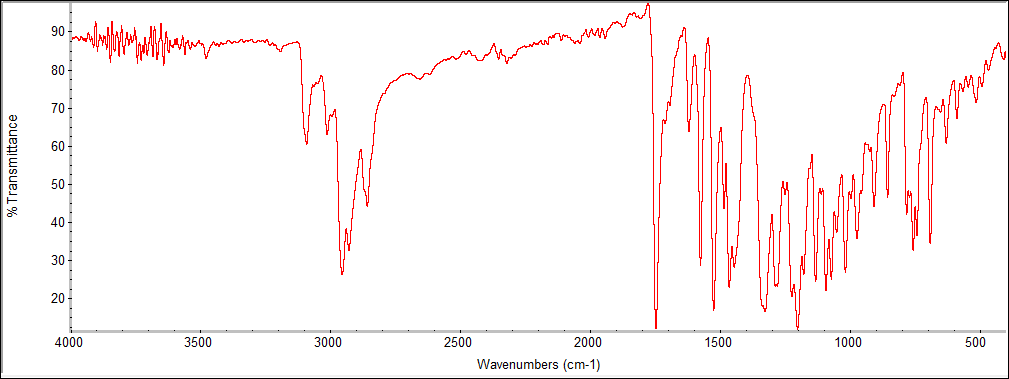** |

| **3g** | **Ethyl 2-(2-methoxy-4-nitro-6-((phenylimino)methyl)phenoxy)acetate** |
| --- | --- |
|  | **^1^H NMR** |
|  | **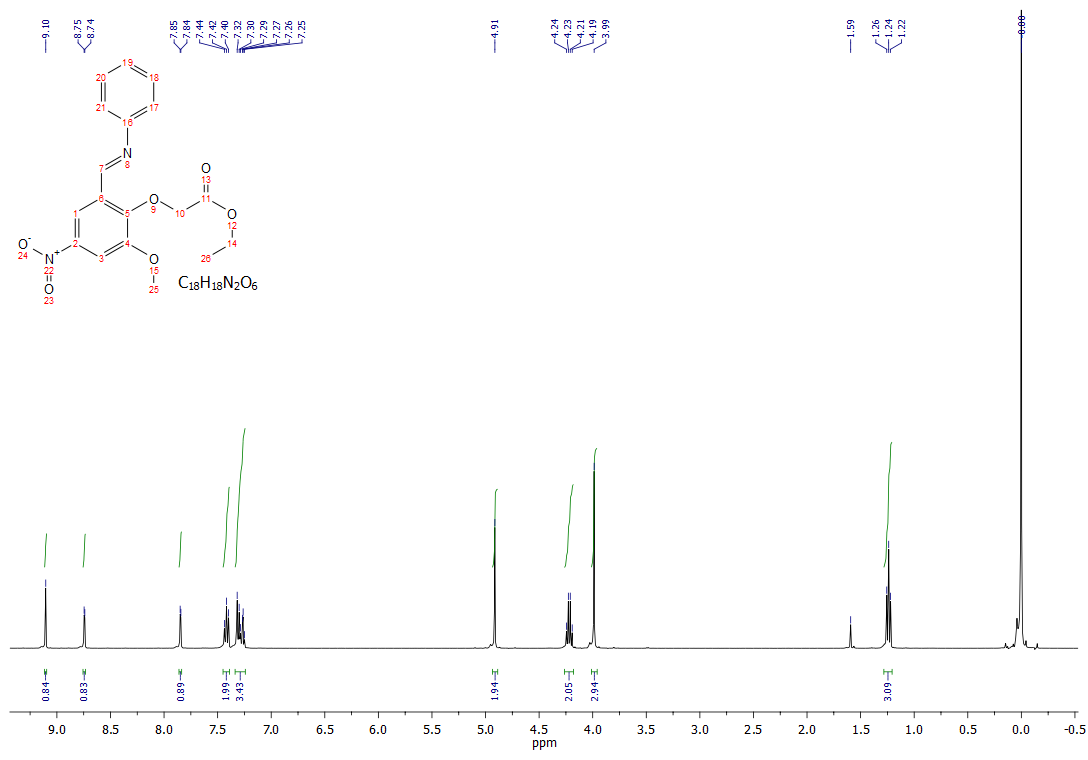** |

|  | **^13^C NMR** |
| --- | --- |
|  | **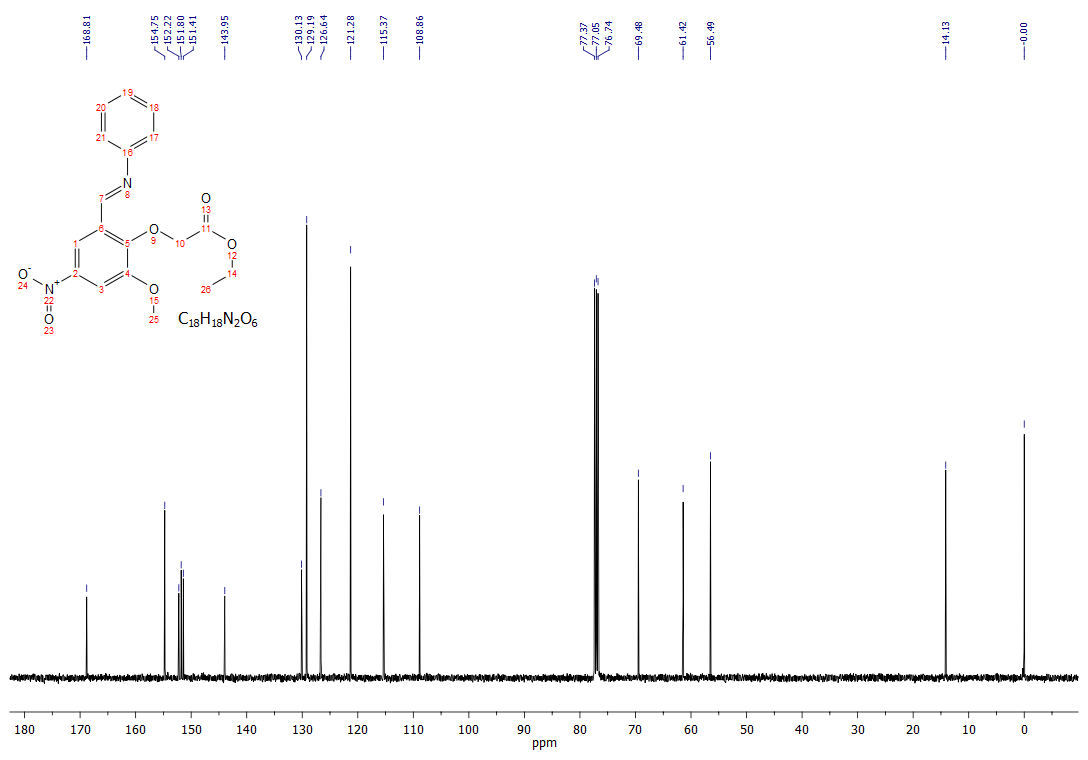** |

|  | **GC-MS** |
| --- | --- |
|  | **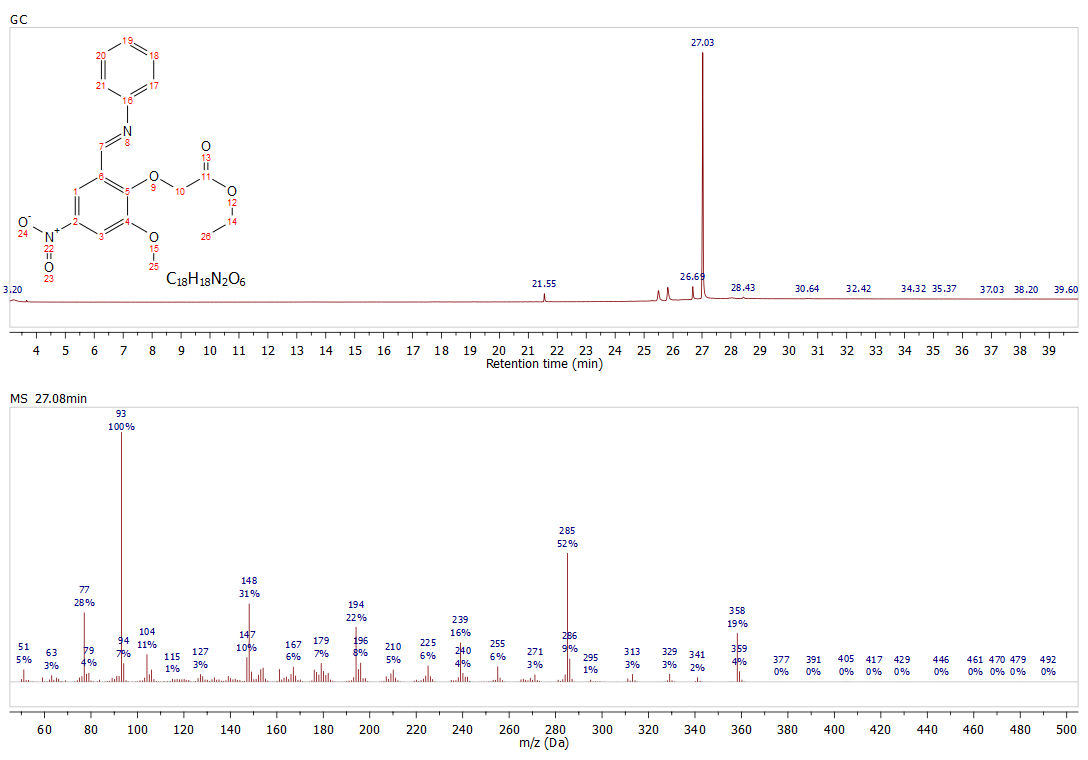** |

|  | **FTIR** |
| --- | --- |
|  | **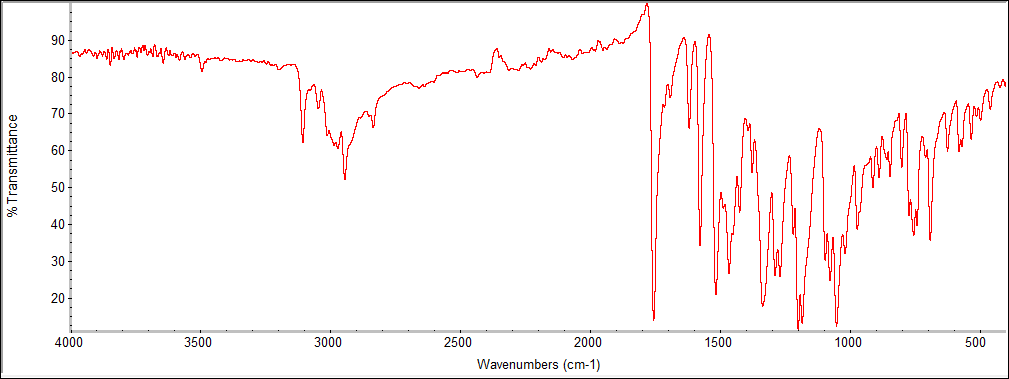** |

| **3h** | **Methyl 2-(2-((4-methoxyphenylimino)methyl)-4-nitrophenoxy)butanoate** |
| --- | --- |
|  | **^1^H NMR** |
|  | **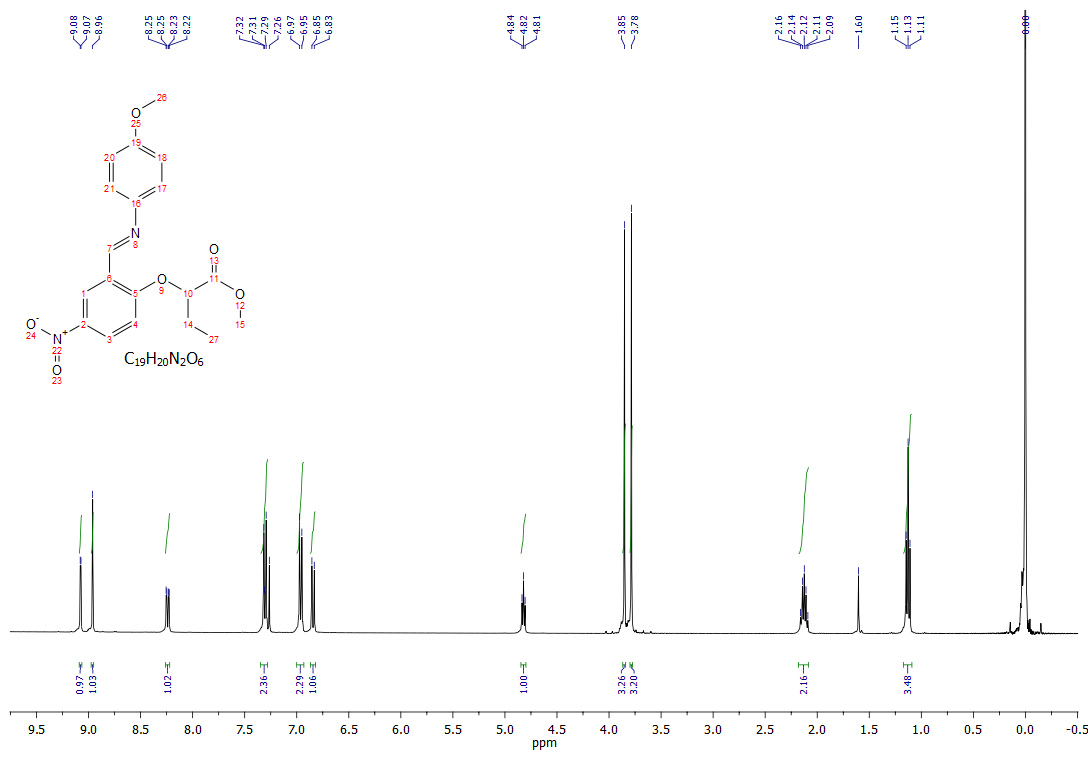** |

|  | **^13^C NMR** |
| --- | --- |
|  | **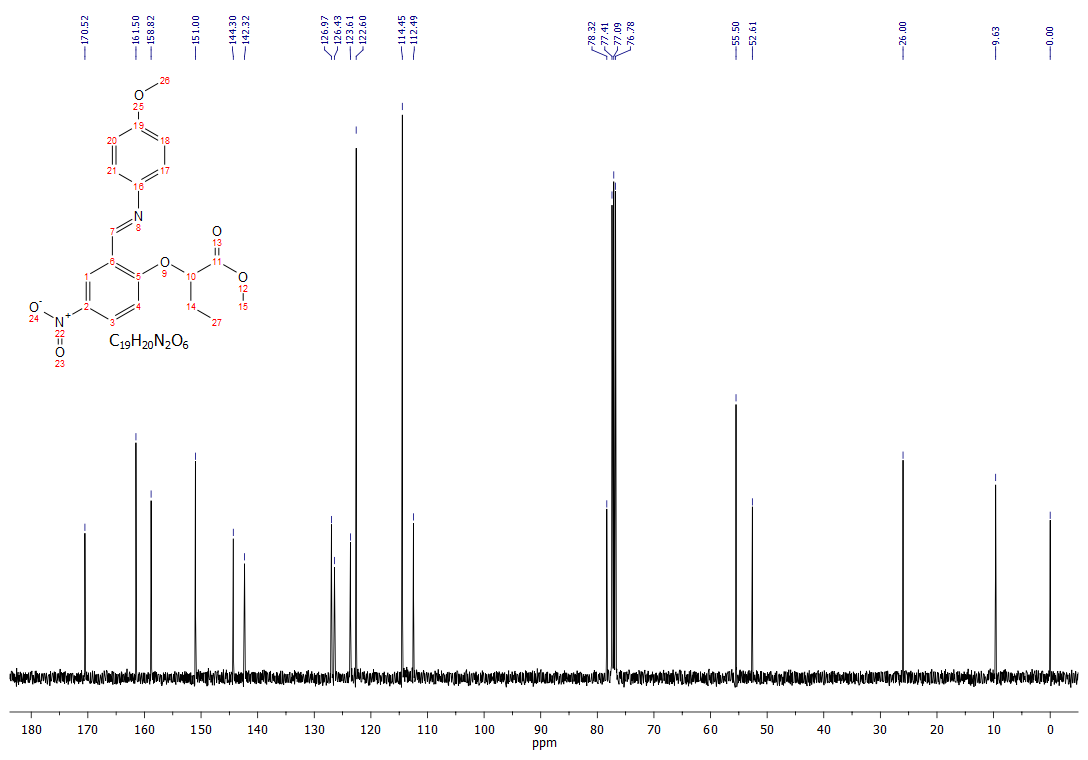** |

|  | **GC-MS** |
| --- | --- |
|  | **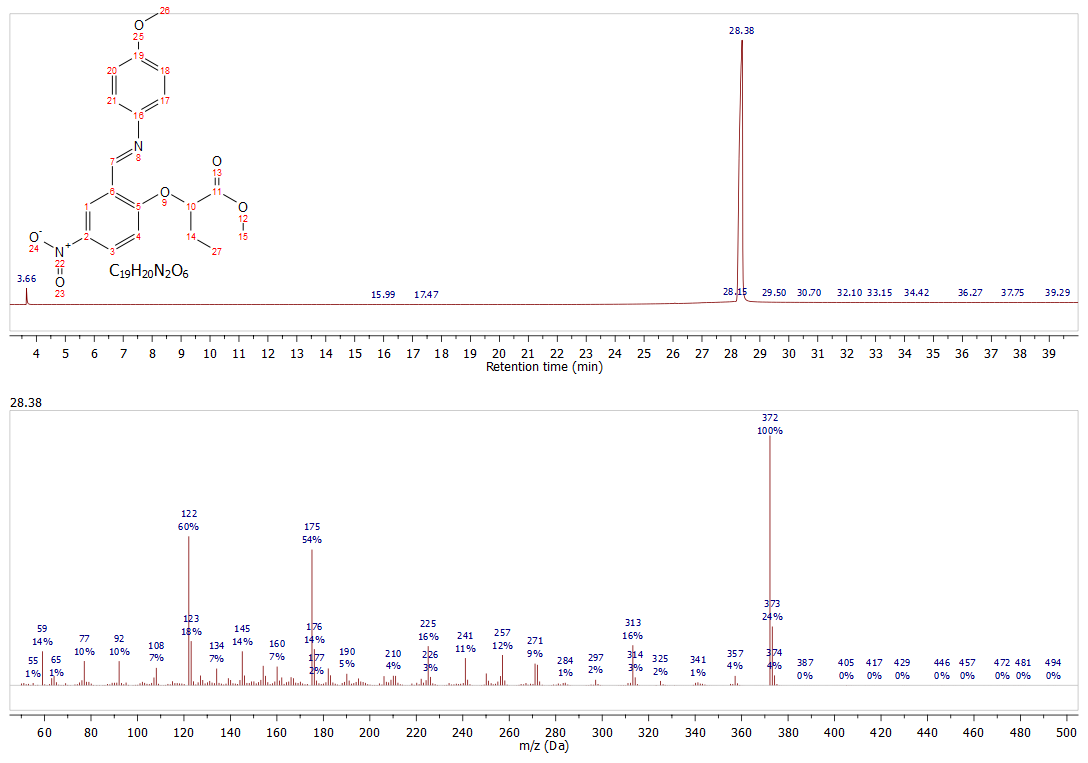** |

|  | **FTIR** |
| --- | --- |
|  | **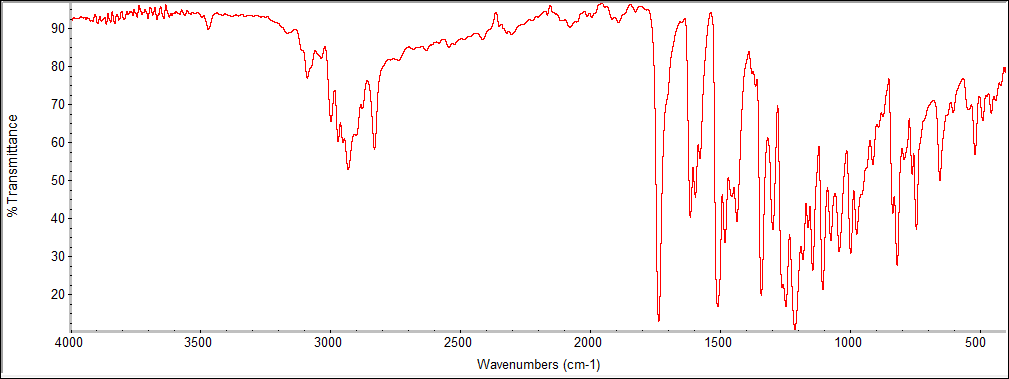** |

| **3i** | **Methyl 2-(2-((4-metoxyphenylimino)methyl)-4-nitrophenoxy)hexanoate** |
| --- | --- |
|  | **^1^H NMR** |
|  | **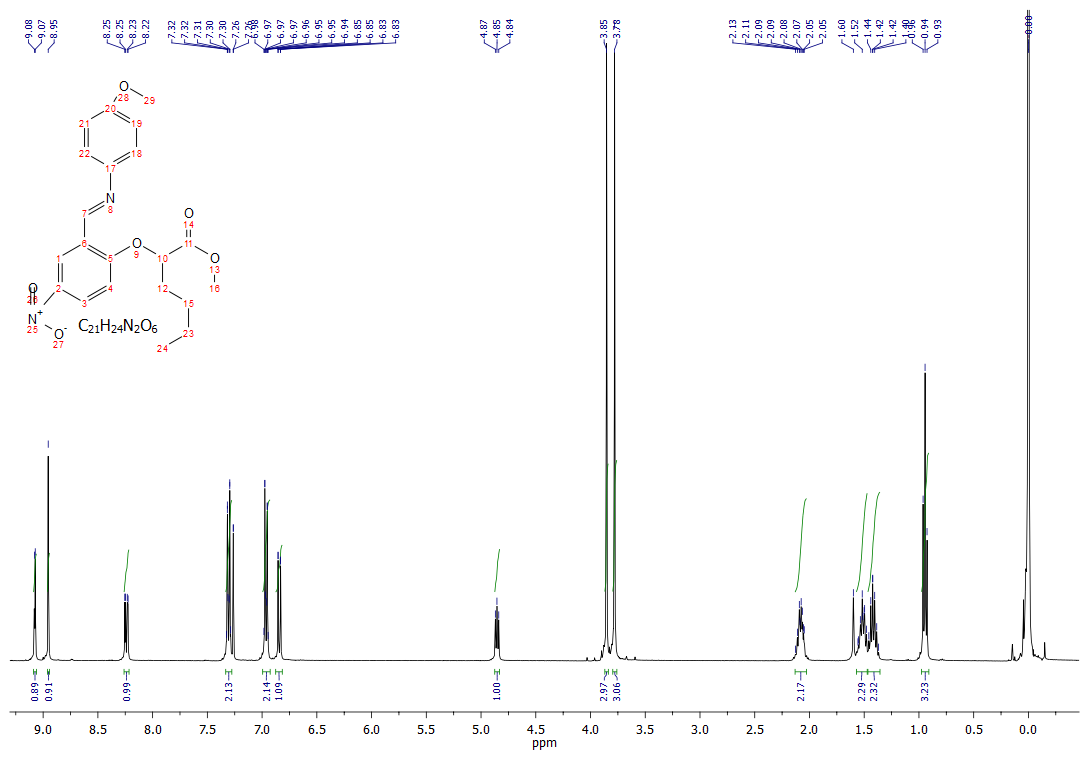** |

|  | **^13^C NMR** |
| --- | --- |
|  | **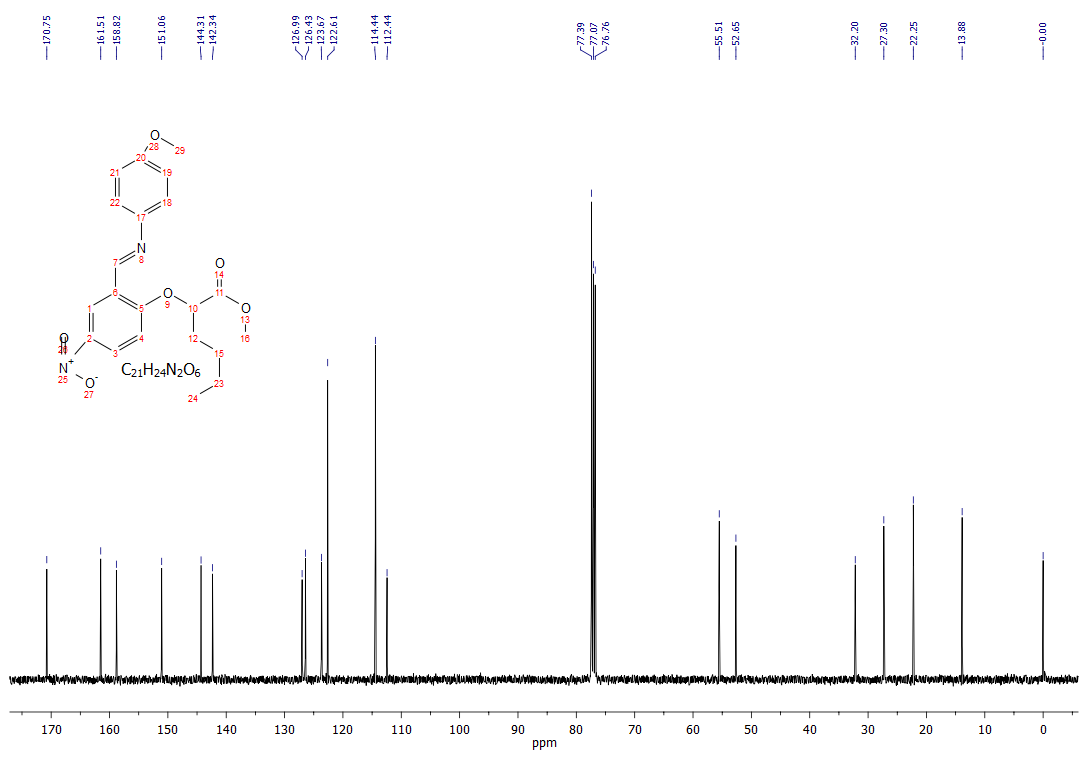** |

|  | **GC-MS** |
| --- | --- |
|  | **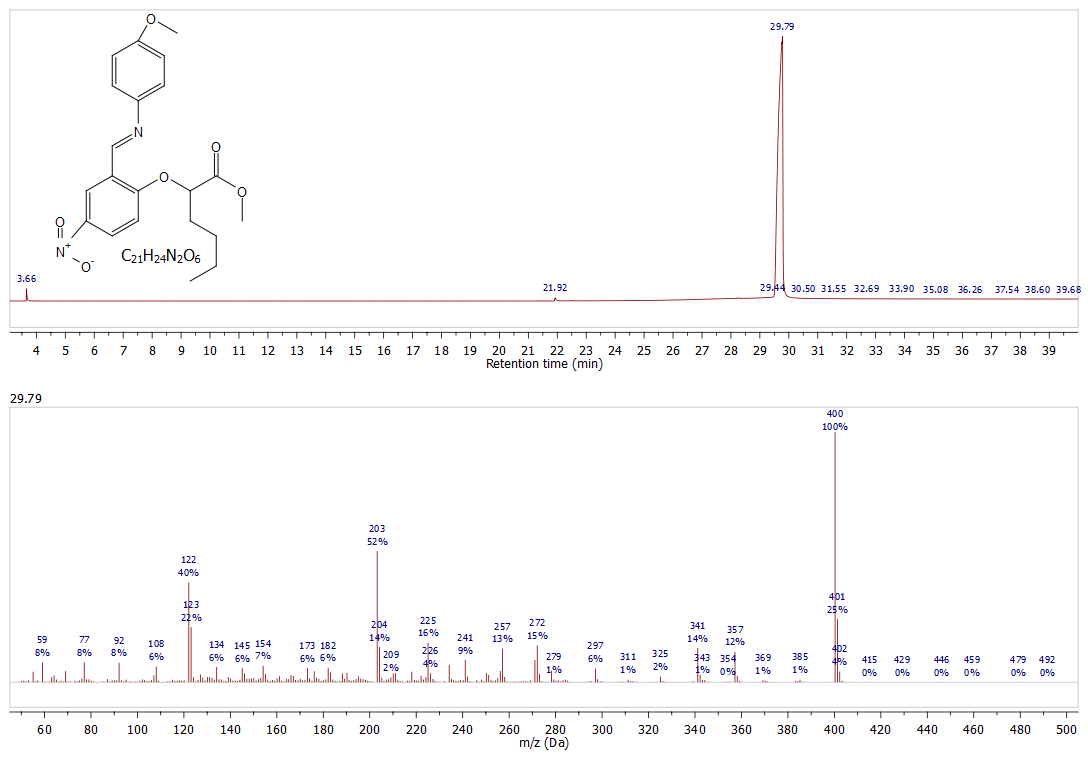** |

|  | **FTIR** |
| --- | --- |
|  | **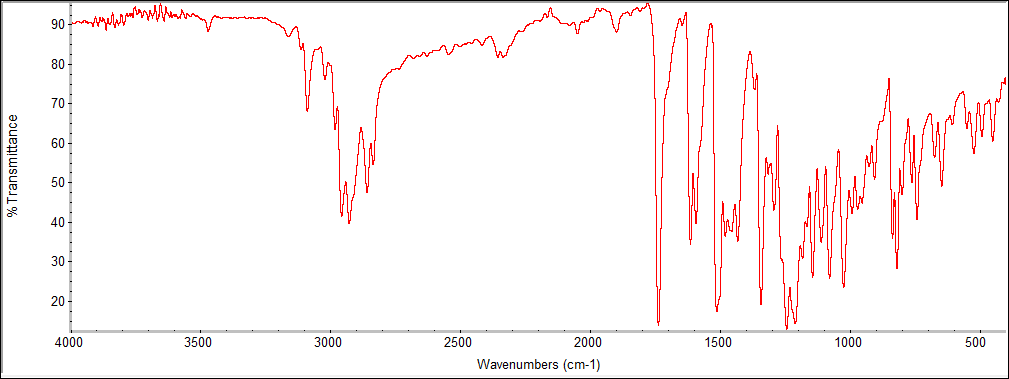** |

| **3j** | **Methyl 2-(2-((4-methoxyphenylimino)methyl)-6-methoxy-4-nitrophenoxy)butanoate** |
| --- | --- |
|  | **^1^H NMR** |
|  | **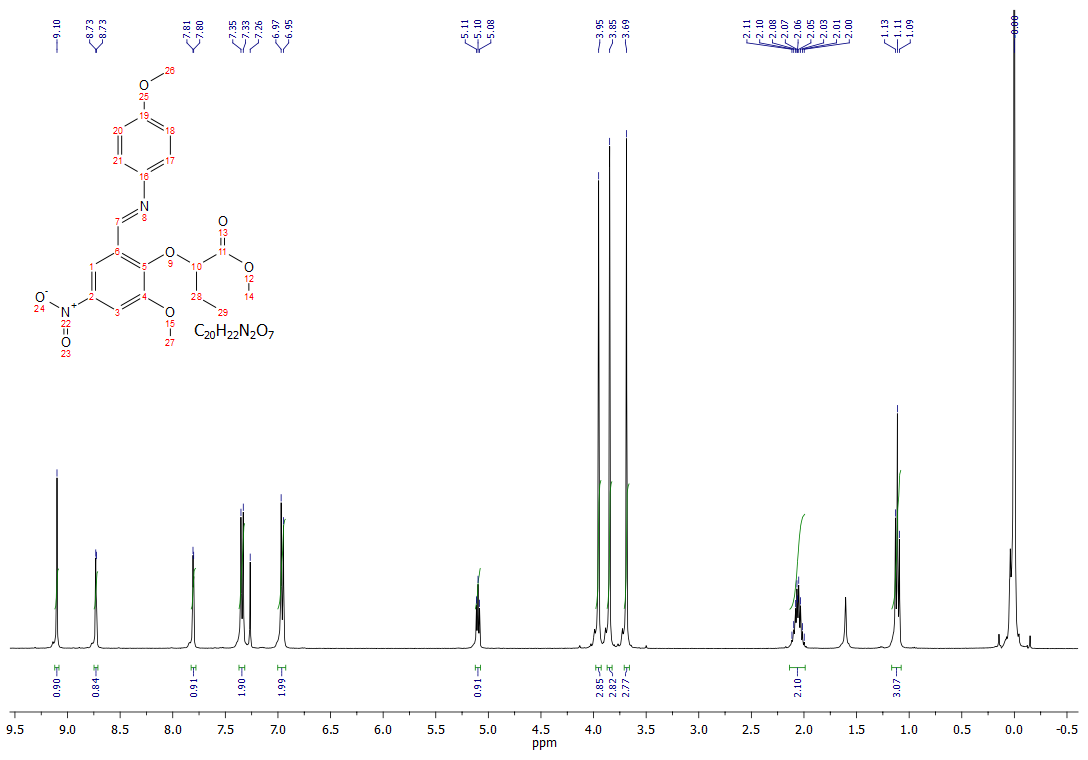** |

|  | **^13^C NMR** |
| --- | --- |
|  | **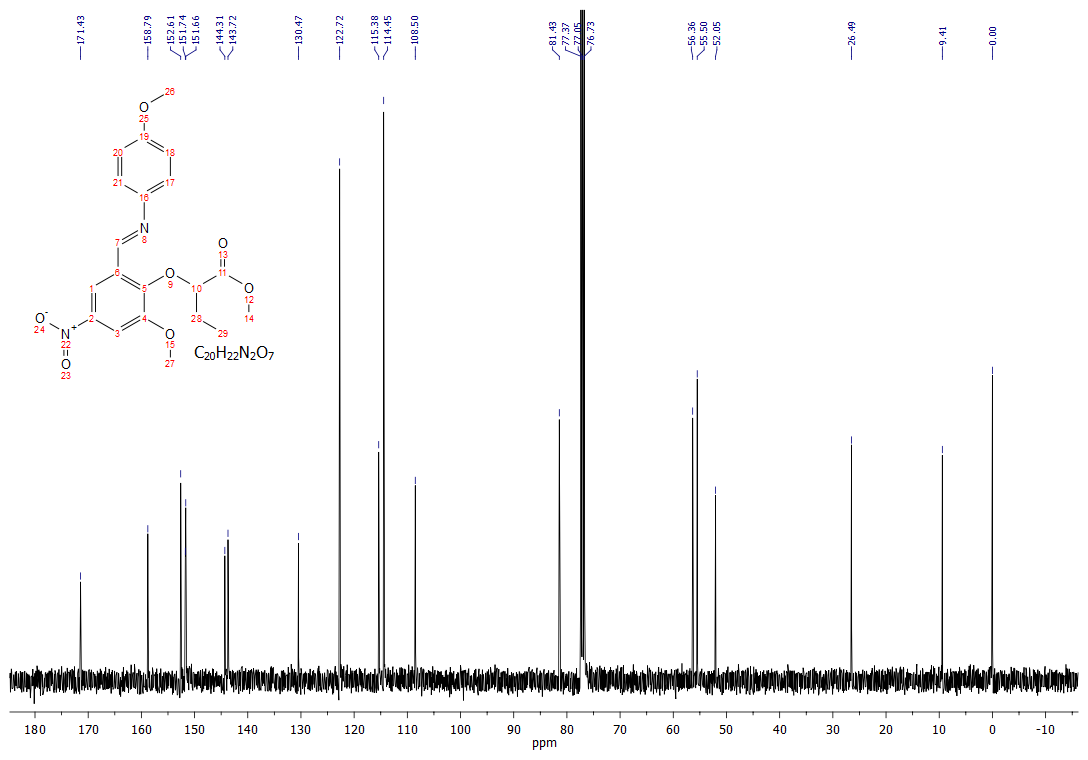** |

|  | **GC-MS** |
| --- | --- |
|  | **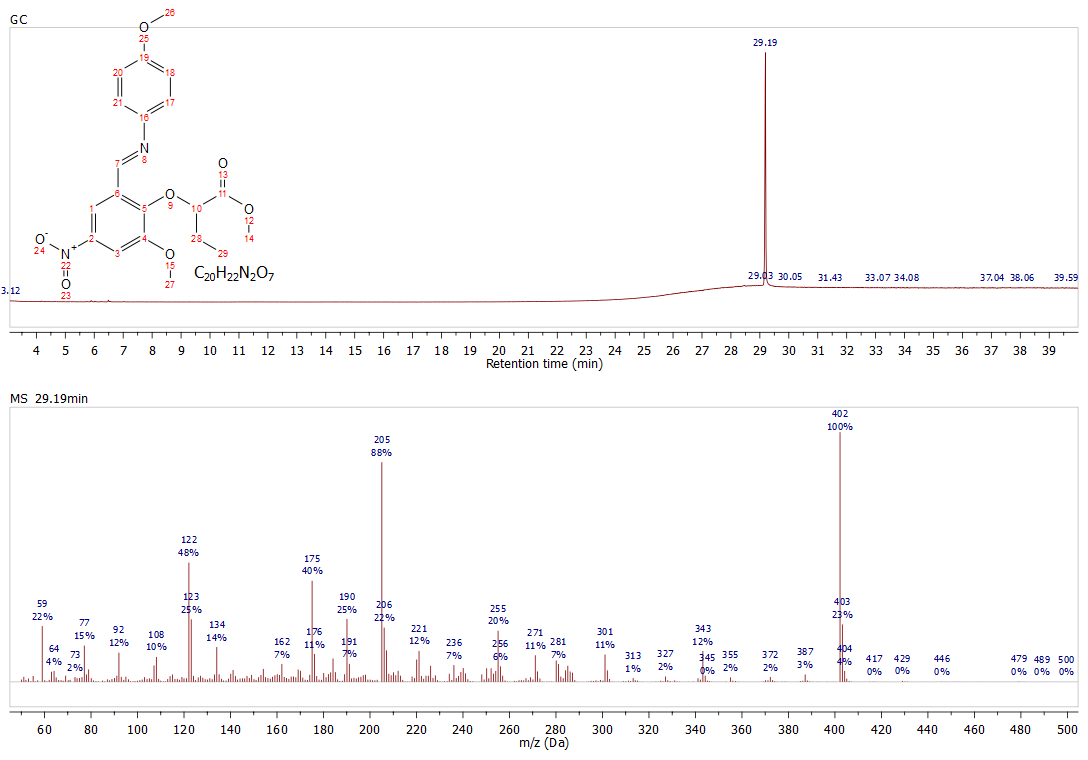** |

|  | **FTIR** |
| --- | --- |
|  | **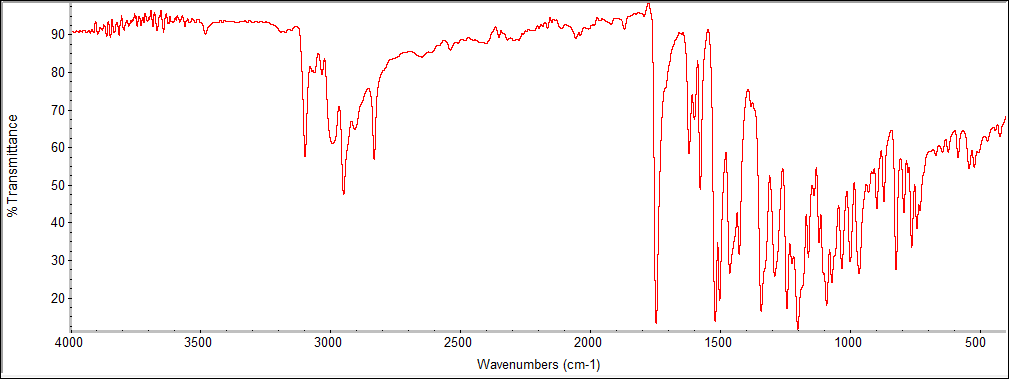** |

| **3k** | **Methyl 2-(2-((4-methoxyphenylimino)methyl)-6-methoxy-4-nitrophenoxy)pentanoate** |
| --- | --- |
|  | **^1^H NMR** |
|  | **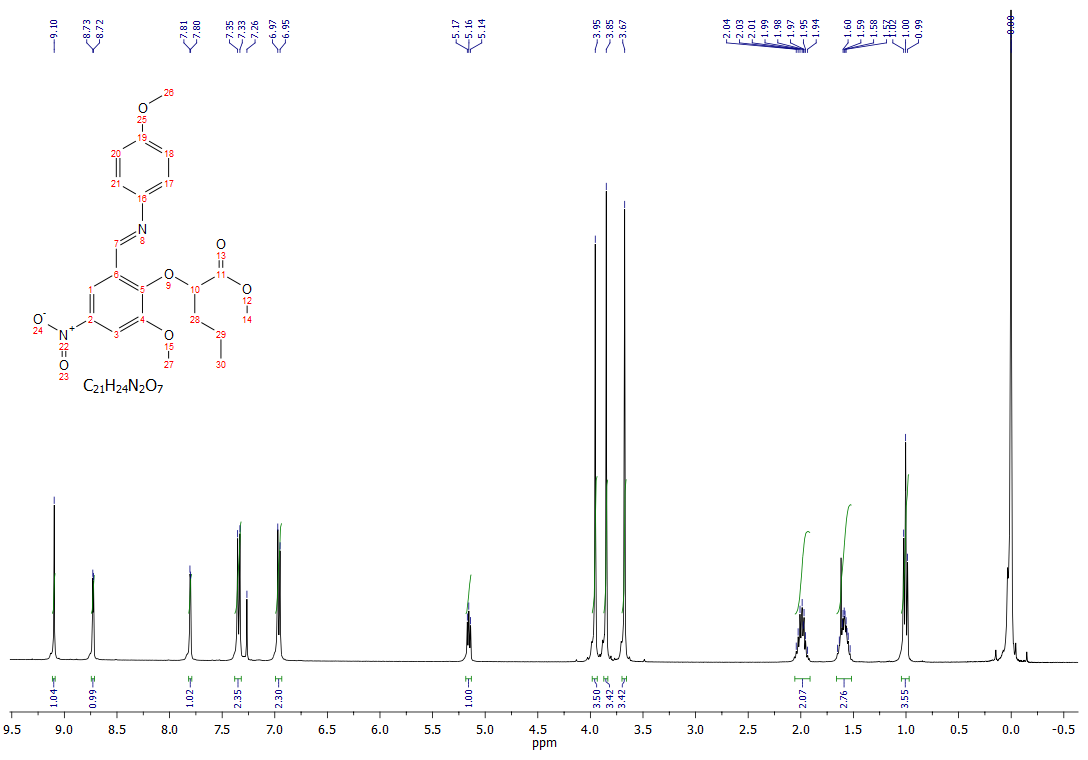** |

|  | **^13^C NMR** |
| --- | --- |
|  | **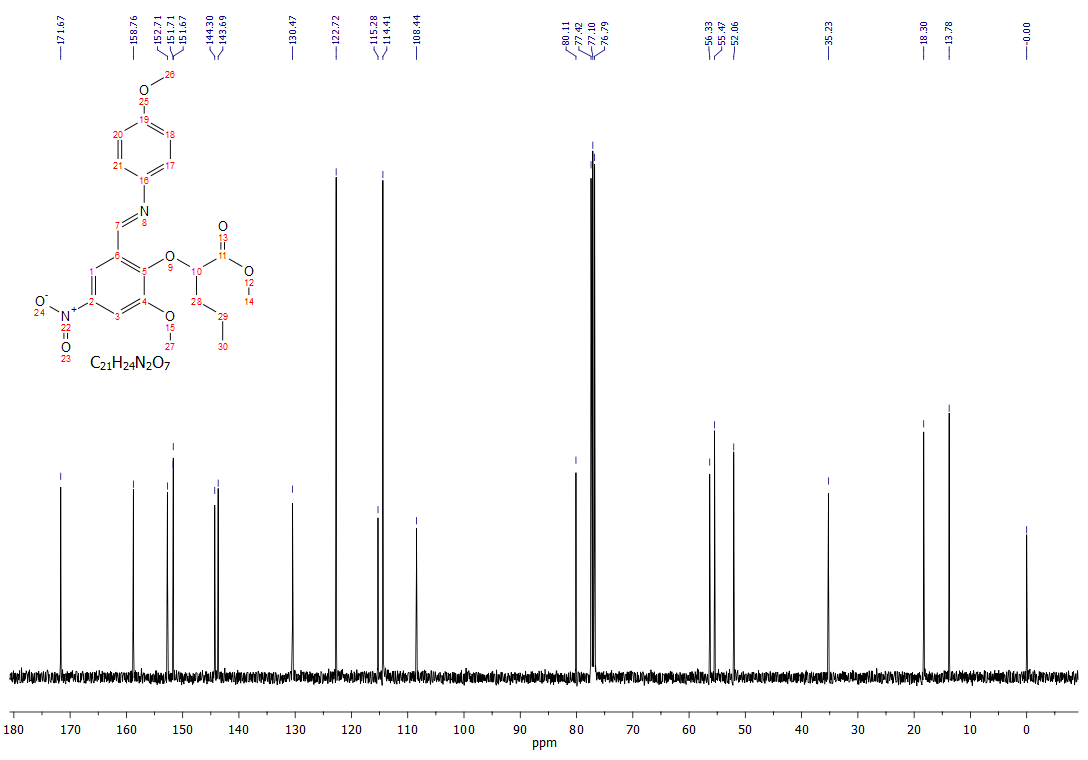** |

|  | **GC-MS** |
| --- | --- |
|  | **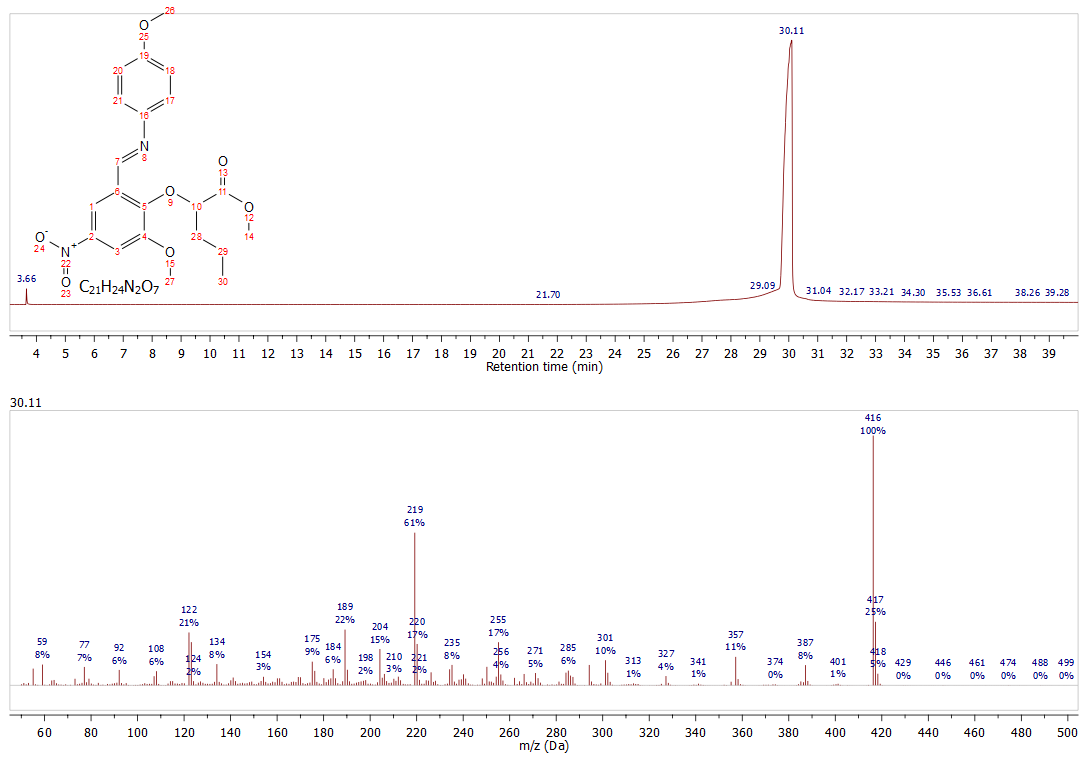** |

|  | **FTIR** |
| --- | --- |
|  | **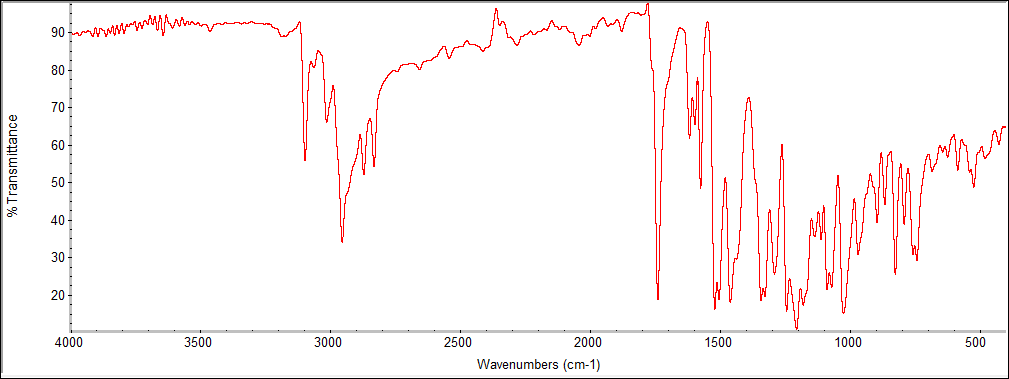** |

| **3l** | **Methyl 2-(2-((4-methoxyphenylimino)methyl)-6-methoxy-4-nitrophenoxy)hexanoate** |
| --- | --- |
|  | **^1^H NMR** |
|  | **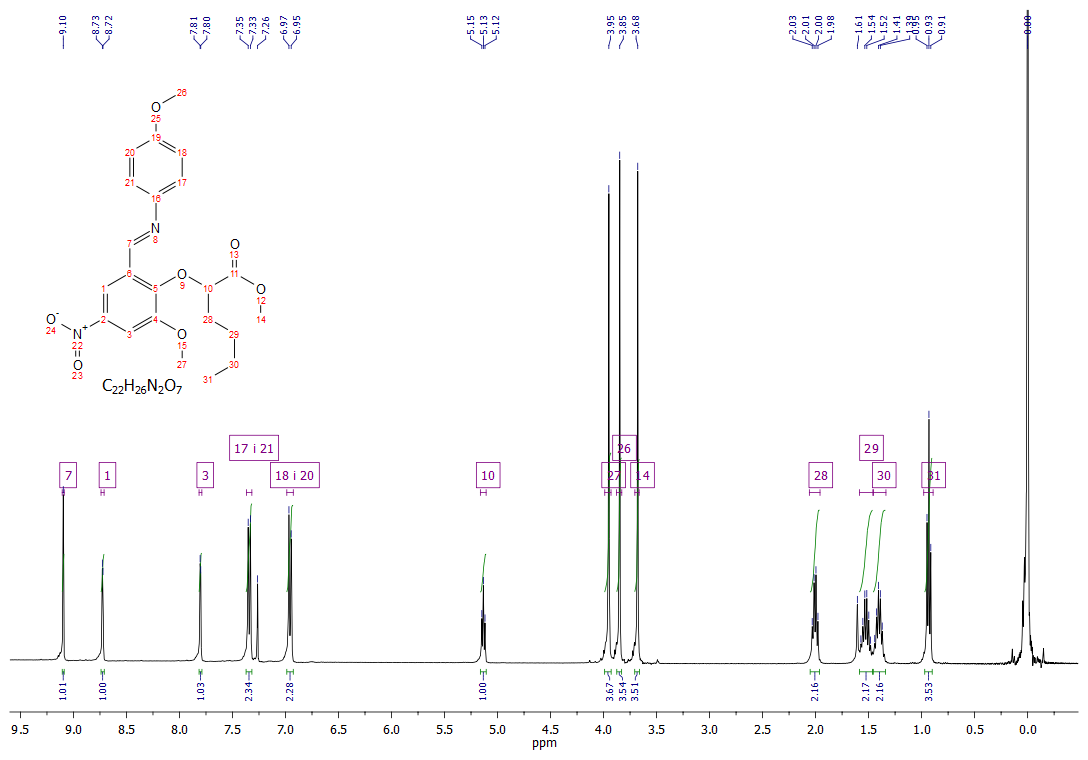** |
|  | **^13^C NMR** |
|  | **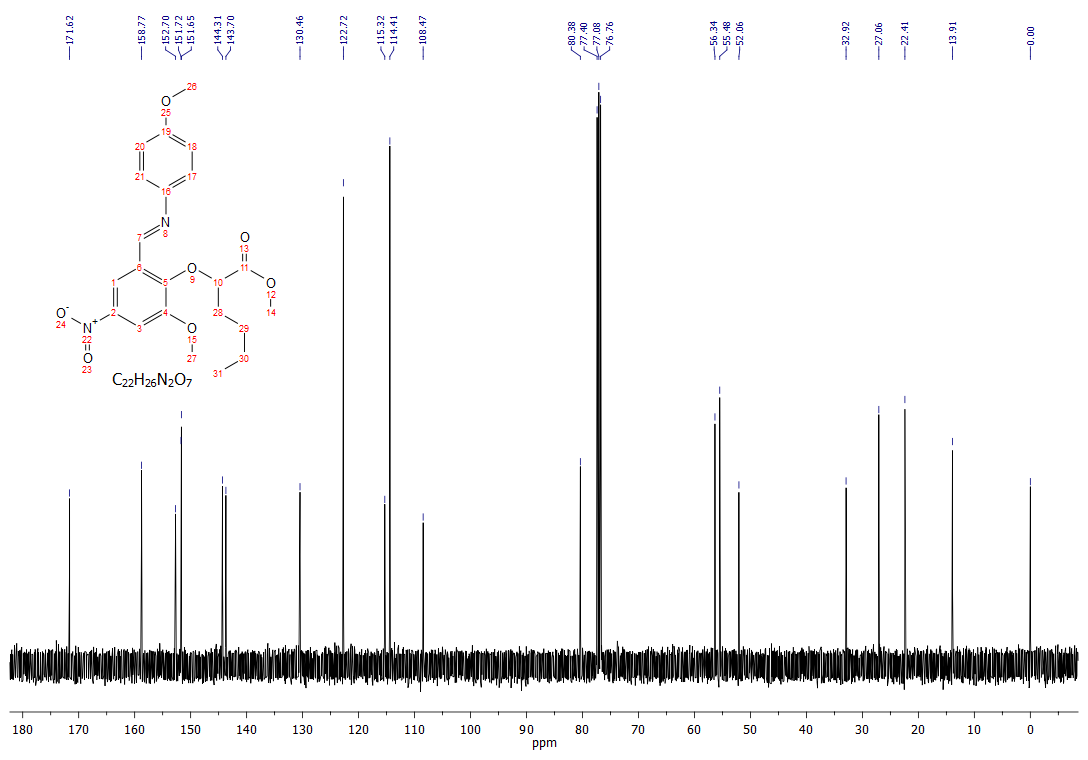** |

|  | **GC-MS** |
| --- | --- |
|  | **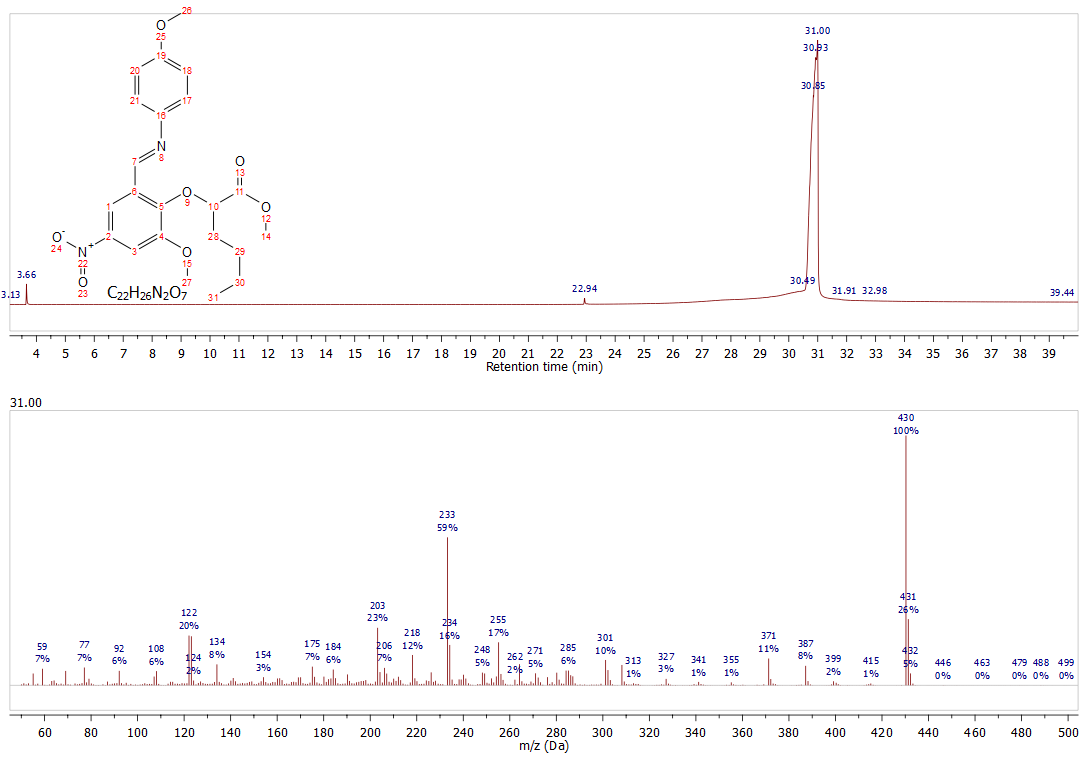** |

|  | **FTIR** |
| --- | --- |
|  | **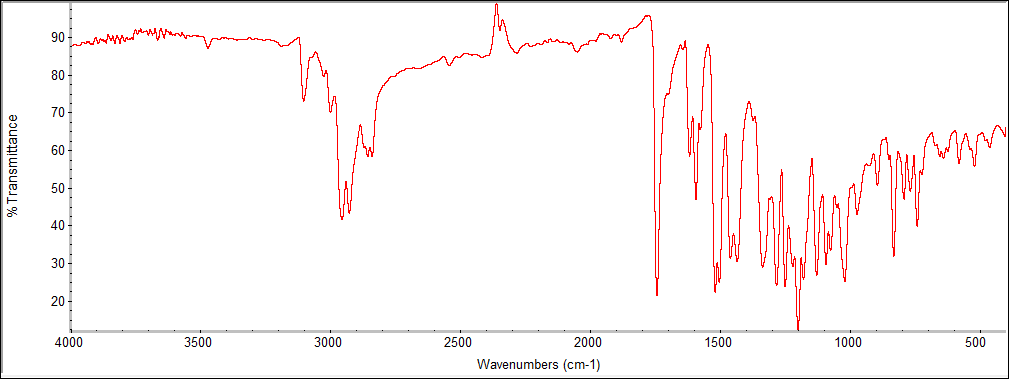** |

| **4a** | **Methyl 2-(4-nitro-2-((phenylamino)methyl)phenoxy)butanoate** |
| --- | --- |
|  | **^1^H NMR** |
|  | **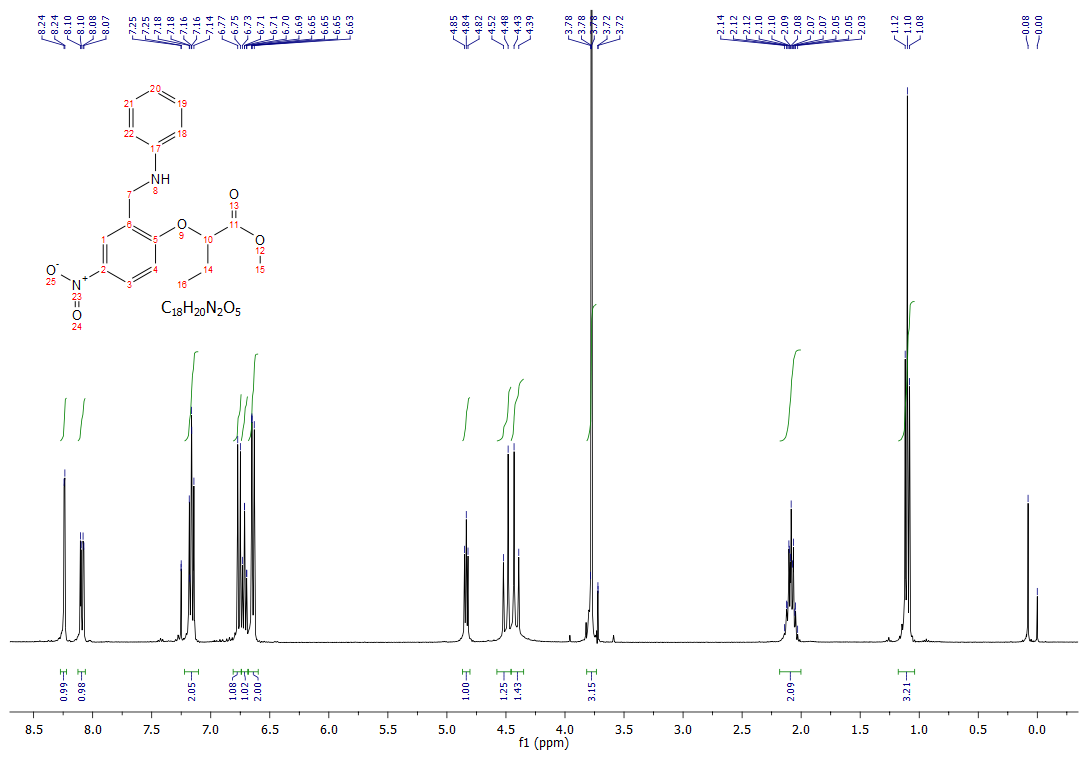** |

**+ D_2_O**


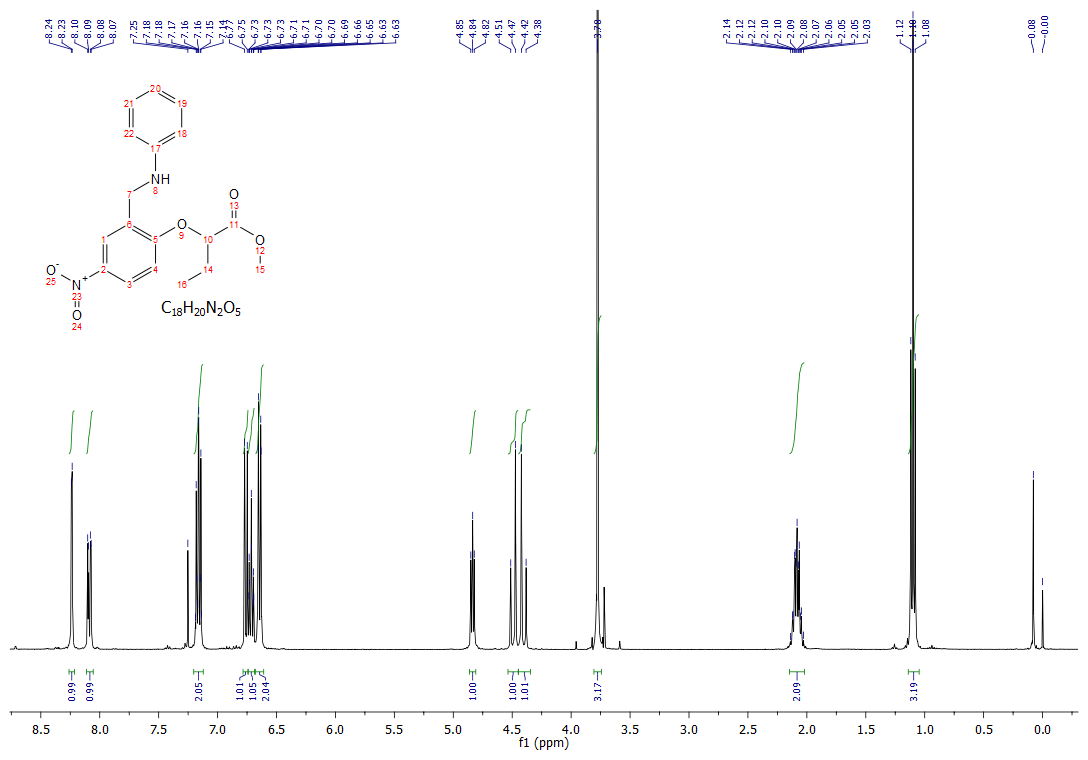


|  | **^13^C NMR** |
| --- | --- |
|  | **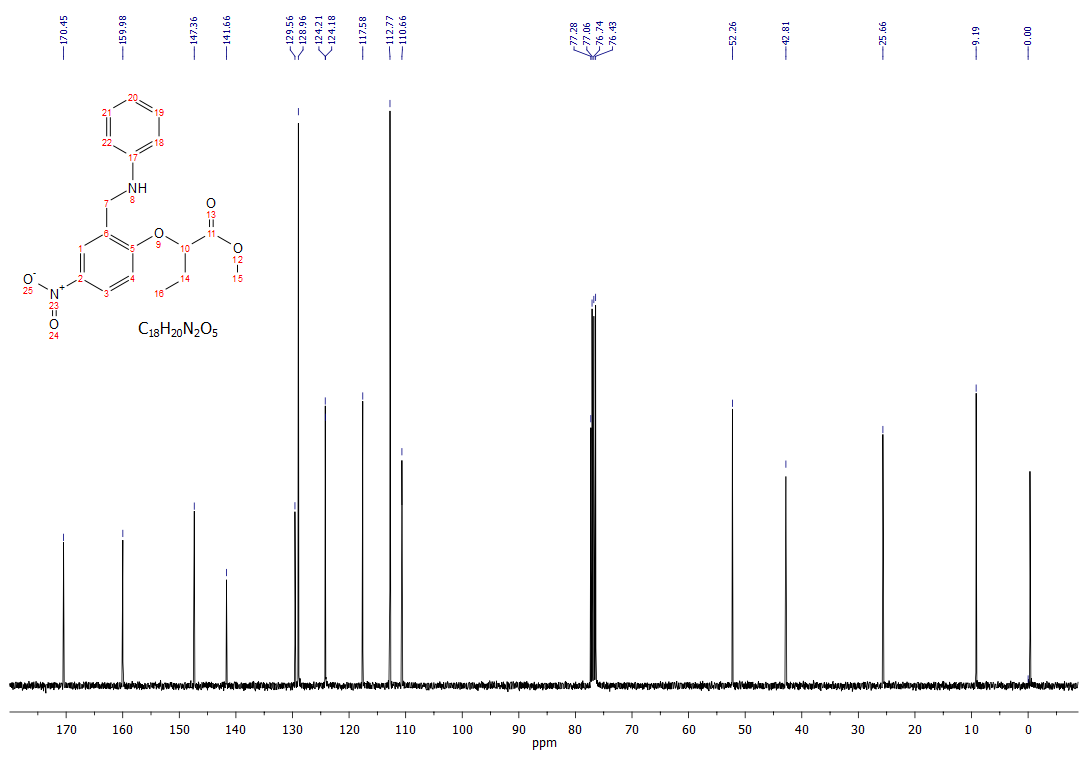** |

|  | **GC-MS** |
| --- | --- |
|  | **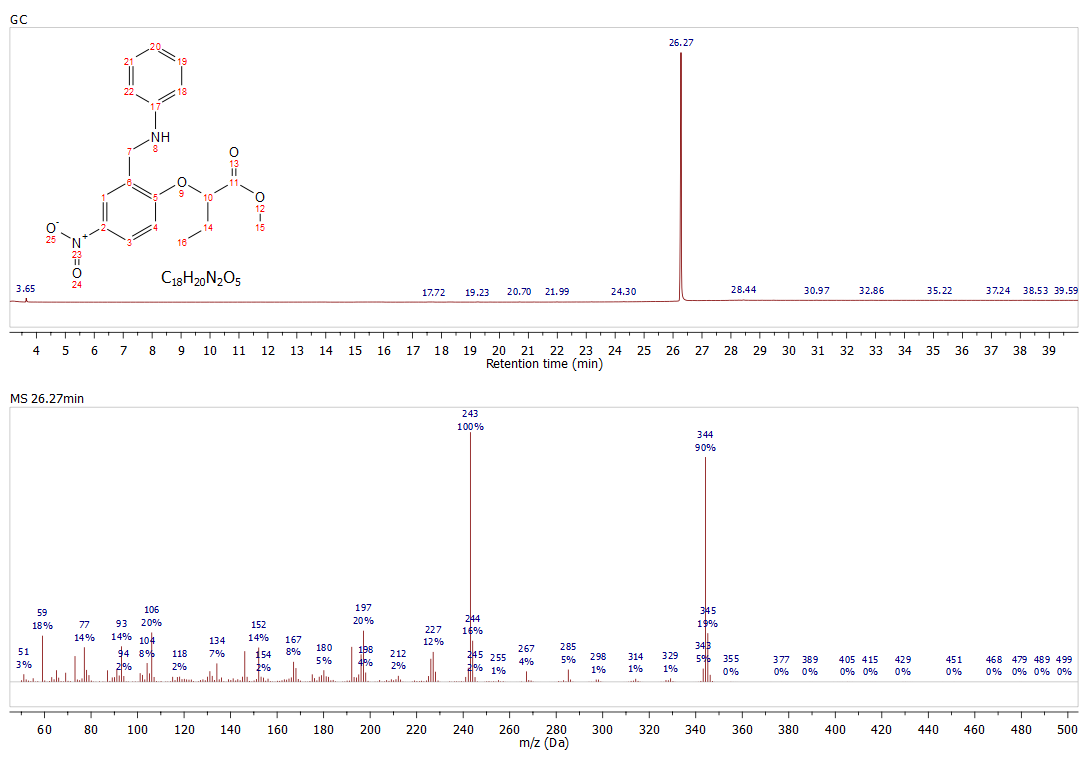** |

|  | **FTIR** |
| --- | --- |
|  | **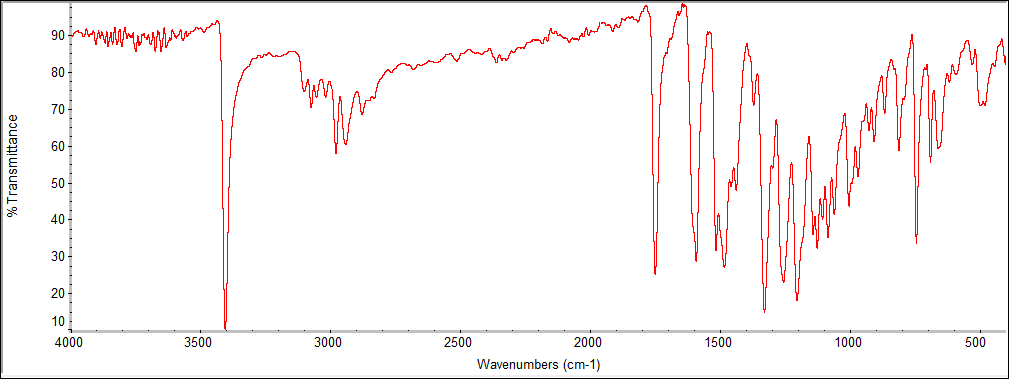** |

| **4b** | **Methyl 2-(4-nitro-2-((phenylamino)methyl)phenoxy)pentanoate** |
| --- | --- |
|  | **^1^H NMR** |
|  | **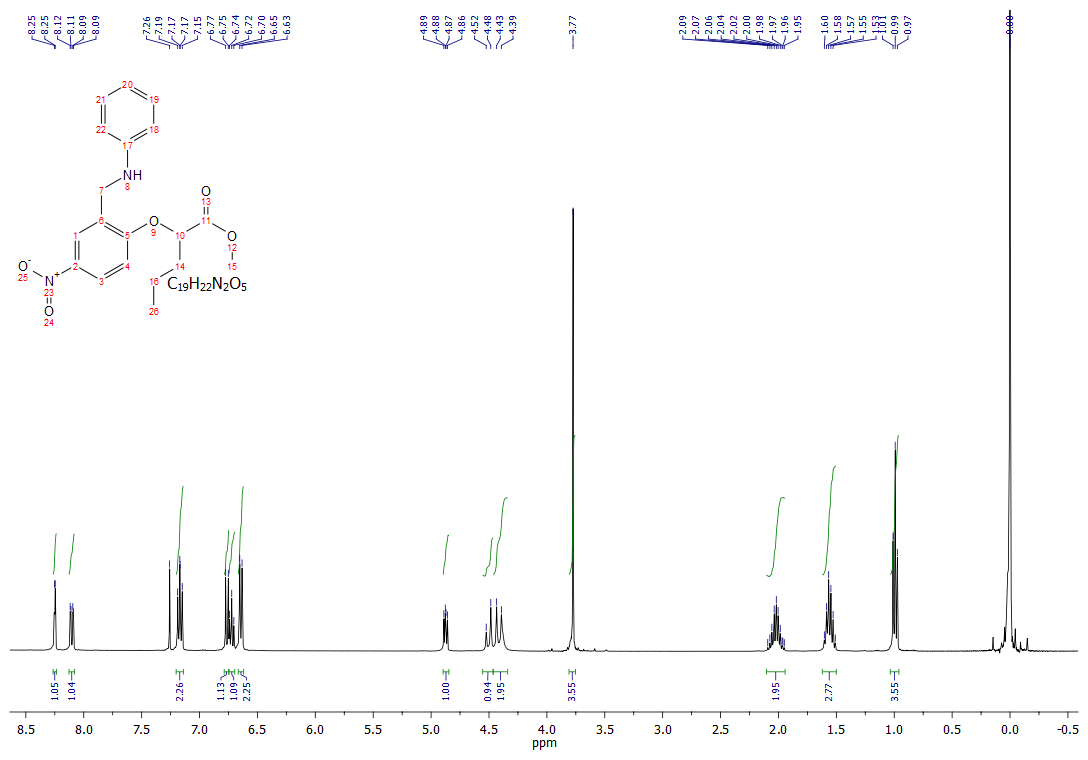** |

|  | **^13^C NMR** |
| --- | --- |
|  | **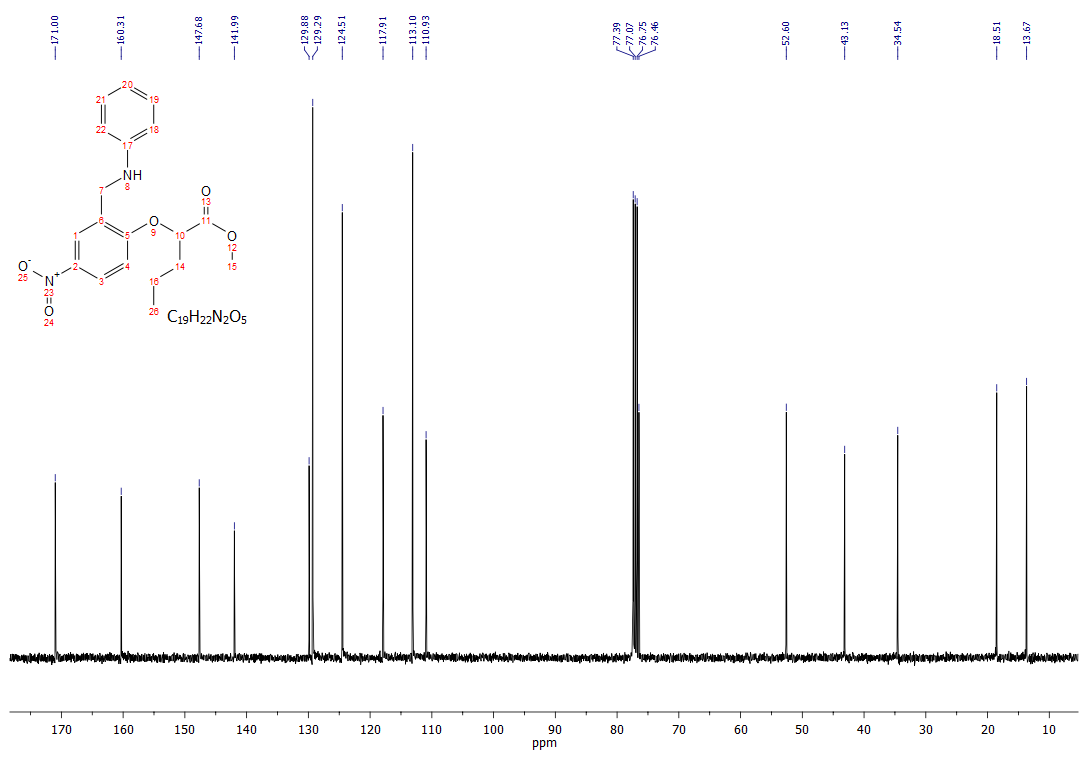** |

|  | **GC-MS** |
| --- | --- |
|  | **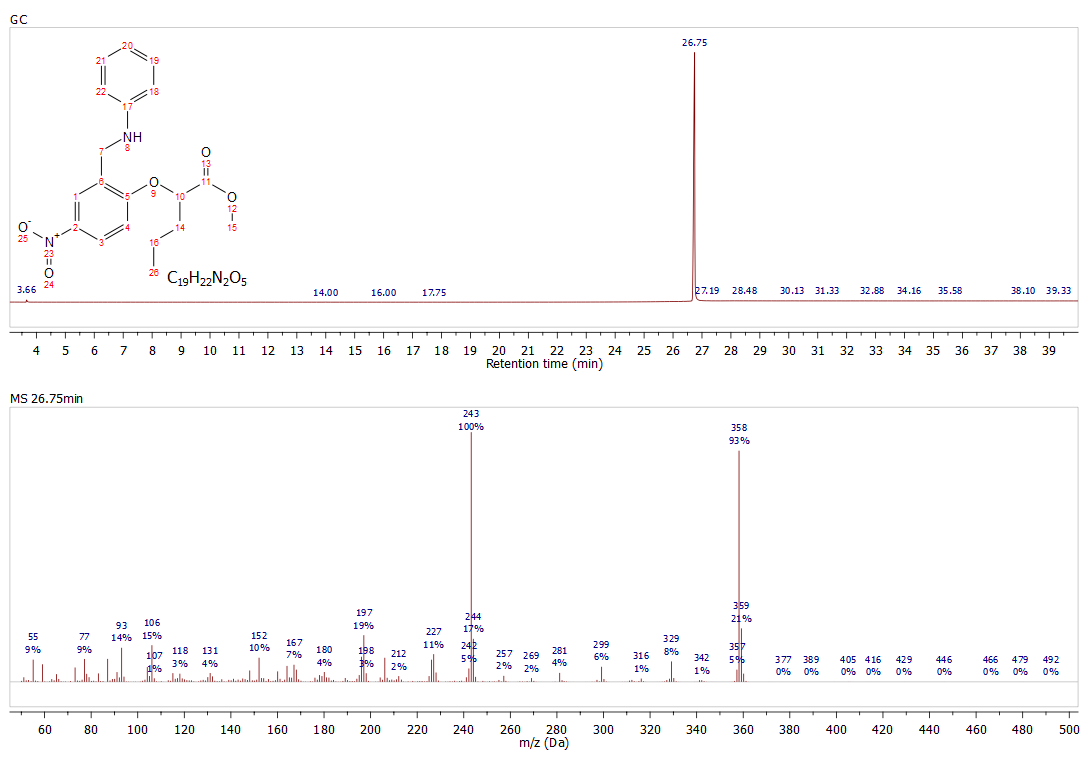** |

|  | **FTIR** |
| --- | --- |
|  | **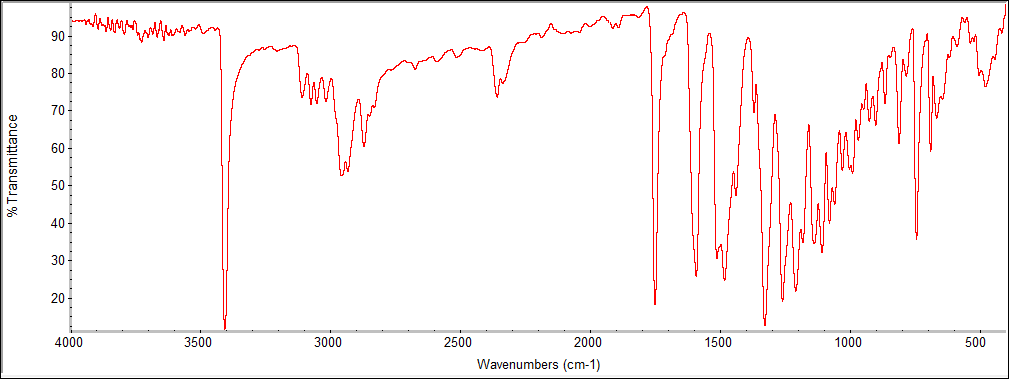** |

| **4c** | **Methyl 2-(4-nitro-2-((phenylamino)methyl)phenoxy)hexanoate** |
| --- | --- |
|  | **^1^H NMR** |
|  | **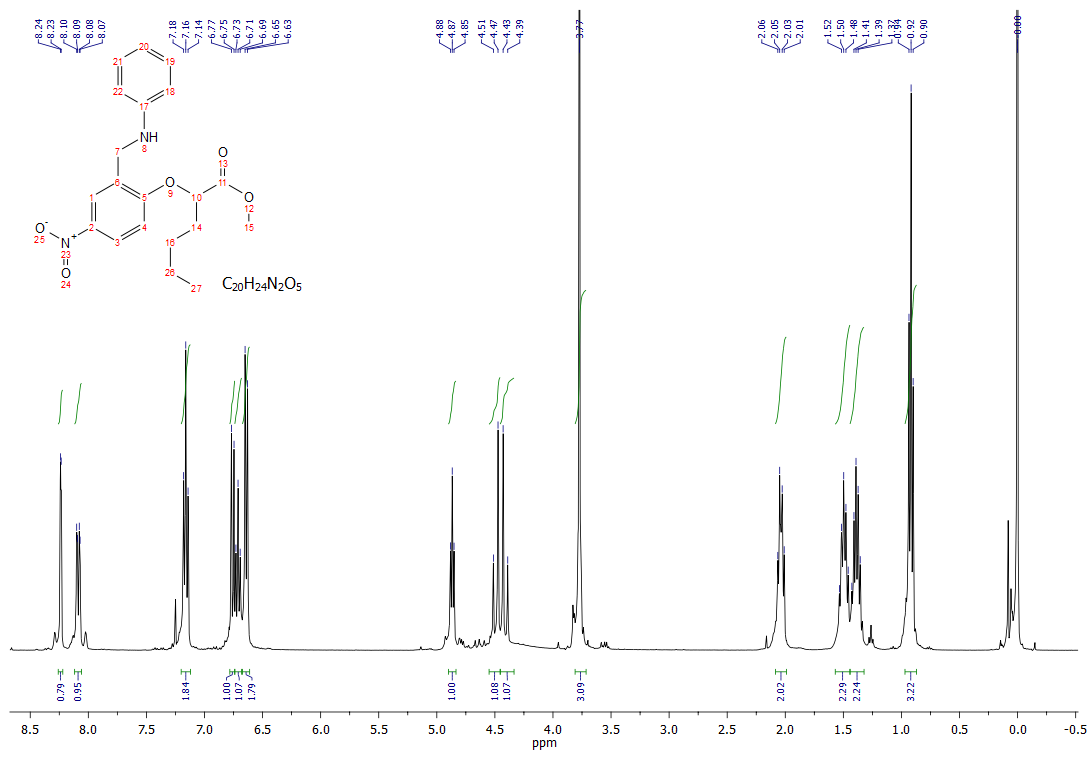** |

|  | **^13^C NMR** |
| --- | --- |
|  | **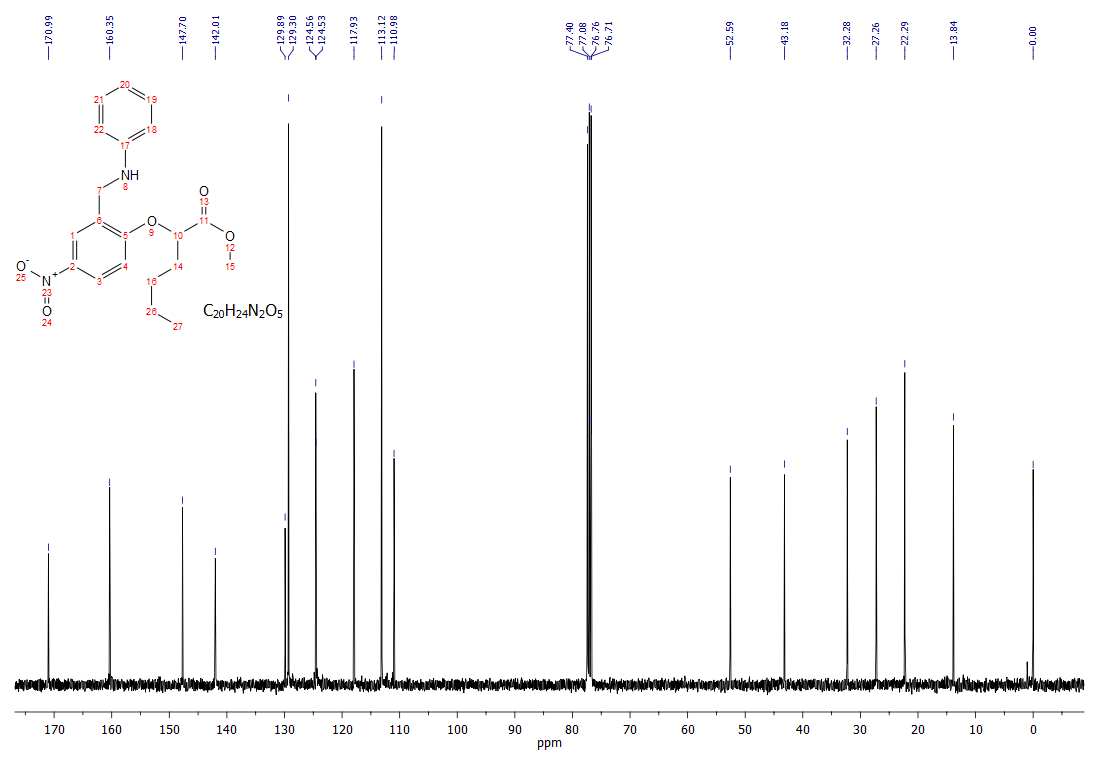** |

|  | **GC-MS** |
| --- | --- |
|  | **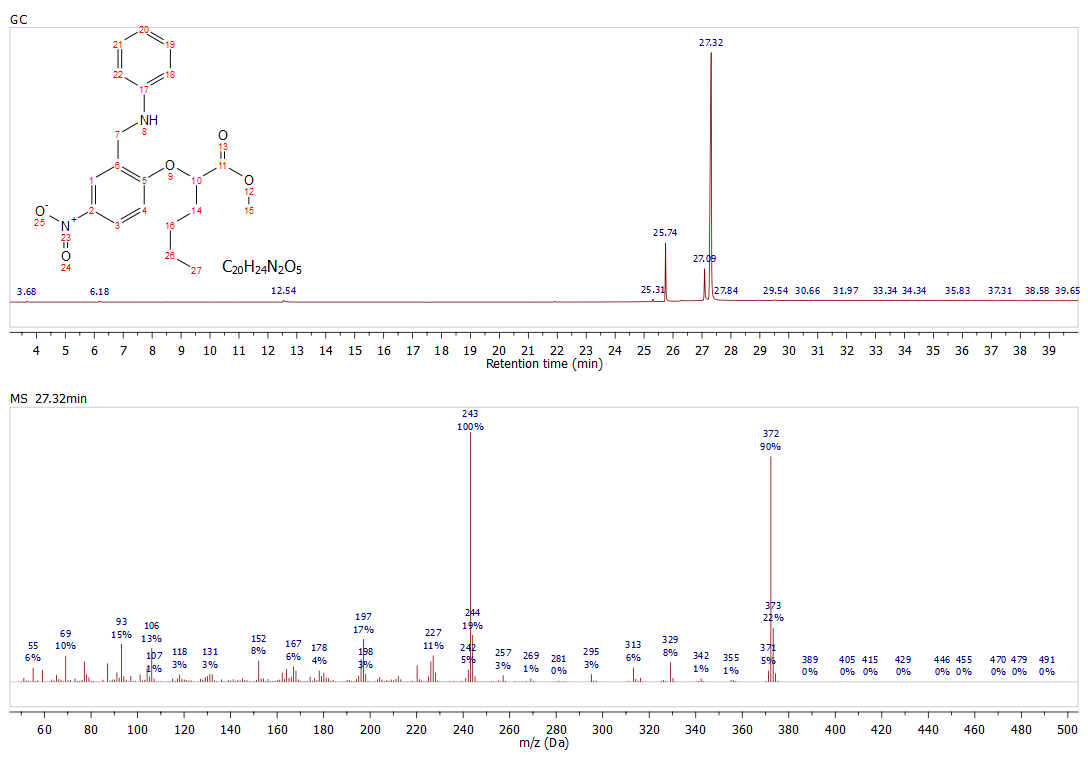** |

|  | **FTIR** |
| --- | --- |
|  | **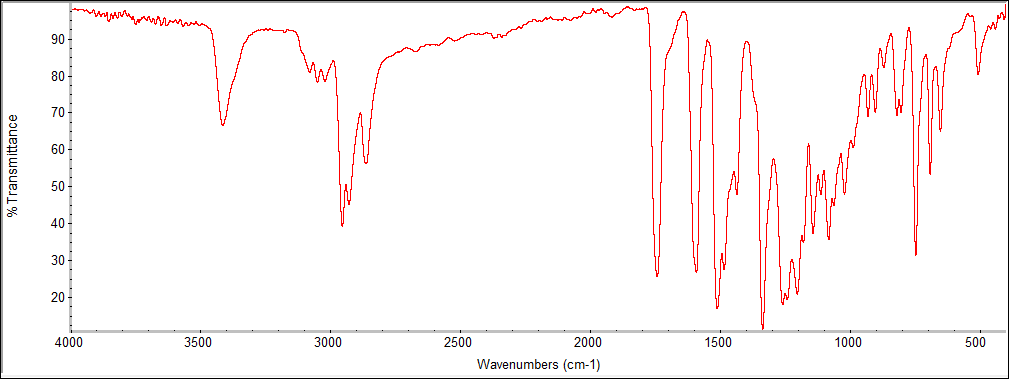** |

| **4d** | **Methyl 2-(2-methoxy-4-nitro-6-((phenylamino)methyl)phenoxy)butanoate** |
| --- | --- |
|  | **^1^H NMR** |
|  | **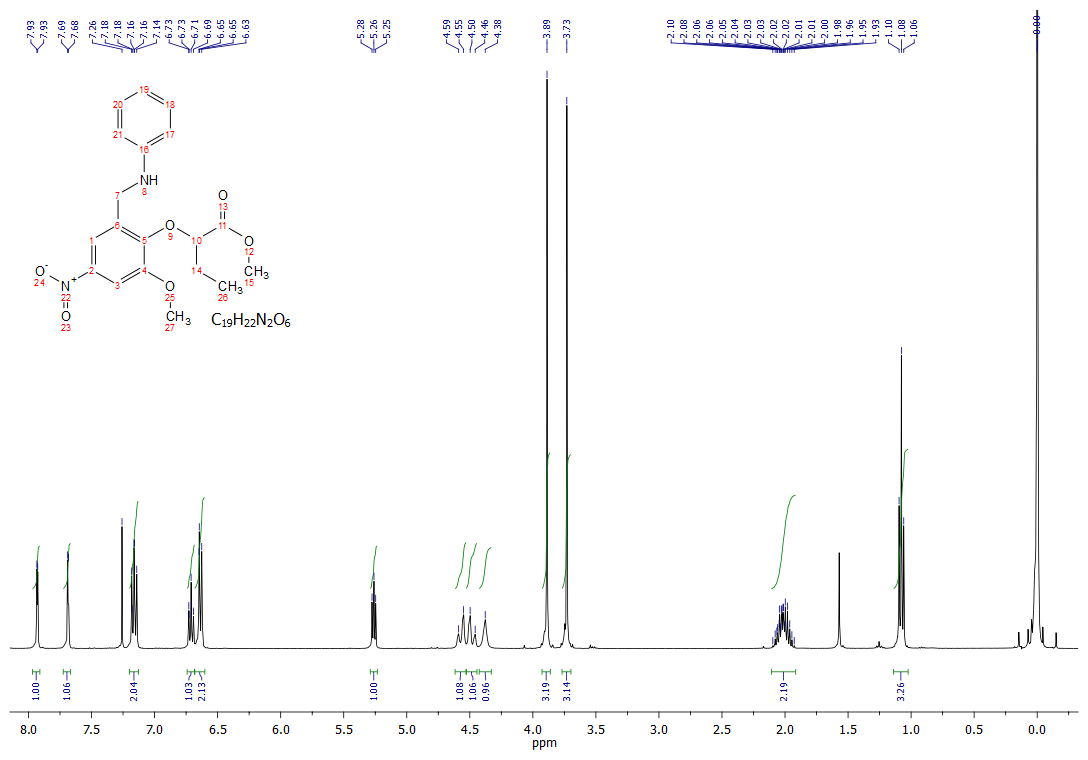** |

|  | **^13^C NMR** |
| --- | --- |
|  | **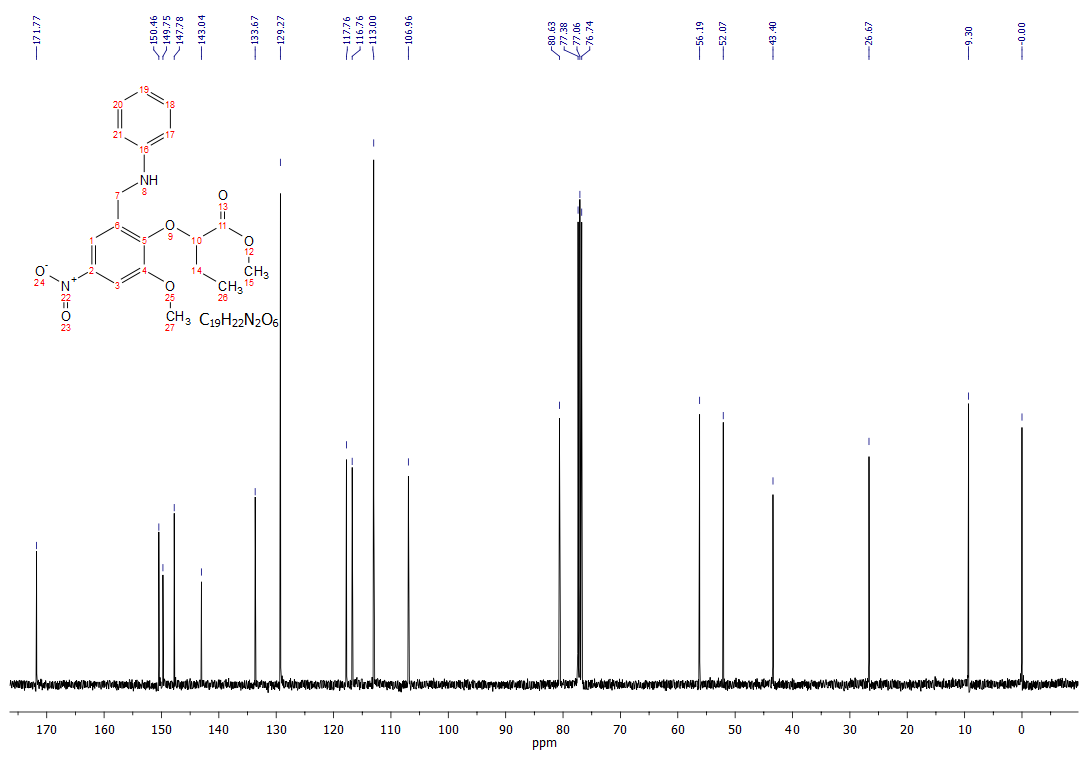** |

|  | **GC-MS** |
| --- | --- |
|  | **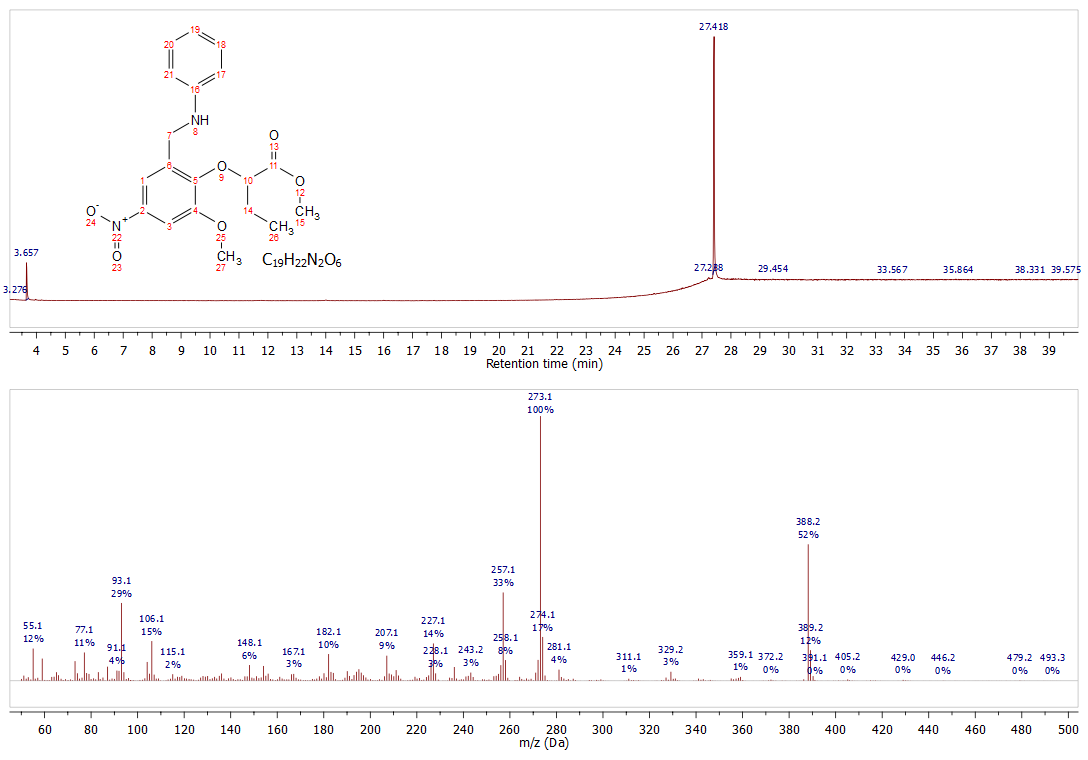** |

|  | **FTIR** |
| --- | --- |
|  | **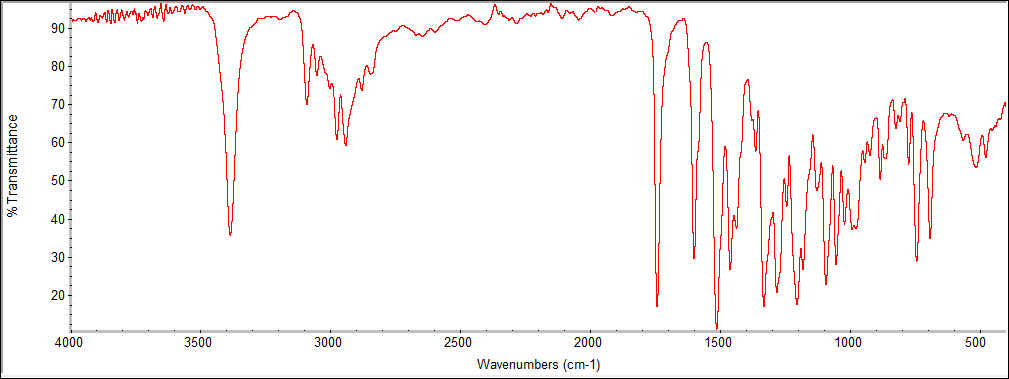** |

| **4e** | **Methyl 2-(2-methoxy-4-nitro-6-((phenylamino)methyl)phenoxy)pentanoate** |
| --- | --- |
|  | **^1^H NMR** |
|  | **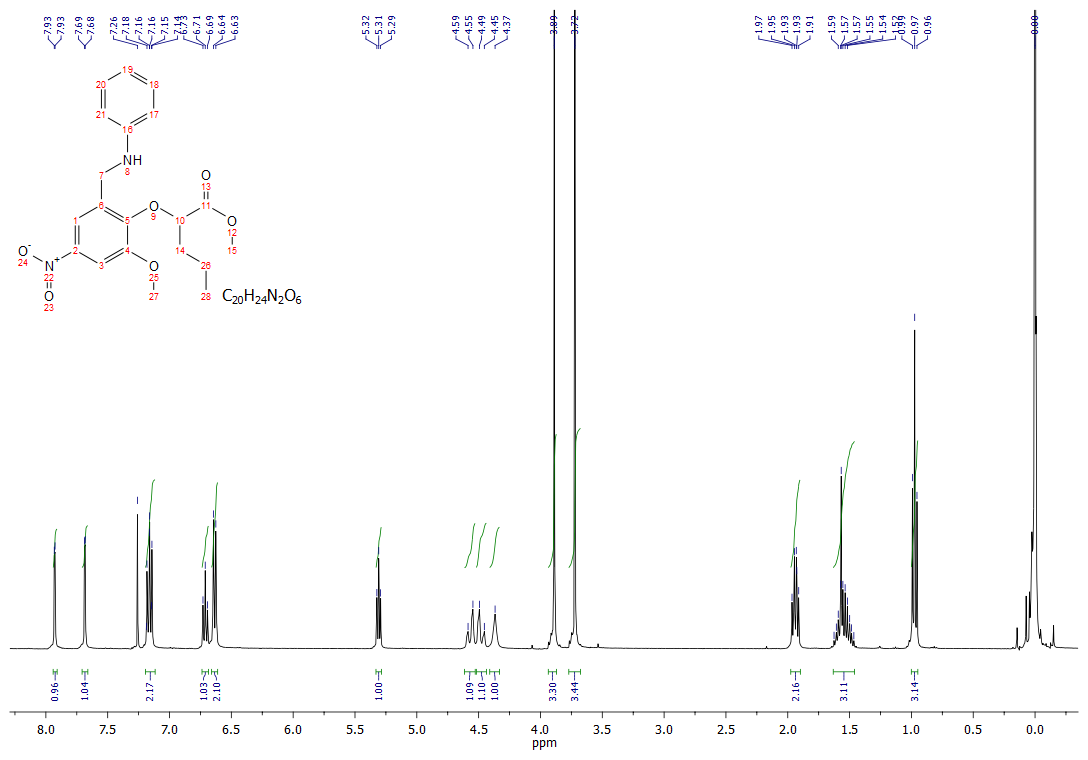** |

|  | **^13^C NMR** |
| --- | --- |
|  | **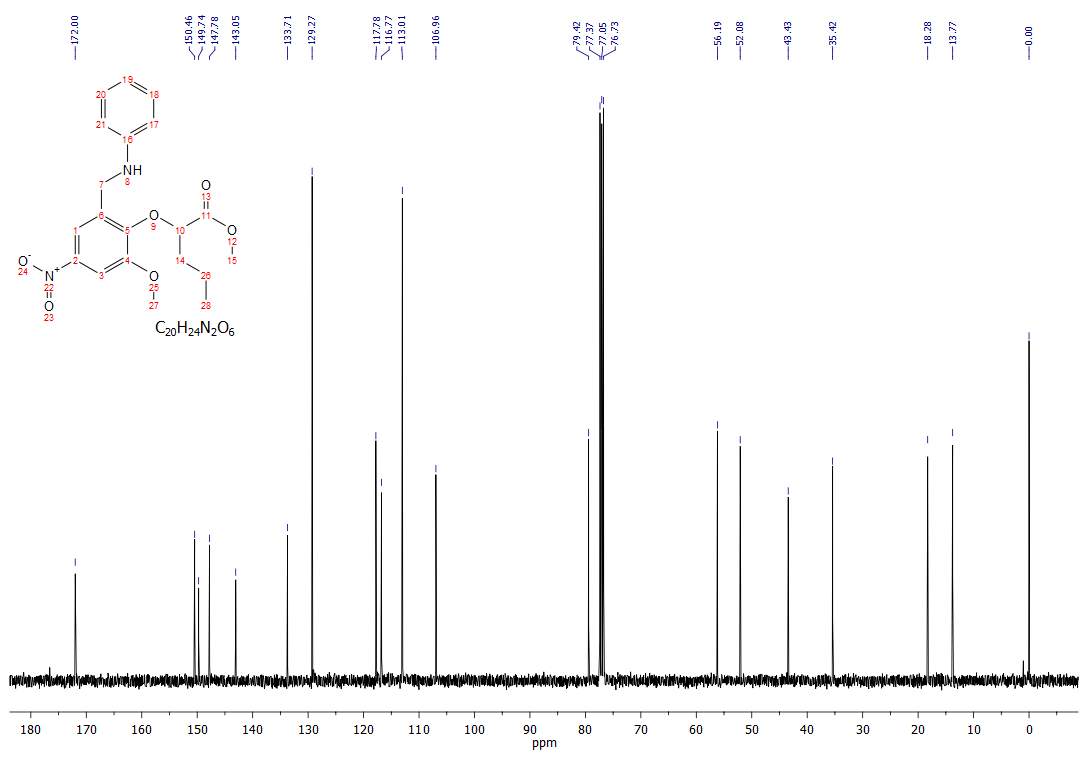** |

|  | **GC-MS** |
| --- | --- |
|  | **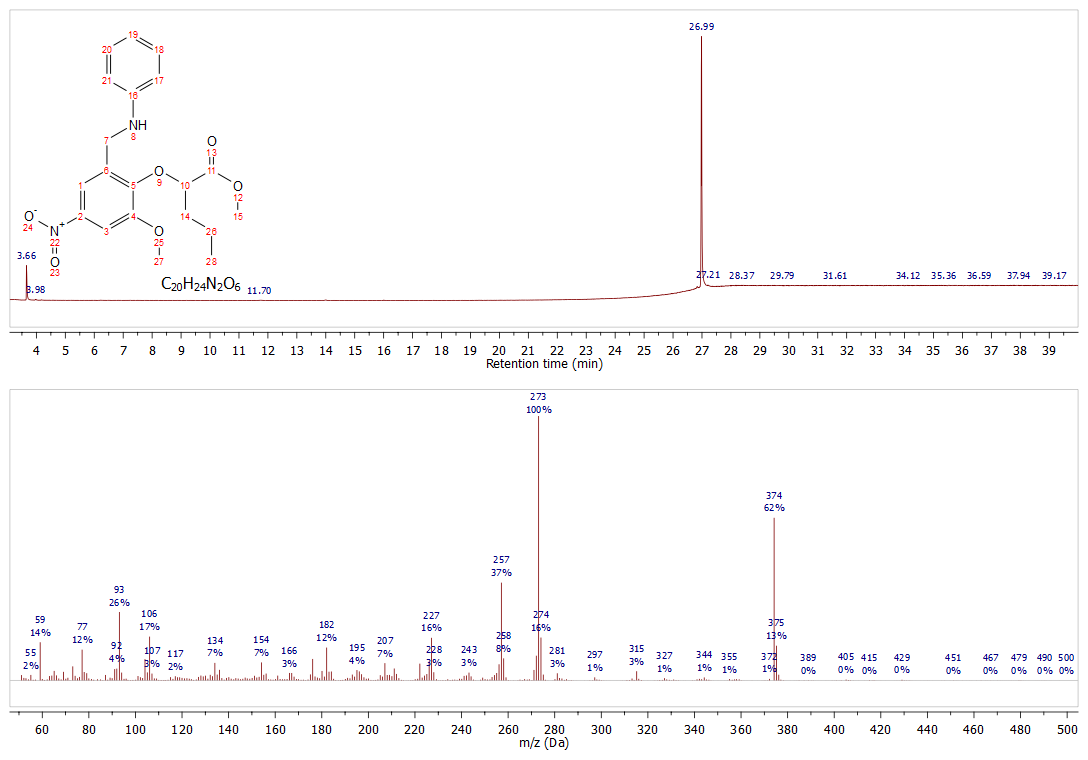** |

|  | **FTIR** |
| --- | --- |
|  | **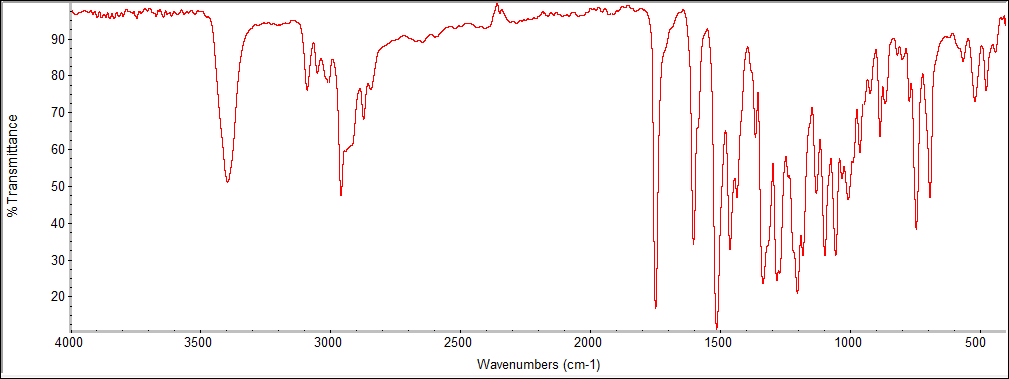** |

| **4f** | **Methyl 2-(2-methoxy-4-nitro-6-((phenylamino)methyl)phenoxy)hexanoate** |
| --- | --- |
|  | **^1^H NMR** |
|  | **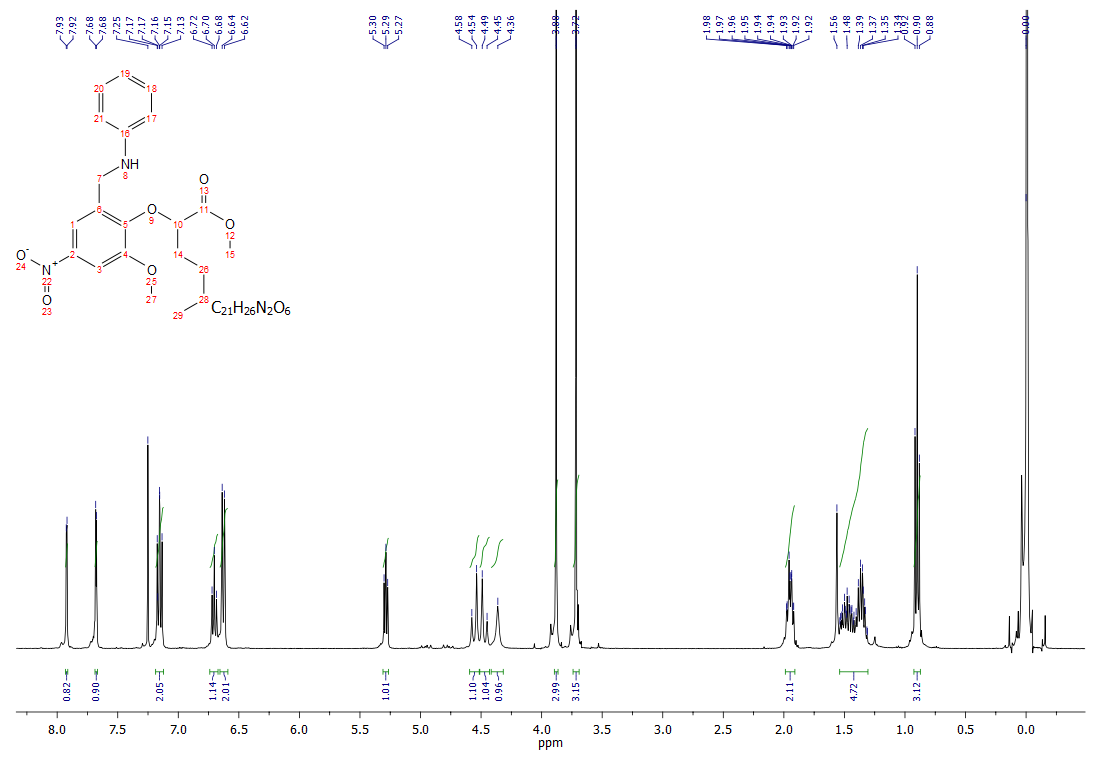** |

|  | **^13^C NMR** |
| --- | --- |
|  | **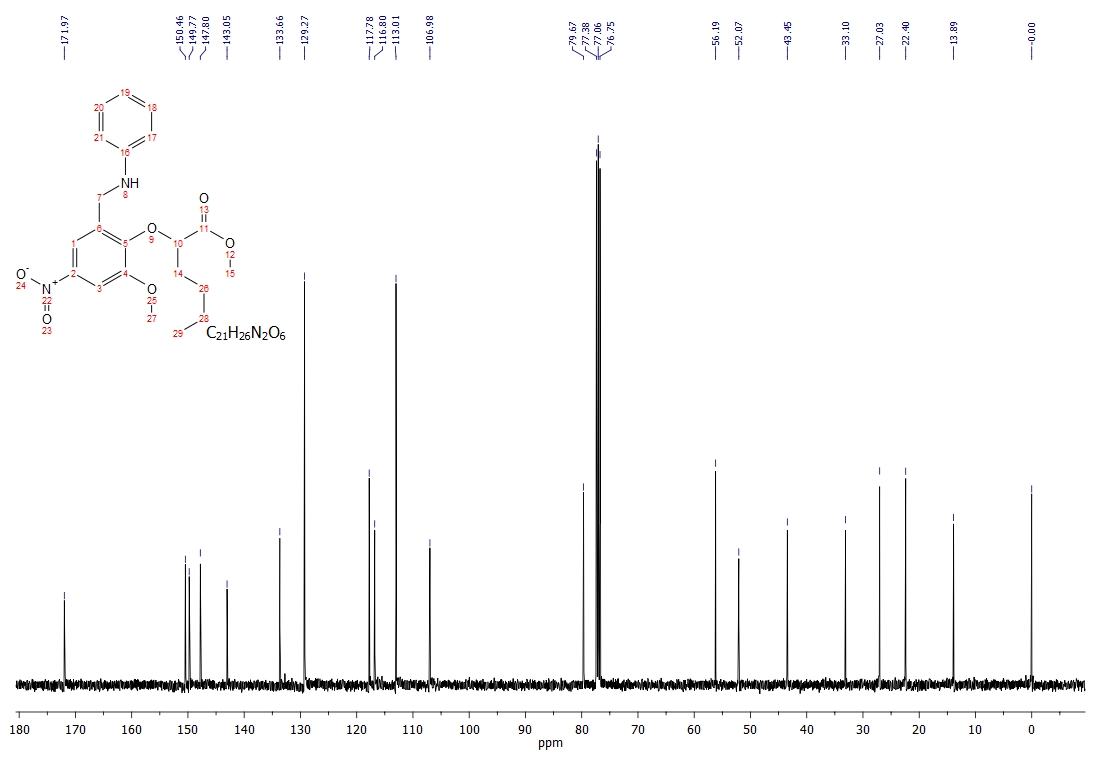** |

|  | **GC-MS** |
| --- | --- |
|  | **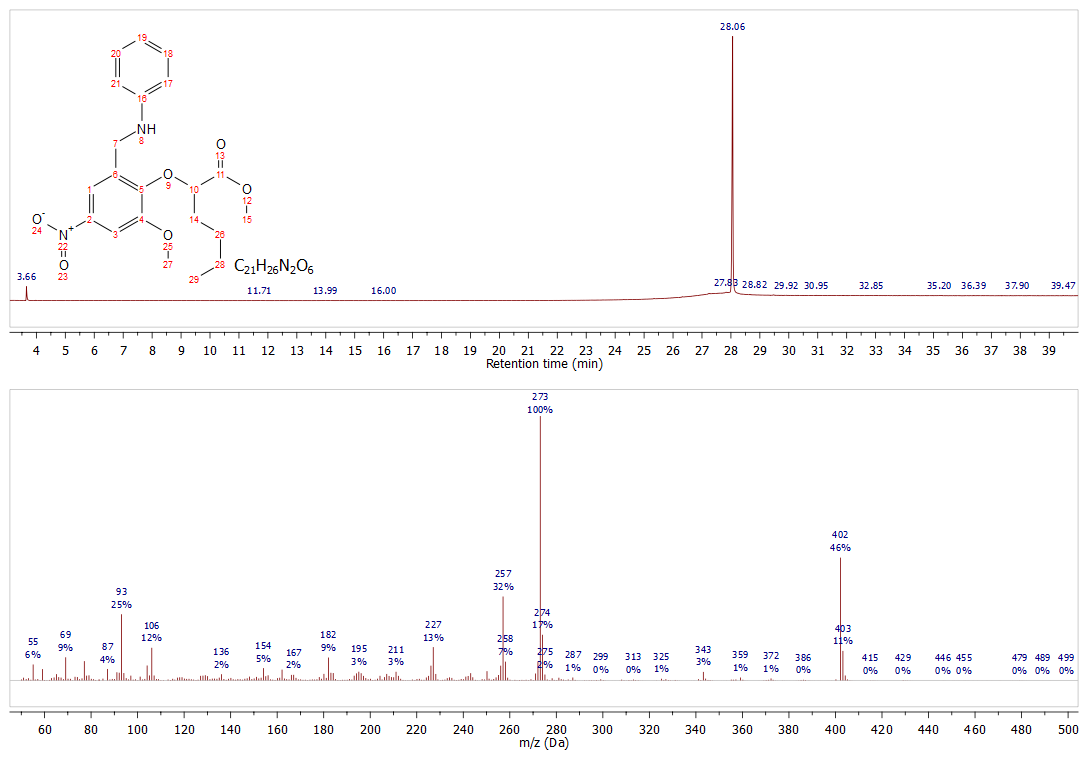** |

|  | **FTIR** |
| --- | --- |
|  | **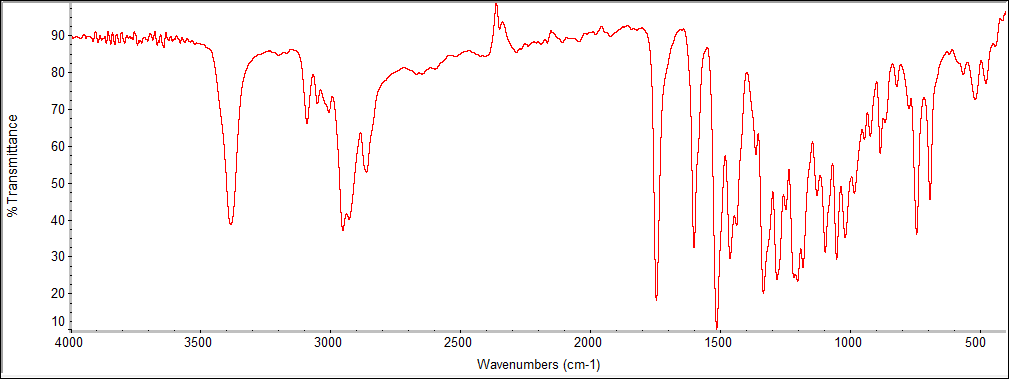** |

| **5a** | **Methyl 2-(4-amino-2-((phenylamino)methyl)phenoxy)butanoate** |
| --- | --- |
|  | **^1^H NMR** |
|  | **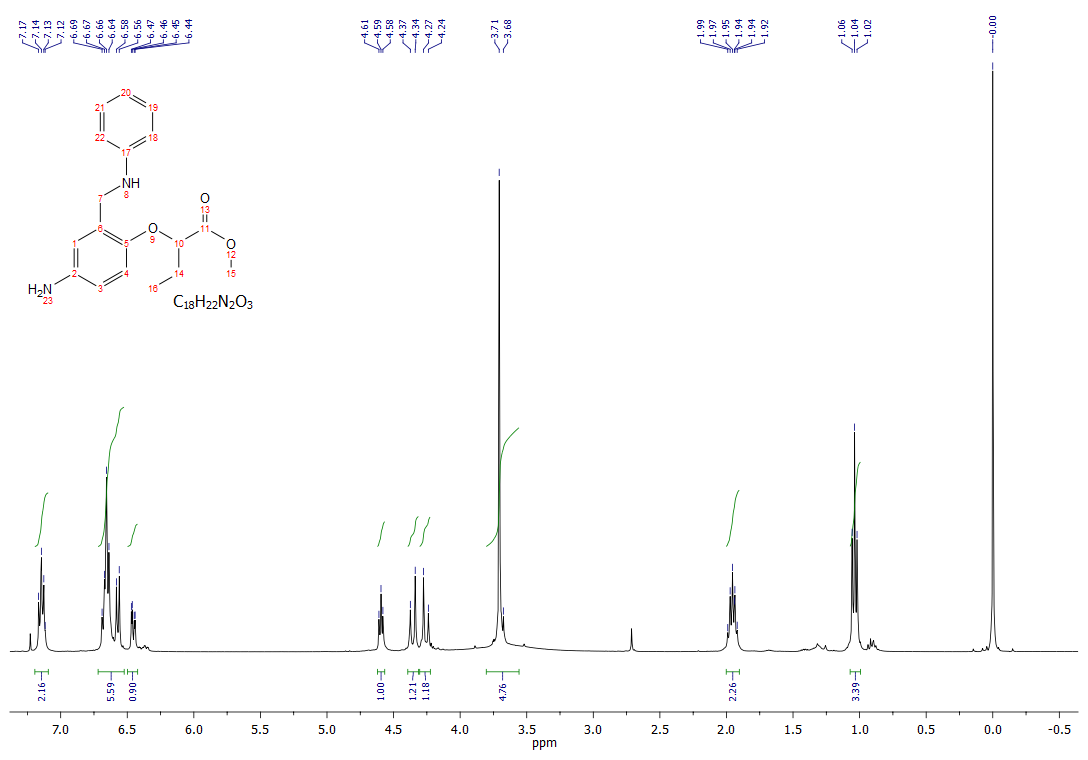** |

|  | **^13^C NMR** |
| --- | --- |
|  | **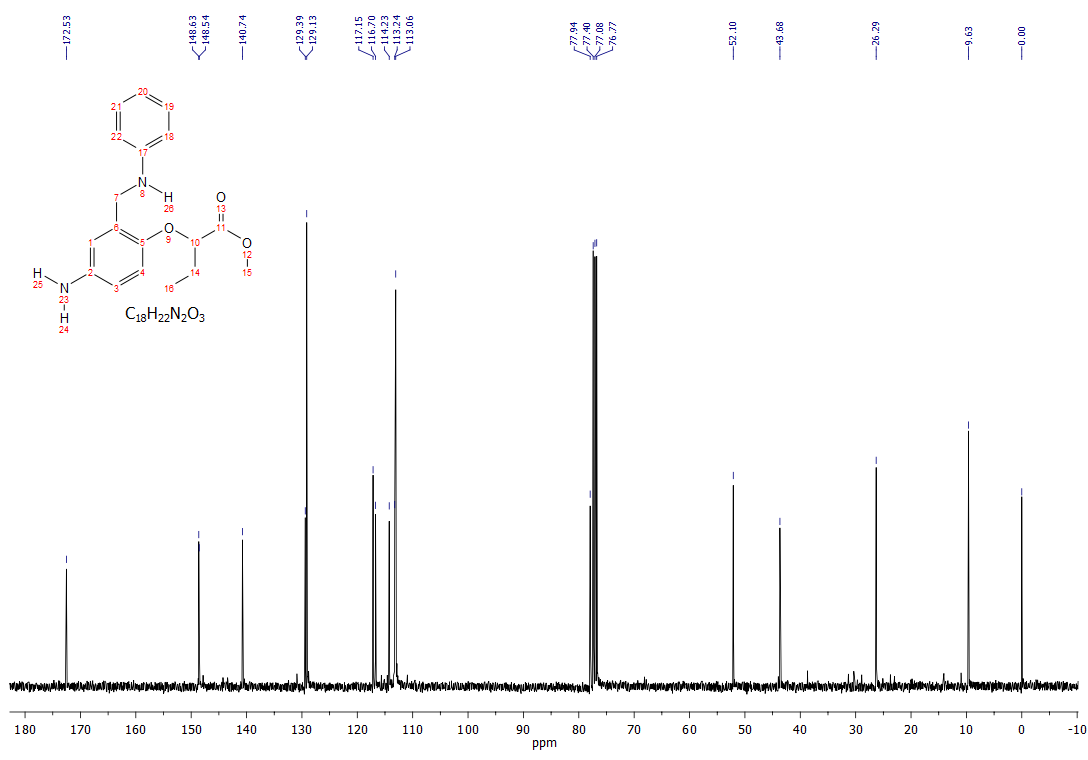** |

|  | **GC-MS** |
| --- | --- |
|  | **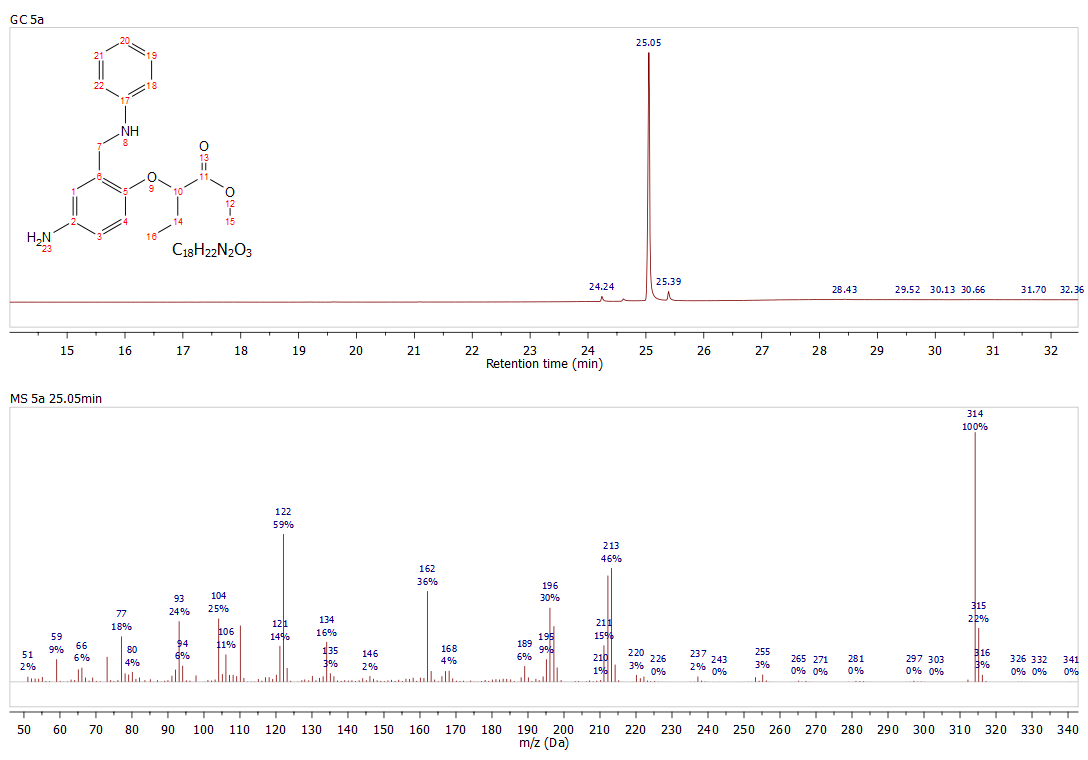** |

|  | **FTIR** |
| --- | --- |
|  | **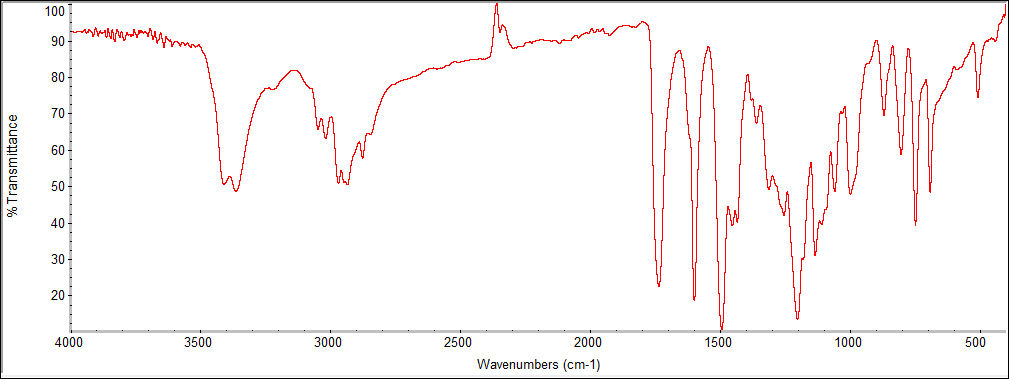** |

| **5c** | **Methyl 2-(4-amino-2-((phenylamino)methyl)phenoxy)hexanoate** |
| --- | --- |
|  | **^1^H NMR** |
|  | **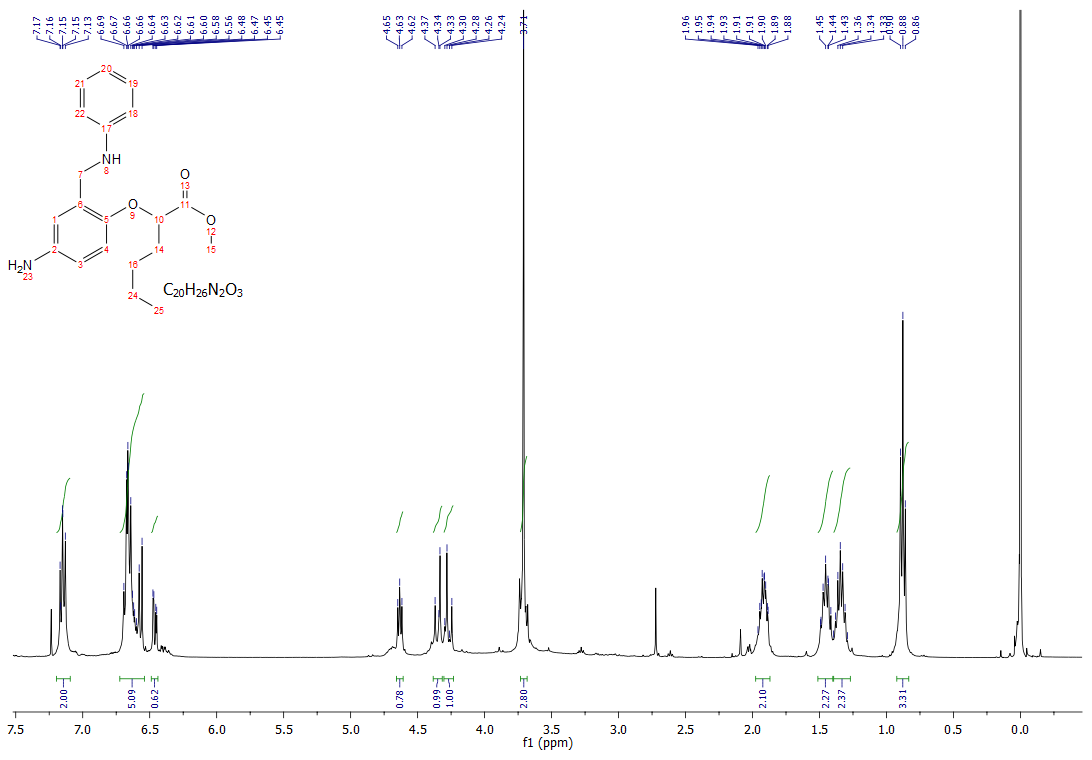** |
|  | **^13^C NMR** |
|  | **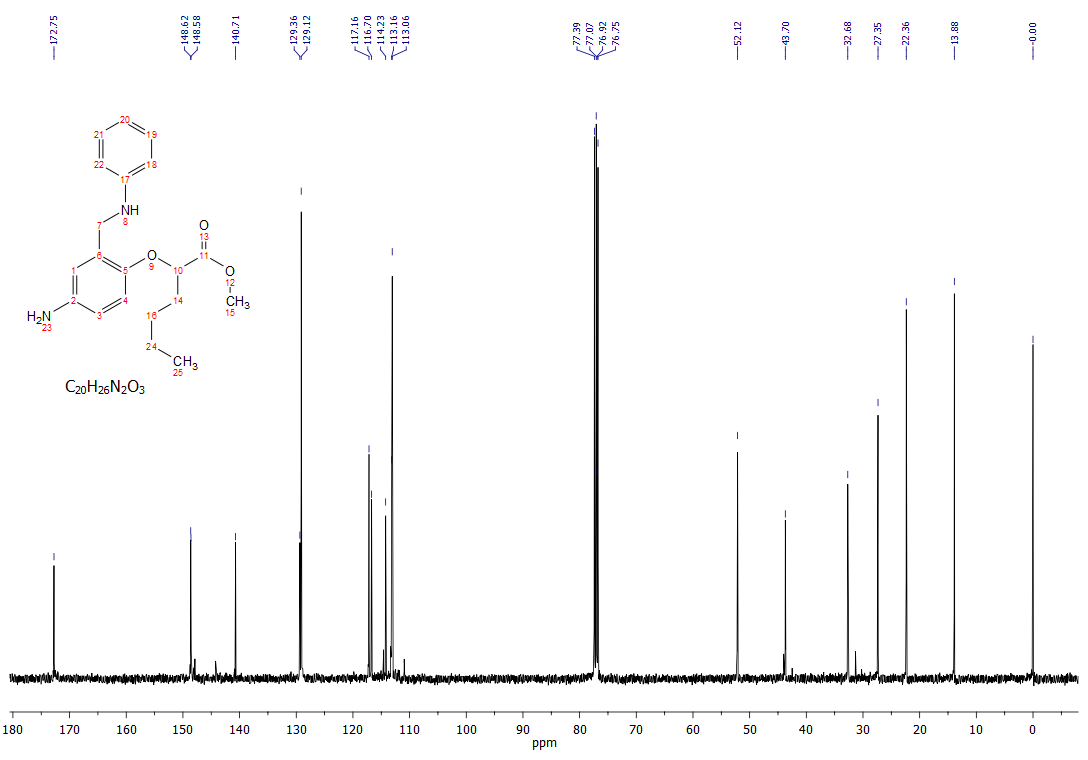** |

|  | **GC-MS** |
| --- | --- |
|  | **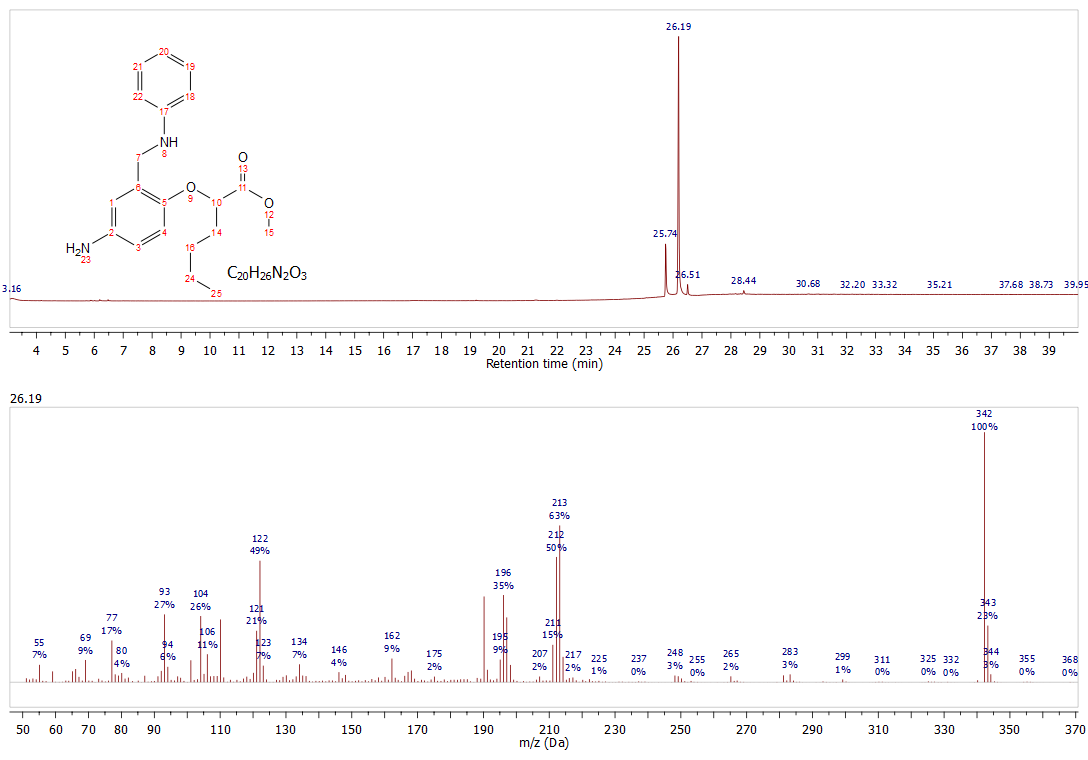** |

|  | **FTIR** |
| --- | --- |
|  | **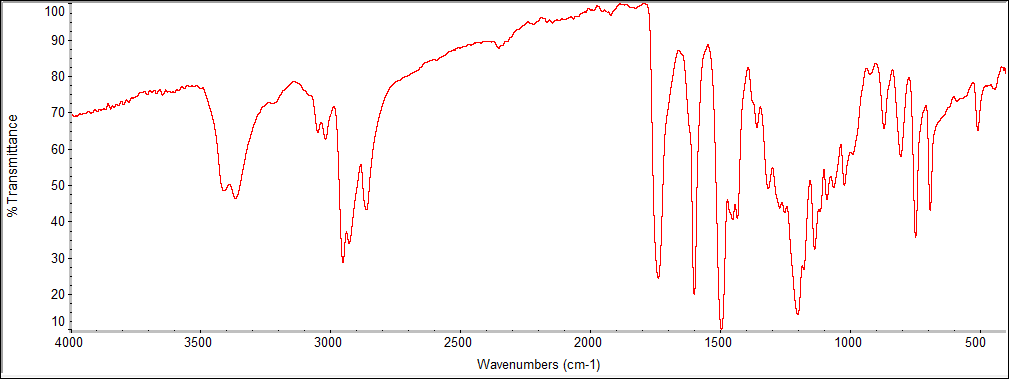** |

| **5d** | **Methyl 2-(4-amino-2-methoxy-6-((phenylamino)methyl)phenoxy)butanoate** |
| --- | --- |
|  | **^1^H NMR** |
|  | **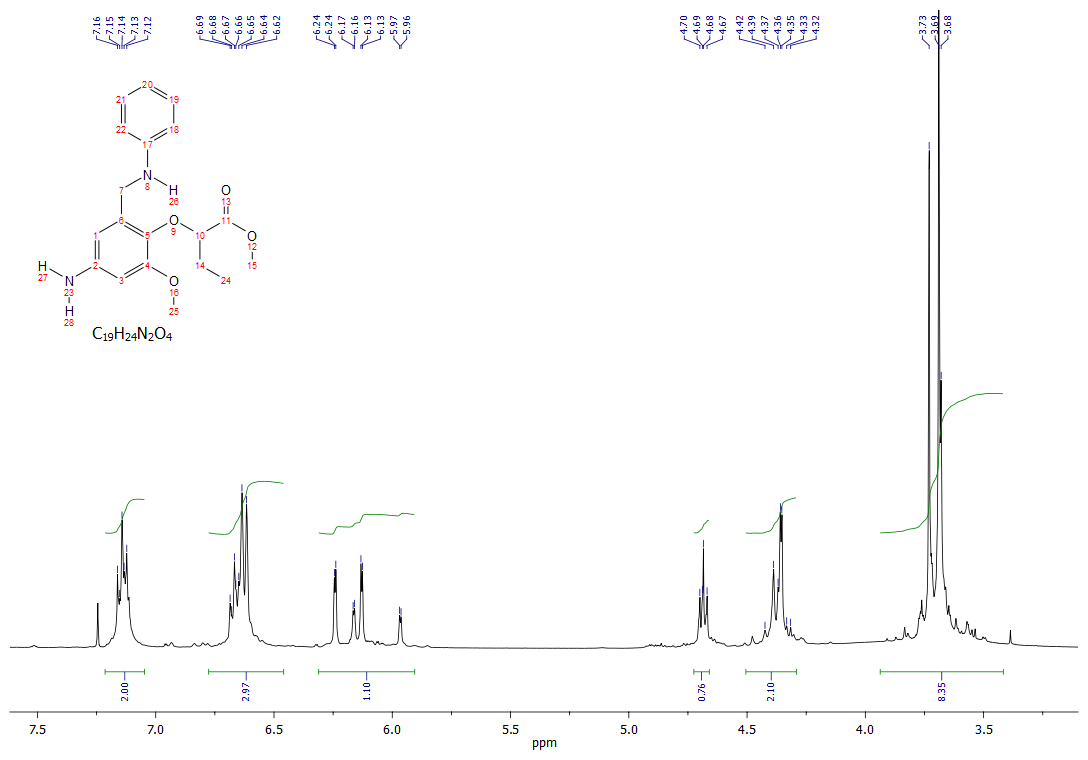** |

|  | **^13^C NMR** |
| --- | --- |
|  | **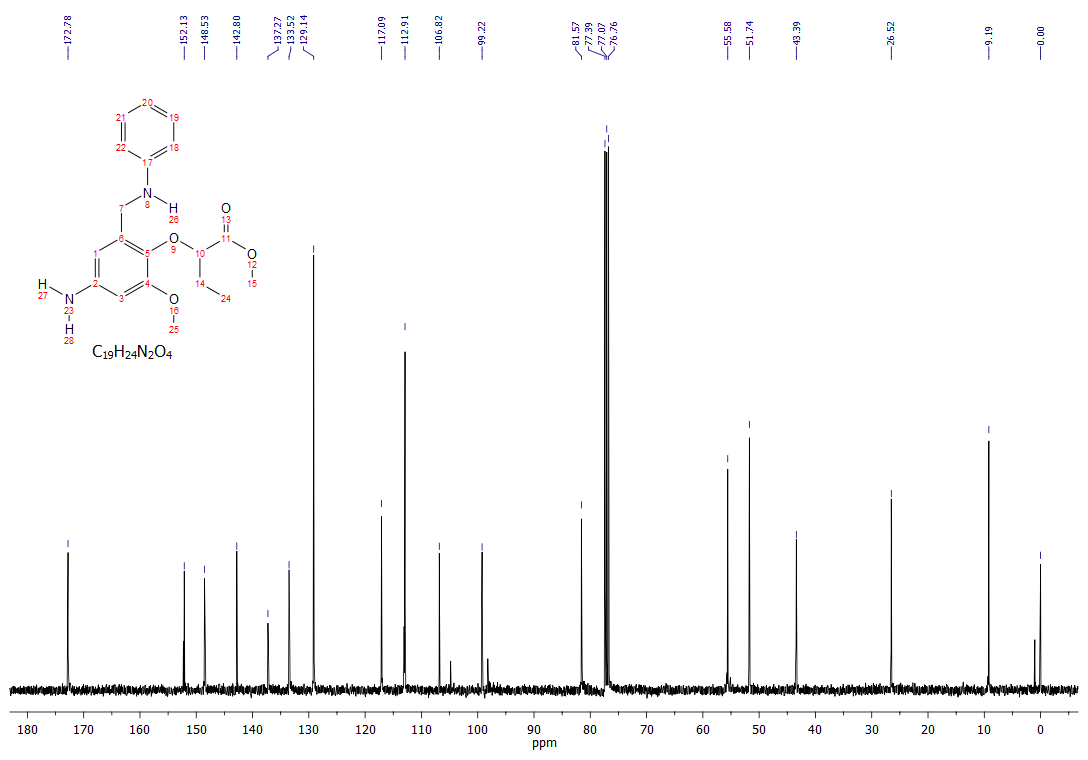** |

|  | **FTIR** |
| --- | --- |
|  | **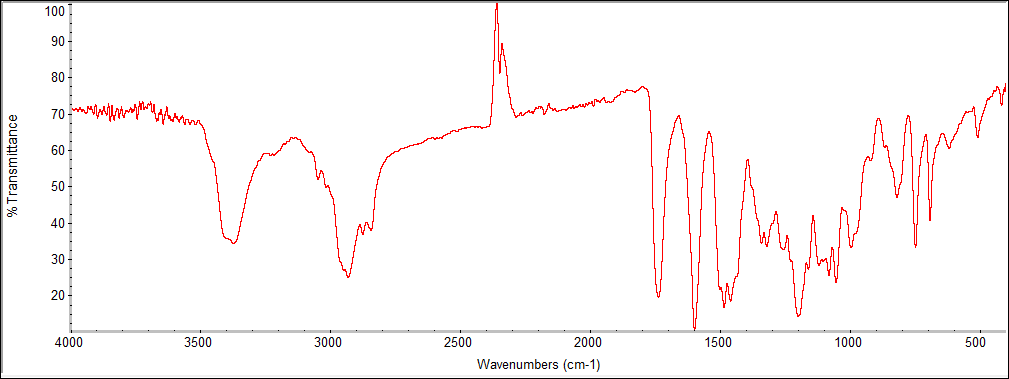** |

| **5e** | **Methyl 2-(4-amino-2-methoxy-6-((phenylamino)methyl)phenoxy)pentanoate** |
| --- | --- |
|  | **^1^H NMR** |
|  | **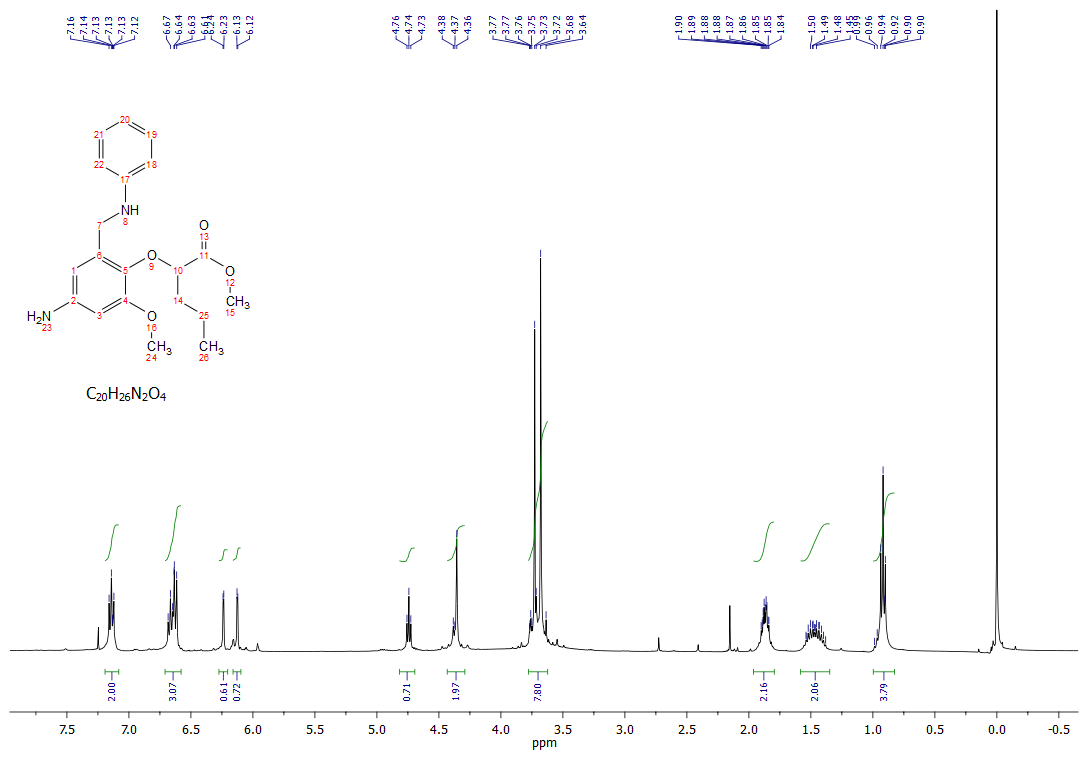** |

|  | **^13^C NMR** |
| --- | --- |
|  | **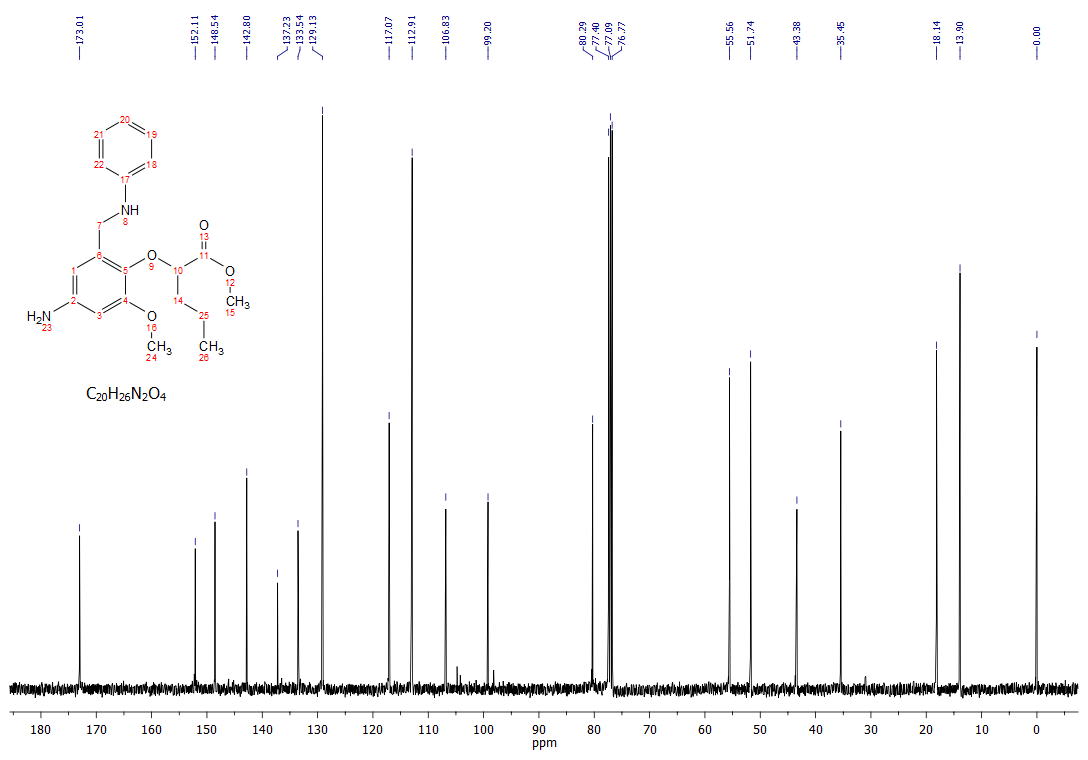** |

|  | **FTIR** |
| --- | --- |
|  | **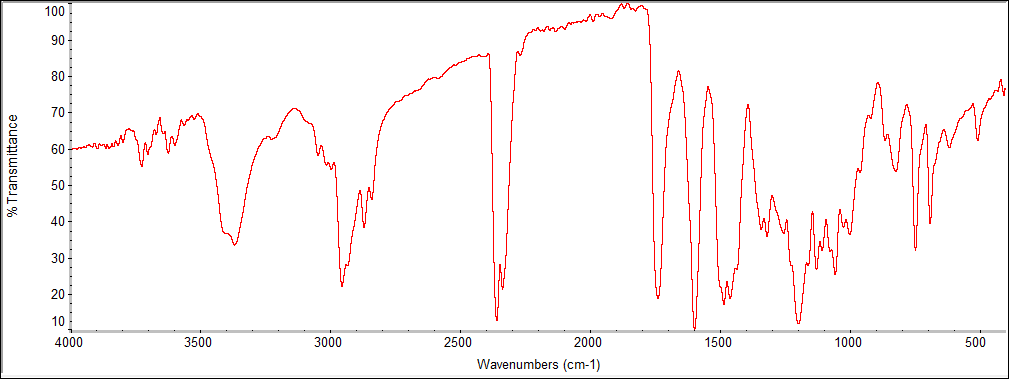** |

| **5f** | **Methyl 2-(4-amino-2-methoxy-6-((phenylamino)methyl)phenoxy)hexanoate** |
| --- | --- |
|  | **^1^H NMR** |
|  | **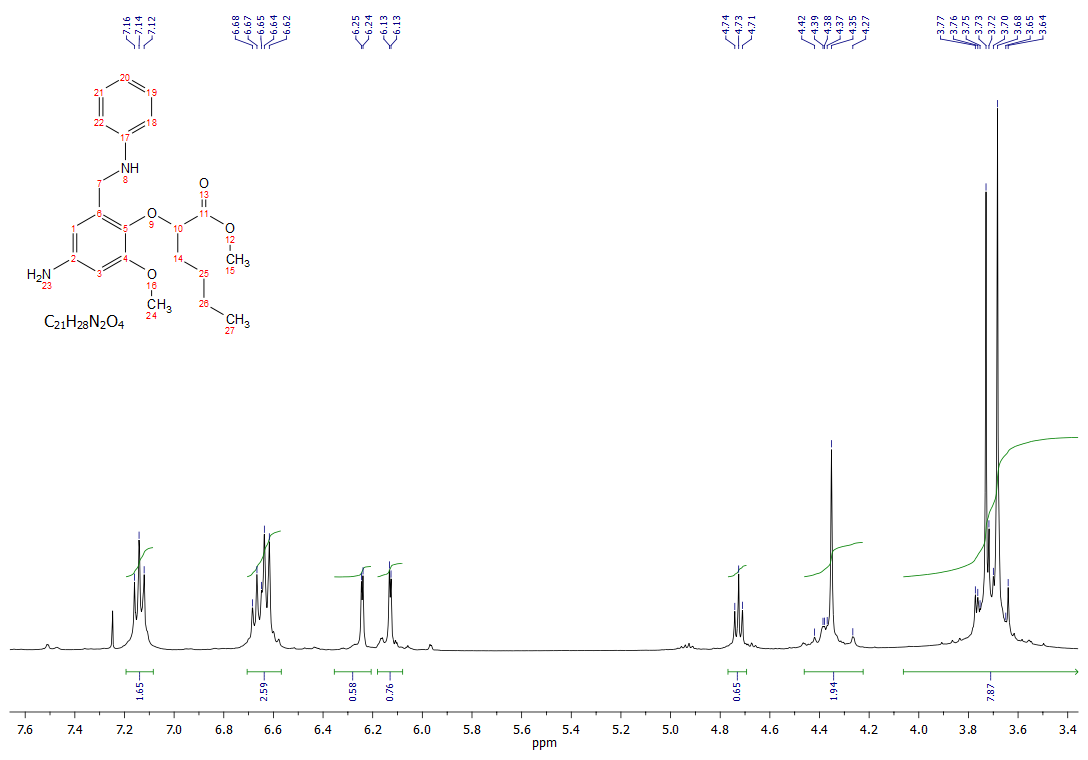** |

|  | **^13^C NMR** |
| --- | --- |
|  | **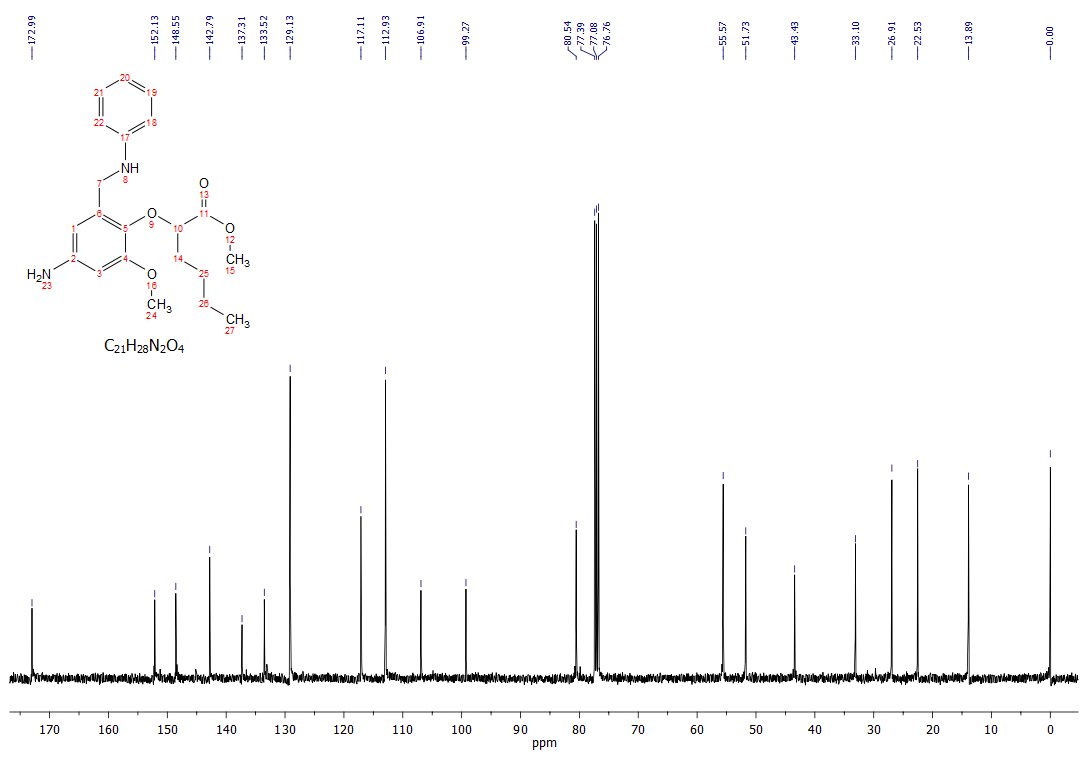** |

|  | **FTIR** |
| --- | --- |
|  | **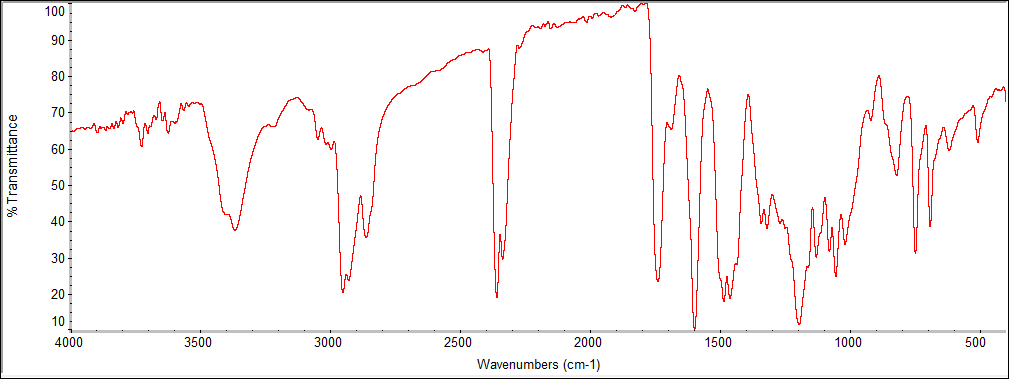** |
